# Associations of glucose- and lipid-lowering medications with incident or first-recorded psoriasis: a systematic review of real-world observational studies

**DOI:** 10.3389/fphar.2026.1942307

**Published:** 2026-10-14

**Authors:** Zhihong Li, Jie Yang, Danping Chen

**Affiliations:** 1 The Fourth Affiliated Hospital of Heilongjiang University of Chinese Medicine, Harbin, China; 2 Heilongjiang University of Chinese Medicine, Harbin, China

**Keywords:** glucose-lowering medications, incident psoriasis, lipid-lowering medications, pharmacoepidemiology, psoriasis, real-world evidence, systematic review

## Abstract

**Background:**

Glucose-lowering and lipid-lowering medications may influence psoriasis development through metabolic, inflammatory, and immune pathways, but real-world evidence remains inconsistent.

**Objective:**

To systematically evaluate associations between these medications and the risk of incident or first-recorded psoriasis, with particular attention to comparator choice, outcome definition, study design, and data-source overlap.

**Methods:**

PubMed, Embase, Web of Science Core Collection, and the Cochrane Library were searched from inception to 1 July 2026, with an updated search conducted on 11 September 2026 during manuscript revision. Longitudinal observational studies evaluating glucose-lowering or lipid-lowering medication use and incident or first-recorded psoriasis were included. Evidence was grouped by medication class, comparator, and outcome type and synthesized narratively. Psoriasis-specific outcomes and broader or composite psoriatic-disease outcomes were treated as distinct evidence categories. Risk of bias was assessed using ROBINS-I. Meta-analysis was not performed because sufficiently comparable and mutually independent estimates were unavailable within most medication–comparator–outcome strata.

**Results:**

Eighteen reports were included. Dipeptidyl peptidase-4 inhibitor estimates were generally below 1; in an active-comparator study using a broader psoriasis-related outcome including psoriatic arthritis codes, the hazard ratio versus sulfonylureas was 0.422 (95% confidence interval, 0.273–0.716). Glucagon-like peptide-1 receptor agonist estimates were notably comparator dependent: estimates were above 1 in comparisons with dipeptidyl peptidase-4 inhibitors, approximately null in comparison with sodium-glucose cotransporter-2 inhibitors, and inconsistent across comparisons with GLP-1RA nonuse. Most sodium-glucose cotransporter-2 inhibitor estimates were near or above the null. Metformin and thiazolidinediones yielded lower estimates in some studies, whereas statins showed no consistent association or exposure-response gradient. Of the 18 report-level ROBINS-I assessment anchors, 13 (72.2%) had a serious overall risk of bias and five (27.8%) had a moderate risk; none had a low risk of bias. Several reports may also have included overlapping populations and were therefore not interpreted as independent replications.

**Conclusion:**

The available observational evidence does not establish causal effects of glucose-lowering or lipid-lowering medications on incident or first-recorded psoriasis. This review does not support choosing or avoiding these medications solely to prevent psoriasis or modifying standard diabetes or cardiovascular treatment on the basis of the reported associations. The findings remain hypothesis-generating because of confounding by indication, other risks of bias, and limited independent evidence.

**Systematic Review Registration:**

https://doi.org/10.37766/inplasy2026.7.0070, identifier INPLASY202670070.

## 1 Introduction

Psoriasis is a chronic immune-mediated inflammatory disease that affects the skin and may also involve the joints and multiple systemic pathways. Dysregulation of the interleukin-23/T helper 17 axis plays a central role in its pathogenesis and contributes to its associations with systemic inflammation and cardiometabolic comorbidities (Armstrong et al., 2025). Psoriasis occurs worldwide and represents a substantial population-level health burden (Parisi et al., 2020). Epidemiologic evidence further indicates close associations between psoriasis and obesity, diabetes, insulin resistance, dyslipidemia, and metabolic syndrome (Alajroush et al., 2024). Identifying modifiable factors that may influence psoriasis onset is therefore important for disease prevention and long-term comorbidity management.

Glucose-lowering and lipid-lowering medications are widely used in patients with metabolic disorders. Beyond their primary effects on glycemic and lipid control, some agents may influence inflammatory, metabolic, and immune pathways relevant to psoriasis (Pantazopoulos et al., 2025). Metformin, thiazolidinediones, dipeptidyl peptidase-4 inhibitors, glucagon-like peptide-1 receptor agonists, sodium-glucose cotransporter-2 inhibitors, and statins differ in their effects on body weight, insulin resistance, adipose tissue inflammation, oxidative stress, and immune signaling. However, the direction and magnitude of their associations with psoriasis risk remain uncertain.

A central challenge is distinguishing associations related to medication exposure from those related to the underlying conditions that lead to treatment. Diabetes, obesity, insulin resistance, dyslipidemia, cardiovascular disease, and chronic kidney disease are associated with psoriasis and may also influence whether glucose-lowering or lipid-lowering therapy is prescribed and which medication is selected. Consequently, observed medication–psoriasis associations may partly or entirely reflect differences in treatment indication, disease severity, and associated comorbidities. Confounding by indication is therefore central to the interpretation of this evidence.

Previous research has mainly examined whether glucose-lowering medications improve disease activity in patients with established psoriasis rather than whether they are associated with its initial occurrence. A previous meta-analysis of antidiabetic medications focused largely on clinical outcomes among patients who already had psoriasis (Chi et al., 2021). Reviews of glucagon-like peptide-1 receptor agonists have similarly emphasized changes in psoriasis severity, body weight, and related cardiometabolic outcomes (Haran et al., 2024). Existing dermatological literature on statins has primarily examined their potential effects on psoriasis severity in patients with established disease, including evidence from randomized clinical trials, rather than their association with the initial occurrence of psoriasis (Socha et al., 2020). Evidence concerning disease severity or treatment response and evidence concerning incident or first-recorded psoriasis address different clinical questions and should not be directly extrapolated from one to the other.

With the increasing availability of administrative healthcare databases, insurance claims data, and electronic health records, real-world studies evaluating metabolic medications and incident psoriasis have accumulated. However, these studies differ substantially in target medications, comparators, exposure definitions, time-zero specification, outcome ascertainment, and control of confounding. Active-comparator analyses, comparisons with heterogeneous treatment groups, and comparisons with medication non-users address different clinical questions. In addition, some reports may have used the same or partially overlapping database populations. Summarizing findings solely according to effect direction or statistical significance may therefore obscure important differences in study design and overstate the independence of the evidence base.

We systematically reviewed the associations of glucose-lowering and lipid-lowering medication use with incident or first-recorded psoriasis. Real-world longitudinal observational studies were grouped according to medication class, comparator, and outcome type. We assessed the risk of bias in report-level assessment anchors and examined potential overlap in data sources and study populations across reports. This review aimed to determine whether the available evidence supports consistent class-level associations and to clarify the extent to which observed findings depend on the specific comparator and study design.

## 2 Methods

### 2.1 Study design and protocol registration

This systematic review synthesized evidence from real-world longitudinal observational studies on the associations between glucose-lowering and lipid-lowering medication use and the risk of incident or first-recorded psoriasis. We also examined whether the findings varied according to medication class, comparator, outcome definition, and study design. The review was reported in accordance with the Preferred Reporting Items for Systematic Reviews and Meta-Analyses 2020 statement (PRISMA 2020) (Page et al., 2021). Evidence that was not considered suitable for quantitative pooling was grouped and narratively synthesized with reference to the Synthesis Without Meta-analysis guideline and the Cochrane Handbook for Systematic Reviews of Interventions (Campbell et al., 2020; McKenzie et al., 2024). The review protocol was registered with INPLASY (registration number: INPLASY202670070; DOI: 10.37766/inplasy2026.7.0070). The protocol was registered retrospectively on 19 July 2026, after completion of the initial review but before publication. Changes and clarifications relative to the registered protocol and the initial manuscript are documented in Supplementary Table S14. Changes introduced during revision are distinguished from methods already described in the registered protocol.

PubMed, Embase, Web of Science Core Collection, and the Cochrane Library were systematically searched from database inception to 1 July 2026. To ensure that the review remained current before manuscript resubmission, the database searches were updated on 11 September 2026 using the same eligibility framework. The update used the same search strategies as the initial search, with a publication-year restriction to 2026, and covered records available on 11 September 2026. It was not restricted to publications appearing after 1 July 2026. The same reference-management and screening procedures were used. Search strategies combined controlled vocabulary and free-text terms adapted to the indexing system and syntax of each database. The search covered psoriasis, glucose-lowering and lipid-lowering medications, major medication classes, and individual drug names, with no language restrictions. We also screened the reference lists of included reports and relevant systematic reviews to identify potentially eligible studies not retrieved through the database searches. The database-specific search strategies and initial retrieval counts are provided in Supplementary Table S1, together with a description of the revision-stage update. The revision-stage search date and screening results are reported under “Updated literature search” in the Supplementary Material and in Supplementary Figure S7.

### 2.2 Eligibility criteria and study selection

We included longitudinal observational studies according to predefined eligibility criteria to evaluate the association between glucose-lowering or lipid-lowering medication use and either incident psoriasis or first-recorded psoriasis. The eligible populations mainly included individuals receiving treatment for diabetes, dyslipidemia, or related metabolic conditions in routine healthcare settings. For this review, incident psoriasis referred to a newly diagnosed outcome during follow-up in studies that explicitly excluded previously recorded psoriasis before baseline or during a specified pre-index period. This operational definition did not establish the timing of biological disease onset. First-recorded psoriasis referred to the first psoriasis diagnosis observed in the available healthcare data, without assuming that the recording date represented disease onset. Even after exclusion of prior recorded diagnoses, previously existing but undocumented psoriasis could remain. Delayed recognition and differences in healthcare contact could therefore influence the observed associations. When the exclusion of pre-existing psoriasis was insufficiently described, this uncertainty was retained in the interpretation.

Eligible exposures were clearly identifiable glucose-lowering or lipid-lowering medications and could be defined as treatment initiation, current or previous use, cumulative exposure, frequency of use, or medication adherence. Comparators were categorized as active medication comparators, heterogeneous treatment populations, medication non-users, or groups defined by different exposure durations, intensities, or adherence levels. Eligible outcomes included psoriasis-specific incident psoriasis, psoriasis-specific first-recorded psoriasis, broader psoriasis-related outcomes, and composite psoriasis/psoriatic arthritis outcomes. Outcome ascertainment could be based on clinical diagnoses, electronic health records, diagnostic classification codes, insurance claims, or case-identification algorithms. These outcome categories were retained separately in the evidence synthesis and were not considered interchangeable. Studies reporting only a composite psoriasis/psoriatic arthritis outcome were eligible for inclusion, but their estimates were synthesized separately from separately reported psoriasis outcomes. Composite outcomes were not interpreted as evidence specific to cutaneous psoriasis. Eligible study designs included cohort, case-control, and nested case-control studies. Studies were required to report a hazard ratio, odds ratio, or risk ratio with a 95% confidence interval, or to provide sufficient data to calculate a corresponding effect estimate.

We excluded cross-sectional and ecological studies, case reports, case series, animal or cellular studies, reviews, commentaries, editorials, and study protocols. Studies that evaluated only psoriasis severity, treatment response, or improvement of established psoriatic lesions were also excluded. Reports were considered ineligible when the specific medication, comparator, or eligible psoriasis outcome could not be identified; an effect estimate could not be extracted or calculated; or the report represented a duplicate publication of an included study without providing additional relevant information.

All retrieved records were imported into NoteExpress for reference management and deduplication. Duplicate records were removed before title and abstract screening. Two reviewers (Z.H.L. and J.Y.) independently screened titles and abstracts and subsequently assessed the eligibility of potentially relevant full-text reports according to the predefined eligibility criteria. Disagreements regarding study selection were resolved through discussion between the two reviewers, with adjudication by a third reviewer (D.P.C.) when consensus could not be reached. The study selection process is shown in Figure 1.

**FIGURE 1:**
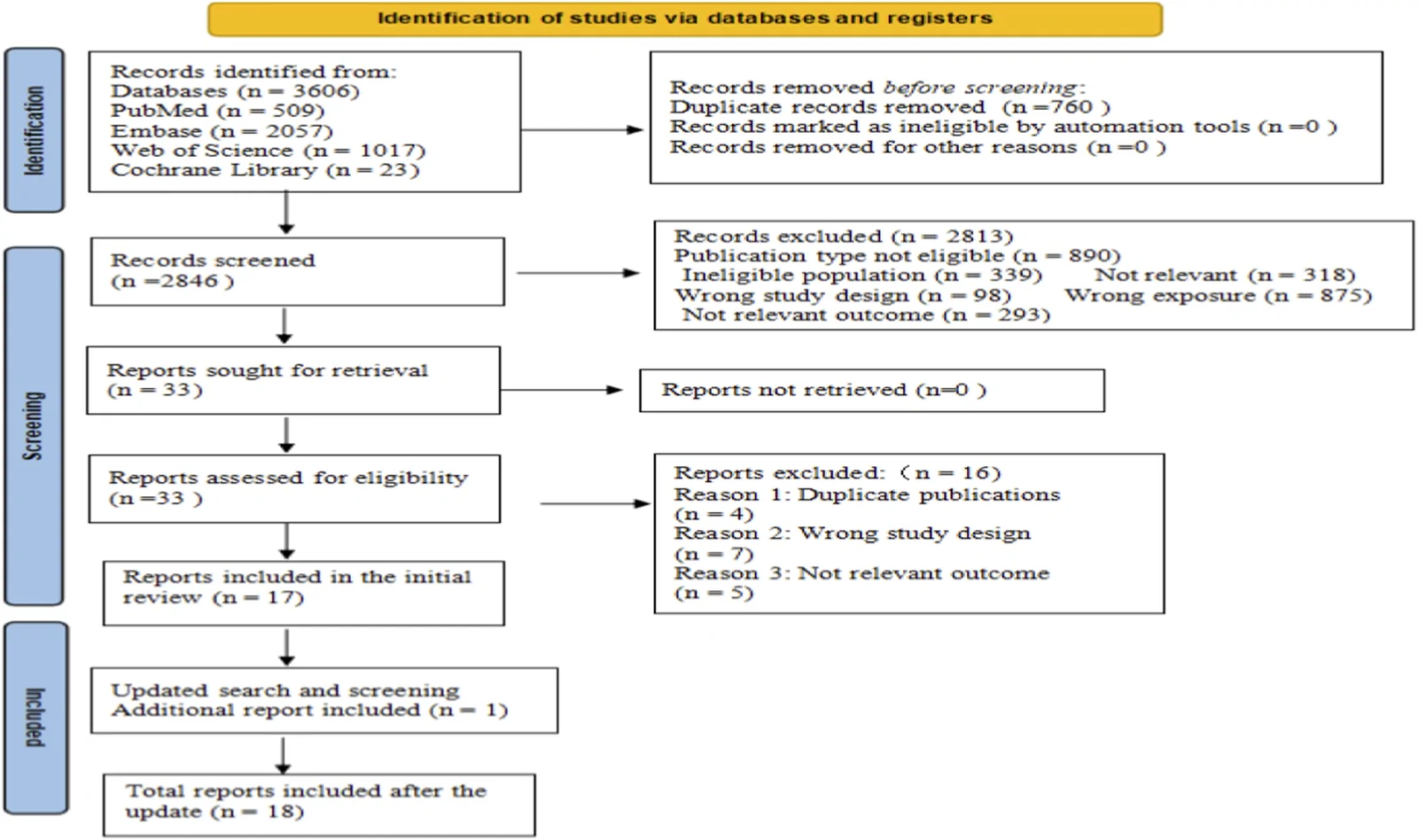
PRISMA flow diagram of the initial search and summary of the revision-stage update. Seventeen reports were included initially, and one additional report was included after the update, yielding 18 reports in total. Details of the updated search are presented in Supplementary Figure S7.

### 2.3 Data extraction

Two reviewers (Z.H.L. and J.Y.) independently extracted data using a prespecified, pilot-tested form and cross-checked all entries. Any disagreement was resolved by reviewing the original report and discussing the discrepancy, with adjudication by a third reviewer (D.P.C.) when necessary. Extracted information included author, publication year, country or region, data source, study design, study period, study population, sample size, number of outcome events, medication exposure, comparator, exposure and outcome definitions, effect measure, effect estimate and 95% confidence interval, statistical methods, and controlled confounders.

Available outcome events, analytic denominators, person-time, and reported absolute measures are summarized in Supplementary Table S12. Counts and denominators were linked to the corresponding comparison and analysis. Reported risks, survival-based cumulative incidences, and incidence rates were not treated as interchangeable. Case-control exposure frequencies were not converted into incidence risks. Where person-time was reported, any review-calculated crude incidence rate is explicitly identified; adjusted absolute risks were not reconstructed from HRs or ORs.

For studies using new-user, active-comparator, target trial emulation, or landmark designs, we additionally extracted the index date or time zero, baseline or washout period, exposure assessment window, outcome lag period, rules for the beginning and end of follow-up, and censoring criteria. Study design, time zero, and follow-up strategies are summarized in Supplementary Table S4A; confounding control and statistical methods are presented in Supplementary Table S4B; and exposure, comparator, and outcome definitions are provided in Supplementary Table S5.

Outcome selection was restricted to outcomes relevant to this review. An outcome designated as primary by the original investigators was not automatically preferred if it addressed a different disease or a broader composite endpoint. When both a separately reported psoriasis outcome and a broader or composite psoriatic-disease outcome were available, priority for the main interpretation was given to the psoriasis outcome, with its diagnostic specificity assessed from the available coding information. Each extracted outcome was characterized along two dimensions: its temporal ascertainment framework (study-defined incident disease or first-recorded diagnosis, with uncertainty retained where reporting was insufficient) and its diagnostic scope (cutaneous psoriasis, a broader psoriasis-related coding definition, a composite psoriasis/psoriatic arthritis outcome, or unconfirmed specificity). Incident psoriasis and first-recorded psoriasis were retained as distinct outcome categories and were not treated as synonymous. Broader psoriasis-related and composite psoriasis/psoriatic arthritis outcomes were extracted and presented separately from psoriasis-specific outcomes. When the available diagnostic coding information did not establish whether an outcome was restricted to cutaneous psoriasis, psoriasis specificity was recorded as unconfirmed. Such uncertainty was retained in the evidence tables and interpretation rather than resolved by assumption. Similarly, when several statistical models were reported, we selected the primary adjusted model specified by the original study or, if none was identified, the model that most closely addressed the review question and most adequately controlled for key baseline confounders. We cross-checked the direction of comparison, reference group, analytic population, outcome classification, and effect estimate across the abstract, main text, tables, figures, and supplementary materials. Inconsistencies that could not be resolved from the original publication were recorded in Supplementary Table S10 and considered when interpreting the findings.

### 2.4 Evidence grouping, ROBINS-I assessment anchors, synthesis-result selection, and management of overlapping populations

Effect estimates were grouped according to the target medication, comparator, and outcome type. Outcome strata were kept distinct as psoriasis-specific incident psoriasis, psoriasis-specific first-recorded psoriasis, broader psoriasis-related outcomes, and composite psoriasis/psoriatic arthritis outcomes. Incident psoriasis and first-recorded psoriasis were not treated as synonymous or directly interchangeable. Psoriasis-specific estimates were prioritized when available for the same medication comparison. Representative estimates were interpreted within their stated medication–comparator–outcome context. A representative estimate based on a broader outcome, or on an outcome with unconfirmed psoriasis specificity, was not interpreted as evidence specific to cutaneous psoriasis. During revision, outcome classifications were corrected or qualified following re-examination of the available coding information; effect estimates were retained as reported. Active-comparator analyses, comparisons with heterogeneous or non-active treatment groups, comparisons with medication non-users, treatment-strategy comparisons, and analyses according to exposure duration, intensity, or adherence were organized and interpreted separately. When a report provided several effect estimates, the result selected for primary interpretation was based on the clarity of the comparator, appropriateness of time zero, specificity and ascertainment of the outcome, and adequacy of confounding control. Results from active-comparator new-user designs were prioritized when comparing alternative treatment strategies. By comparing initiators of the target medication with initiators of a clinically relevant alternative treatment, this design may improve comparability in treatment indication and disease stage and align baseline assessment with the start of follow-up, although residual confounding cannot be completely excluded (Lund et al., 2015).

During revision, we distinguished the specific result used to anchor a ROBINS-I record from the results used to interpret a medication–comparator–outcome contrast. An assessment anchor identifies a population, exposure definition, comparator, outcome and model; it does not establish that the result is the primary result of the original report or that it should dominate the synthesis. The reasons for the original choice of individual anchors were not documented contemporaneously. These historical limitations remain, and the revision-stage rules described here are not presented as prespecified methods.

For each medication–comparator–outcome contrast, we prioritized the original report’s main adjusted analysis in the full eligible population, retaining its stated treatment strategy and follow-up horizon. When no main model was designated, we considered control of relevant baseline confounding and avoidance of adjustment for post-treatment variables. Separate psoriasis outcomes were preferred to broader composites only within the same clinical comparison; uncertain cutaneous specificity was retained. Where there was no naturally preferred sex, prescription-count or duration stratum, all relevant strata were presented in parallel. We did not select a stratum because of its effect direction, magnitude, statistical significance or precision. Different comparators and outcome categories were not substituted for one another.

Accordingly, we considered both sexes and all non-reference PDC categories in Chodick, both current-use TZD prescription strata and the separate past-use strata in Brauchli 2008, and both any-statin duration strata in Han. The historical anchors in the ROBINS-I records were retained solely for traceability and were not used as single representative estimates for these reports. Related results require an explicit check of whether the same domain judgments apply, with separate assessment if population, exposure timing, comparator, outcome or analytical method changes. A qualitative sensitivity assessment examined whether interpretation changed across these alternative results. The rules and sensitivity assessment were introduced during revision and do not establish that the original selections were independent of the observed results.

Because different reports may have used the same database or partially overlapping study populations, we assessed potential overlap according to database or cohort name, country or region, study period, eligibility criteria, sample size, population characteristics, participating institutions, and author teams (Hussein et al., 2022). When reports used the same or potentially overlapping population and addressed the same or a closely related research question, one report was selected as representative, while the remaining reports were retained as alternative or supportive evidence rather than treated as independent replications. Related reports evaluating different medications, comparators, or outcomes were presented separately, but their incomplete independence was considered in the overall interpretation. Accordingly, the number of included reports was not interpreted as the number of independent replications, and potentially overlapping reports were not counted cumulatively when judging the consistency or strength of the evidence. Detailed judgments and management decisions are provided in Supplementary Table S8 and Supplementary Figure S6.

### 2.5 Risk-of-bias assessment

Two reviewers (Z.H.L. and J.Y.) independently assessed the risk of bias in the report-level assessment anchor selected from each included report using the seven-domain Risk Of Bias In Non-randomized Studies of Interventions framework described in the 2016 publication (ROBINS-I) (Sterne et al., 2016). Before each assessment, we specified the target medication, comparator, outcome, analytic population, target effect, and key confounders. The domains assessed were bias due to confounding, selection of participants, classification of interventions, deviations from intended interventions, missing data, measurement of outcomes, and selection of the reported result. Domain-level and overall risk of bias were categorized as low, moderate, serious, critical, or no information.

The same seven domain headings were retained across designs, but the design-specific sources of bias differ. For cohorts, relevant issues include alignment of treatment initiation and follow-up, selection based on subsequent treatment persistence, switching, censoring, and missing covariates. For case-control and nested case-control analyses, interpretation additionally requires assessment of the source population, selection of controls, matching of index dates and prior observation time, and ascertainment of prescriptions before the index date. Claims-recorded prescriptions do not automatically eliminate selection or confounding bias. The design-specific considerations summarized in Supplementary Table S9D clarify the interpretation of the existing judgments; they do not reconstruct missing historical signalling-question forms. Imprecision and indirectness from a broader outcome were considered separately from bias in measurement of that outcome.

Overall judgments followed ROBINS-I guidance and were not based on numerical scoring or simple summation of domain-level assessments. Disagreements were resolved through discussion between the two reviewers, with adjudication by a third reviewer (D.P.C.) when consensus could not be reached. The individual pre-consensus assessment forms were no longer available; therefore, agreement before consensus could not be calculated. Risk-of-bias judgments were incorporated directly into the narrative synthesis and interpretation of medication-specific findings; results at serious risk of bias were retained but interpreted cautiously and were not regarded as robust evidence of causal effects. The ROBINS-I assessment anchor and the key risk-of-bias concerns for each report are summarized in Supplementary Table S3. Domain-level judgments and their distribution are presented in Supplementary Table S9A, B. Supplementary Table S9C describes the applicability of the anchor assessment to related results and identifies additional result-specific concerns. Source-based explanations for the domain judgments clarified during revision are provided in Supplementary Table S9E.

### 2.6 Evidence synthesis

All included reports were synthesized narratively and grouped by medication class, comparator, and outcome type. Psoriasis-specific incident psoriasis, psoriasis-specific first-recorded psoriasis, broader psoriasis-related outcomes, and composite psoriasis/psoriatic arthritis outcomes were synthesized as distinct outcome categories. Study-reported incident psoriasis outcomes with unconfirmed psoriasis specificity were explicitly flagged and were not grouped with confirmed psoriasis-specific outcomes. The label “study-reported incident” preserves the original investigators’ terminology and does not independently establish true disease onset. Incident psoriasis and first-recorded psoriasis were not considered synonymous, and broader or composite psoriatic-disease outcomes were not treated as interchangeable with psoriasis-specific outcomes. Findings were interpreted in the context of study design, time-zero specification, effect estimate and precision, risk of bias, and data source. Studies evaluating the same medication against different comparators were summarized separately because they addressed different clinical questions. We did not use vote counting based solely on statistical significance or the direction of effect estimates. We also qualitatively appraised the strength of the main comparison-specific findings, considering study design, risk of bias, consistency within comparable contrasts, precision, outcome specificity, and potential population overlap. These appraisals are presented by medication–comparator–outcome contrast in Supplementary Table S13 and were not formal GRADE ratings.

The suitability of meta-analysis was considered within evidence strata defined by medication, comparator, and outcome type. We considered clinical and methodological comparability and the independence of the available estimates when assessing the suitability of quantitative pooling. Statistical heterogeneity was not quantified because no quantitative synthesis was undertaken. Clinical heterogeneity included differences in the target medication, comparator population, underlying patient population, and psoriasis outcome definition, including whether the outcome represented incident psoriasis, first-recorded psoriasis, a broader psoriasis-related outcome, or a composite psoriasis/psoriatic arthritis outcome. Methodological heterogeneity included differences in study design, time-zero specification, new-user or prevalent-user status, exposure assessment, lag periods, follow-up and censoring rules, treatment switching, and methods used to control confounding. Within most medication–comparator–outcome strata, only one or a small number of mutually independent studies were available, and several reports may have used overlapping database populations. Consequently, statistical heterogeneity could not be meaningfully or reliably estimated within most strata. Even where more than one estimate was available, differences in comparator definition, outcome ascertainment, temporal design, or population independence meant that the estimates did not represent a sufficiently homogeneous target contrast for a clinically interpretable pooled effect. Quantitative pooling was therefore not undertaken. Hazard ratios, odds ratios, and risk ratios were presented as originally reported and were not converted into a common effect measure. Forest plots display study-specific effect estimates and 95% confidence intervals without statistical weights or pooled effects. Funnel plots and statistical tests for publication bias were not performed because no meta-analysis was conducted and each evidence group contained only a small number of independent studies.

## 3 Results

### 3.1 Study selection

The database searches yielded 3,606 records, including 509 from PubMed, 2,057 from Embase, 1,017 from Web of Science Core Collection, and 23 from the Cochrane Library. After removal of 760 duplicate records, 2,846 records underwent title and abstract screening, of which 2,813 were excluded. The remaining 33 full-text reports were assessed for eligibility according to the predefined inclusion and exclusion criteria. Of these, 16 reports were excluded: four were duplicate publications without additional relevant information, seven used ineligible study designs, and five did not report a relevant incident psoriasis outcome. Seventeen reports were therefore included in the initial review.

During manuscript revision, an updated literature search was conducted on 11 September 2026 to identify newly available evidence. This updated search identified 268 additional records from PubMed (n = 112), Embase (n = 68), Web of Science Core Collection (n = 85), and the Cochrane Library (n = 3). After removal of 102 duplicate records, 166 records remained for title and abstract screening, of which 163 were excluded. Three reports underwent full-text assessment; two were already included in the original review, and one newly eligible report was identified. Therefore, the final review included 18 reports (Chen et al., 2025; Chiang et al., 2026; Ma et al., 2024; Lee et al., 2025; Karacabeyli et al., 2026; Hung et al., 2025; Chiu et al., 2024; Chen et al., 2020; Ching et al., 2025; Ma et al., 2022; Tseng, 2023; Huang et al., 2021; Wu et al., 2015; Brauchli et al., 2008; Brauchli et al., 2011; Han et al., 2025; Chodick et al., 2015; Mahajan et al., 2026). The study selection process is shown in Figure 1 and Supplementary Figure S7.

The retained records provide aggregate counts but do not establish whether the 102 removed duplicates included matches to the initial search library. The 166 remaining records should therefore not be interpreted as 166 previously unseen records. The initial full-text exclusions are available only as aggregate categories; a report-by-report exclusion log could not be recovered. These reporting limitations are described in the Supplementary Material.

### 3.2 Characteristics of the included studies

The included reports were published between 2008 and 2026 and were based on real-world healthcare data. They used cohort, case-control, and nested case-control designs, including active-comparator new-user analyses, target trial emulations, and landmark cohort approaches. Data were derived primarily from administrative healthcare databases, insurance claims databases, and electronic health records. Most studies used propensity score matching, propensity score weighting, or multivariable regression to control for confounding. The main characteristics of the included reports are summarized in Table 1, with further details on study design and population characteristics provided in Supplementary Table S11.

**TABLE 1:** Characteristics of the included reports.

Report	Country/region and data source	Study design and study period	Analytic sample size	Medication exposure and comparator	Outcome definition	Outcome category for synthesis
(Chen et al., 2025)	Taiwan; NHIRD	Study-reported time-stratified propensity score–matched active-comparator new-user cohort and target trial emulation; 2006–2015. Alignment of comparator treatment initiation with the start of follow-up was not fully established	DPP-4i: 9,962 vs. sulfonylurea: 39,848; TZD: 1,066 vs. sulfonylurea: 4,264	DPP-4i or TZD vs. sulfonylurea	Study-reported incident psoriasis, defined by at least two visits with ICD-9-CM codes 696.0, 696.1, 696.5, or 696.8 within 365 days after the index date; the definition included psoriatic arthropathy codes	Broader psoriasis-related outcome including PsA codes
(Chiang et al., 2026)	United States; TriNetX US Collaborative Network	Active-comparator new-user target trial emulation with an ITT approach and 1:1 PSM; 2018–2022	169,630 vs. 169,630	GLP-1RA vs. DPP-4i	Study-reported incident psoriasis identified using ICD-10-CM code L40 after a 90-day lag period; exclusion of arthropathic psoriasis codes was not explicitly documented	Study-reported incident psoriasis; restriction to cutaneous psoriasis unconfirmed
(Huang et al., 2021)	Taiwan; LHDB/NHIRD	Retrospective matched cohort study; 1999–2013	1,299 vs. 12,990	α-Glucosidase inhibitor plus metformin vs. metformin monotherapy	Incident psoriatic disease, defined as a composite of psoriasis or psoriatic arthritis	Composite incident psoriatic disease (PsO/PsA)
(Tseng, 2023)	Taiwan; NHIRD	Retrospective landmark cohort with a 12-month exposure-assessment window and IPTW; 1999–2009, with follow-up through 2011	234,585 vs. 125,921	Metformin vs. mixed non-metformin therapy	Incident psoriasis-related diagnosis identified using ICD-9-CM code 696	Broader incident psoriasis-related outcome
(Hung et al., 2025)	Taiwan; LHID within the NHIRD	1:4 matched retrospective cohort study; 2009–2021	15,773 vs. 63,092	DPP-4i vs. non-DPP-4i oral glucose-lowering therapy	Psoriasis, psoriatic arthritis, and composite psoriatic disease reported separately	Psoriasis-specific outcome available; PsA and composite outcomes reported separately
(Chiu et al., 2024)	Taiwan; NHIRD	Propensity score–adjusted retrospective cohort study; 2009–2019	1,312,521 vs. 2,700,856	DPP-4i vs. mixed non-DPP-4i therapy	Broad psoriasis-related outcome including psoriatic arthritis codes	Broader psoriasis-related outcome including PsA codes
(Chen et al., 2020)	Taiwan; NHIRD diabetes subdatabase	Prescription-time distribution matching and 1:1 propensity score–matched cohort; 2009–2013	387,099 vs. 387,099	DPP-4i vs. DPP-4i-naïve mixed therapy	Broad psoriasis-related diagnosis after the index date	Broader psoriasis-related outcome
(Wu et al., 2015)	Taiwan; NHIRD	Nested case-control study within a nationwide diabetes cohort; 1997–2011	9,243 cases and 36,972 controls	Frequent metformin, TZD, or insulin use vs. nonuse of the corresponding medication	First psoriasis- or psoriatic arthritis-related diagnosis, identified by hospitalization or three consecutive specialist diagnoses	First-recorded broader/composite psoriatic-disease outcome
(Ma et al., 2024)	United States; TriNetX electronic health records from 92 healthcare organizations	Propensity score–matched active-comparator treatment-initiator cohort with an ITT approach; 2014–2024	550,195 vs. 550,195	SGLT2i vs. DPP-4i	Study-reported incident psoriasis identified using ICD-10 code L40; exclusion of arthropathic psoriasis codes was not explicitly documented	Study-reported incident psoriasis; psoriasis specificity unconfirmed
(Ma et al., 2022)	Taiwan; NHIRD-MOHW	Matched cohort of persistent medication users with competing-risk analysis; 2007–2018	103,745 vs. 207,490	Persistent SGLT2i use vs. mixed non-SGLT2i therapy	Study-reported incident psoriasis: ICD-9-CM 696.1 or ICD-10 L40; at least three specialist outpatient diagnoses or one inpatient diagnosis. Restriction to cutaneous psoriasis unconfirmed	Study-reported incident psoriasis; cutaneous specificity unconfirmed
(Brauchli et al., 2008)	United Kingdom; GPRD	1:1 matched case-control study; 1994–2005	36,702 cases and 36,702 controls	TZD, metformin, sulfonylurea, or acarbose vs. nonuse of the corresponding medication	First-recorded psoriasis	First-recorded psoriasis; restriction to cutaneous psoriasis unconfirmed
(Ching et al., 2025)	United States; All of Us	Retrospective longitudinal EHR association analysis using multivariable logistic regression; data queried from 2018 to 2023	73,225: 14,178 exposed/59,047 unexposed	Any prior GLP-1RA exposure vs. no GLP-1RA exposure	Newly recorded psoriasis diagnosis after GLP-1RA exposure	First-recorded psoriasis; cutaneous specificity unconfirmed
(Brauchli et al., 2011)	United Kingdom; GPRD	1:1 matched case-control study; 1994–2005	36,702 cases and 36,702 controls	Statin or fibrate use vs. no lipid-lowering medication use	First-recorded psoriasis	First-recorded psoriasis; restriction to cutaneous psoriasis unconfirmed
(Han et al., 2025)	Republic of Korea; NHIS-HEALS	Matched case-control study nested within a health-screening cohort; 2002–2019	8,285 cases and 33,140 controls	Cumulative statin use during the 2 years before the index date vs. nonuse	Broad psoriasis-related outcome including psoriatic arthritis codes	Broader psoriasis-related outcome including PsA codes
(Chodick et al., 2015)	Israel; Maccabi Healthcare Services	Retrospective cohort of new statin users; 1998–2009, with follow-up through 2010	205,820	Different PDC categories, with PDC <20% as the reference	Incident psoriasis recorded by a specialist or during hospitalization	Psoriasis-specific incident psoriasis
(Karacabeyli et al., 2026)	Canada; Population Data BC	Active-comparator new-user cohort with generalized propensity score matching weights; 2014–2022, with follow-up through 2023	GLP-1RA: 49,514 treatment courses; SGLT2i: 101,925; DPP-4i: 77,861	GLP-1RA or SGLT2i vs. DPP-4i	Incident psoriatic disease, defined as a composite of psoriasis or psoriatic arthritis	Composite incident psoriatic disease (PsO/PsA)
(Lee et al., 2025)	United States; TriNetX US Collaborative Network	Active-comparator new-user ITT cohort with 1:1 PSM; initiation during 2015–2022, with observation through 2025	290,770 vs. 290,770	GLP-1RA vs. DPP-4i	Study-reported incident psoriasis from day 90; eMethod: UMLS:ICD10CM:L40 for outcome and baseline exclusion; no explicit L40.5 exclusion	Study-reported incident psoriasis; psoriasis specificity unconfirmed
(Mahajan et al., 2026)	Multinational; TriNetX Global Collaborative Network	Three active-comparator new-user emulated target trials with an ITT approach and 1:1 PSM; 2016–2023, with follow-up through 25 February 2025	DPP-4i vs. GLP-1RA: 118,419 vs. 118,419; DPP-4i vs. SGLT2i: 102,810 vs. 102,810; GLP-1RA vs. SGLT2i: 105,869 vs. 105,869	New initiation of DPP-4i, GLP-1RA, or SGLT2i monotherapy in pairwise active-drug comparisons	Study-reported incident psoriasis identified using ICD-10-CM code L40 after a 6-month landmark. Psoriatic arthritis was reported separately, but mutual exclusivity of the coding definitions was not established	Study-reported incident psoriasis; restriction to cutaneous psoriasis unconfirmed

Outcome definitions retain the ascertainment approaches reported in the original studies. Temporal ascertainment and diagnostic scope are described separately. Study-reported incident and first-recorded outcomes are distinguished; neither label independently establishes biological disease onset. Diagnostic scope is classified as cutaneous psoriasis, a broader psoriasis-related coding definition, a composite psoriasis/psoriatic arthritis outcome, or unconfirmed cutaneous specificity. These diagnostic categories are not treated as interchangeable. Abbreviations: DPP-4i, dipeptidyl peptidase-4, inhibitor; EHR, electronic health record; GLP-1RA, glucagon-like peptide-1, receptor agonist; GPRD, general practice research database; ICD, international classification of diseases; IPTW, inverse probability of treatment weighting; ITT, intention-to-treat; LHDB, longitudinal health insurance database; LHID, longitudinal health insurance database; NHIRD, national health insurance research database; NHIS-HEALS, National Health Insurance Service–Health Screening Cohort; PDC, proportion of days covered; PsA, psoriatic arthritis; PsO, psoriasis; PSM, propensity score matching; SGLT2i, sodium-glucose cotransporter-2, inhibitor; TZD, thiazolidinedione.

Absolute outcome data were not uniformly reported (Supplementary Table S12). For example, Chen WS reported 17 versus 178 events and rates of 188.47 versus 467.00 per 100,000 person-years for DPP-4i versus sulfonylurea, using a broader PsA-inclusive definition (Chen et al., 2025). Hung reported 42 versus 286 cutaneous-psoriasis events and rates of 37.24 versus 63.77 per 100,000 person-years (Hung et al., 2025). These unadjusted rates concern different comparisons and outcome definitions and should not be compared as treatment effects. Case-control reports contributed case and control exposure counts rather than medication-group incidence risks. Ching’s psoriasis-analysis cohort comprised 73,225 patients, including 14,178 exposed and 59,047 unexposed patients, with 182 and 1,419 psoriasis diagnoses, respectively; these counts do not establish equal-duration incidence risks. In Brauchli 2008, the current ≥5-prescription TZD stratum contained 12 cases and 36 controls (Supplementary Table S12), emphasizing sparse exposure rather than providing an incidence denominator.

The medications evaluated included dipeptidyl peptidase-4 inhibitors (DPP-4i), glucagon-like peptide-1 receptor agonists (GLP-1RA), sodium-glucose cotransporter 2 inhibitors (SGLT2i), metformin, thiazolidinediones (TZDs), sulfonylureas, α-glucosidase inhibitors, insulin, and statins. Comparators included other active medications, heterogeneous treatment populations, medication non-users, and groups defined by different exposure durations, intensities, or levels of adherence. The studies differed substantially in comparator selection, time-zero specification, exposure definition, follow-up rules, and psoriasis outcome definition, and some reports may have drawn on the same or partially overlapping database populations. Outcomes were presented according to their reported definitions and coding specificity. Broader psoriasis-related and composite psoriasis/psoriatic arthritis outcomes were distinguished from psoriasis-specific outcomes, and estimates with unconfirmed psoriasis specificity were explicitly flagged. Because these comparator categories represent different clinical contrasts and potentially different underlying patient populations, estimates derived from different comparator frameworks were not interpreted as directly interchangeable. The evidence was therefore synthesized narratively without quantitative pooling. The overall evidence structure is shown in Figure 2, and the primary effect estimates and their roles in the evidence synthesis are provided in Supplementary Table S2.

**FIGURE 2:**
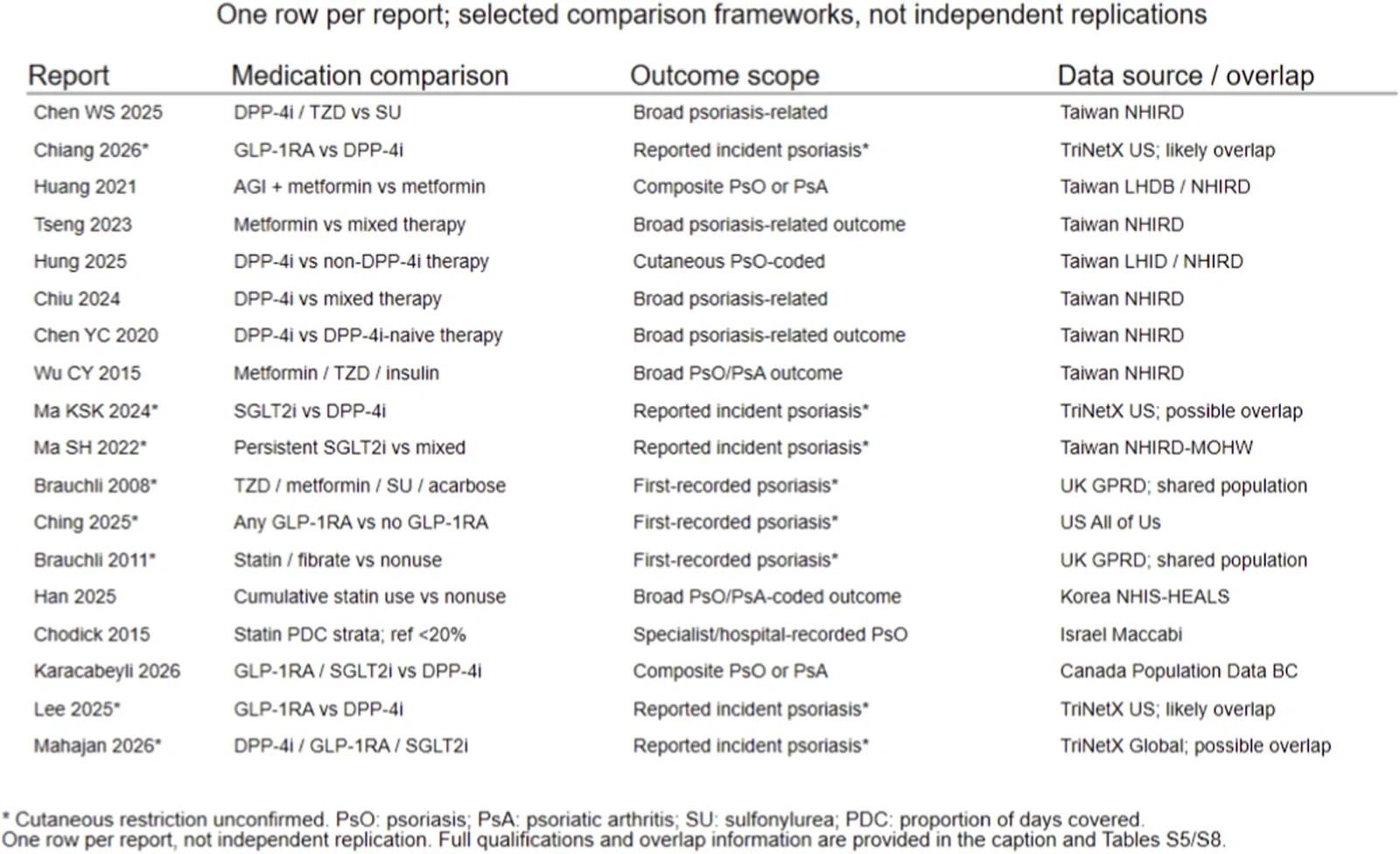
Evidence structure across 18 reports of medication exposure and psoriasis-related outcomes. Each row represents one report and summarizes selected medication comparisons, outcome scope, and data source, including potential population overlap. The comparisons shown are illustrative and do not enumerate every analysis within each report. Reports differ in their outcome definitions, including cutaneous psoriasis, broader psoriasis-related outcomes, and composite outcomes encompassing psoriasis or psoriatic arthritis. Reports using the same database may include overlapping populations and should not be assumed to represent independent replications. Asterisks indicate that restriction of the outcome to cutaneous psoriasis could not be confirmed. Detailed outcome definitions and overlap assessments are provided in Supplementary Table S5, S8. Abbreviations: AGI, alpha-glucosidase inhibitor; DPP-4i, dipeptidyl peptidase-4 inhibitor; GLP-1RA, glucagon-like peptide-1 receptor agonist; PDC, proportion of days covered; PsA, psoriatic arthritis; PsO, psoriasis; SGLT2i, sodium–glucose cotransporter-2 inhibitor; SU, sulfonylurea; TZD, thiazolidinedione.

### 3.3 Glucose-lowering medications

#### 3.3.1 Dipeptidyl peptidase-4 inhibitors

Effect estimates for DPP-4i were generally below 1. In an active-comparator study, Chen W-S et al. compared DPP-4i with sulfonylureas and reported an HR of 0.422 (95% CI 0.273–0.716) for the study-reported psoriasis outcome (Figure 3). The outcome definition explicitly included ICD-9-CM code 696.0 for psoriatic arthropathy and was therefore classified in this review as a broader psoriasis-related outcome including PsA codes. The study flow description specified exclusion of psoriasis diagnosed before T2DM diagnosis, but did not clearly establish exclusion of all psoriasis recorded before medication initiation. The exposed-group index date was the first target-drug prescription, whereas the control-group index date was randomly assigned with reference to the distribution of time from T2DM diagnosis to treatment in the exposed group. Thus, alignment of treatment initiation and follow-up between groups was not fully established by the reported methods. Three additional studies using Taiwanese databases compared DPP-4i users with differently composed non-DPP-4i treatment groups and also reported HRs below 1. However, these supporting reports differed in comparator composition, outcome definitions, and temporal designs and may have included overlapping populations; they were therefore not interpreted as independent replications of a DPP-4i effect (Figure 4). Results for composite outcomes and sensitivity analyses are presented in Supplementary Table S4B.

**FIGURE 3:**
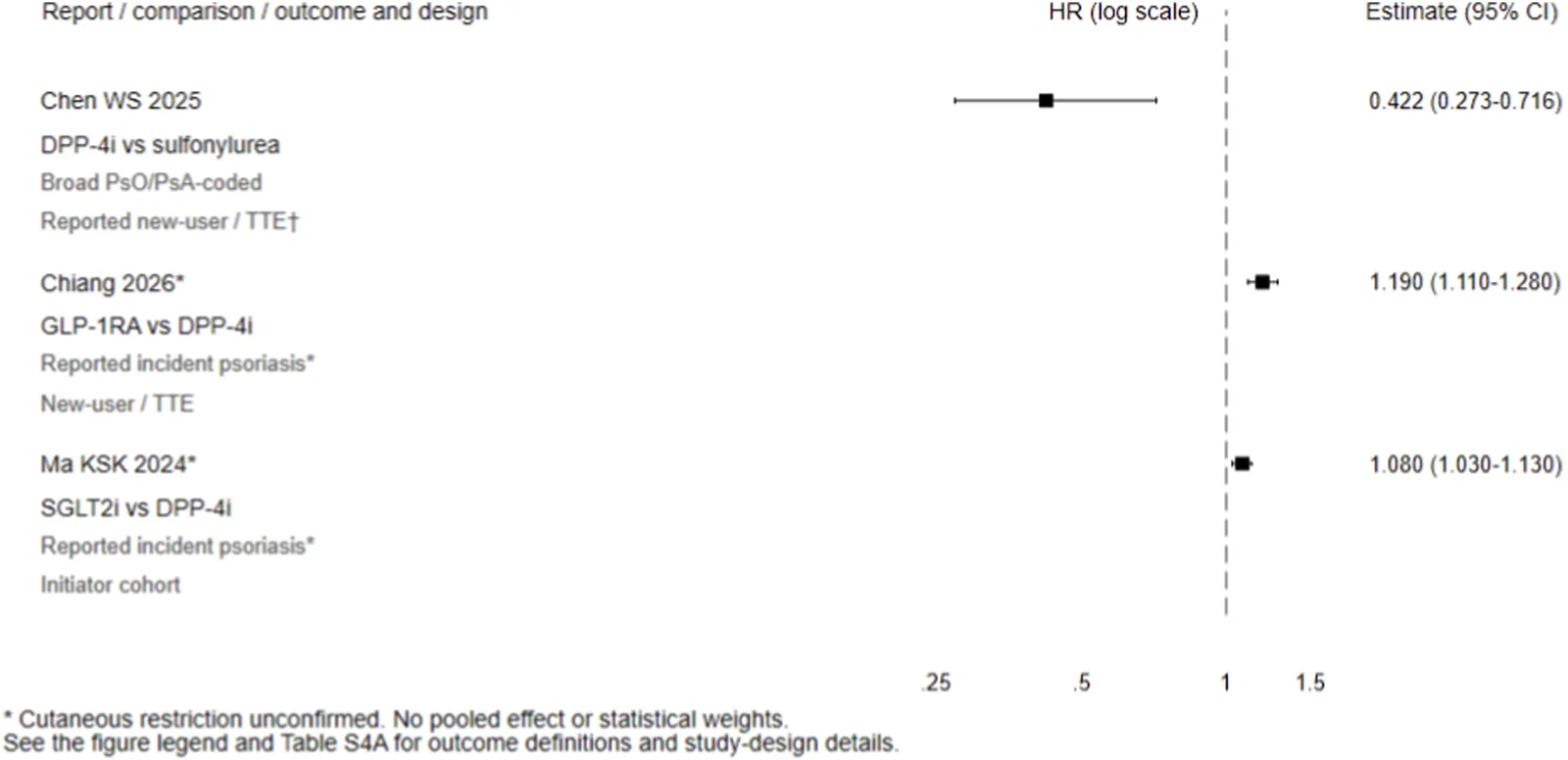
Selected active-comparator estimates for glucose-lowering medications and study-specific psoriasis outcomes. Filled squares indicate reported hazard ratios (HRs), and horizontal lines indicate 95% confidence intervals. The forest axis is logarithmic; the vertical dashed line indicates the null value of 1. The Chen WS estimate concerns a broader psoriasis-related outcome explicitly including psoriatic arthritis codes. Asterisks identify the Chiang and Ma KSK estimates, for which restriction to cutaneous psoriasis could not be confirmed. “Reported incident psoriasis” retains the original study terminology and does not independently establish biological disease onset. †For Chen WS, “Reported new-user/TTE” reflects the design reported by the original investigators. The exposed-group index date was the first target-drug prescription, whereas the comparator-group index date was randomly assigned with reference to the exposed group’s distribution of time from diabetes diagnosis to treatment. Alignment of comparator treatment initiation with the start of follow-up was therefore not fully established. Study designs are abbreviated in the figure; detailed index-date definitions, lag periods, follow-up, and analytical strategies are provided in Supplementary Table S4A. Estimates differ in medication comparison, outcome definition, design, and temporal framework and should not be interpreted as directly interchangeable. Marker sizes do not represent statistical weights, and no pooled estimate was calculated. Potential population overlap further limits evidence independence. DPP-4i, dipeptidyl peptidase-4 inhibitor; GLP-1RA, glucagon-like peptide-1 receptor agonist; SGLT2i, sodium-glucose cotransporter-2 inhibitor; PsO, psoriasis; PsA, psoriatic arthritis; TTE, target trial emulation.

**FIGURE 4:**
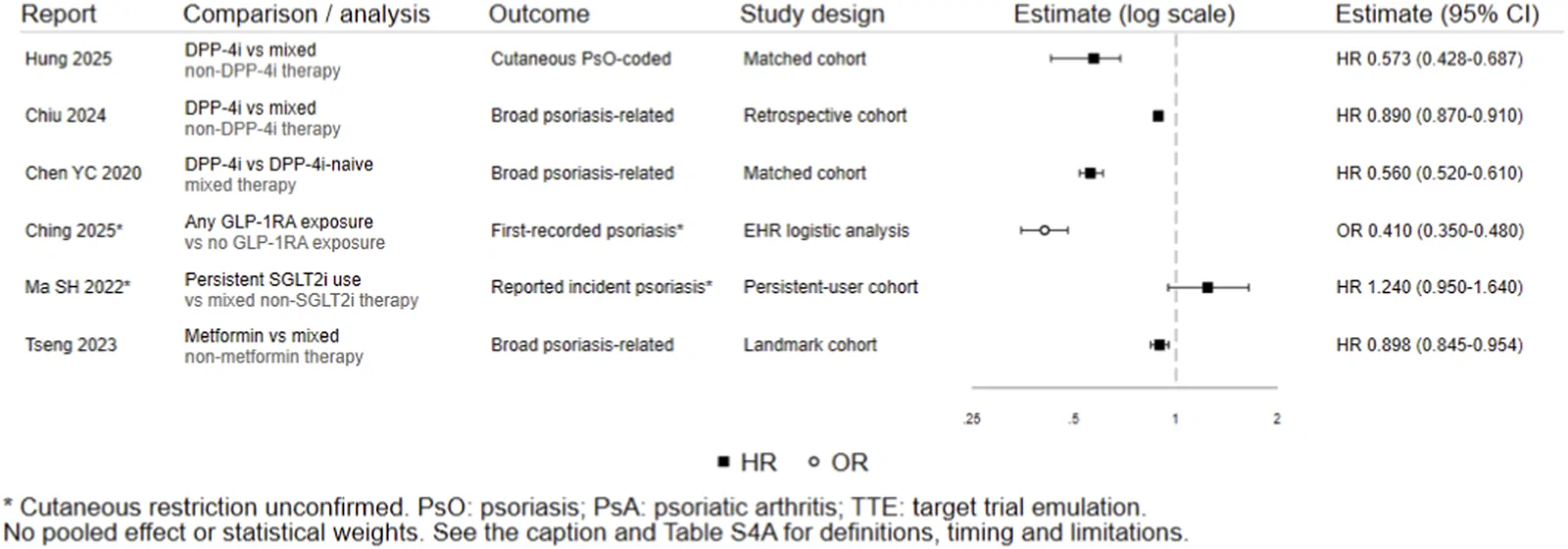
Selected heterogeneous or non-active-comparator estimates for glucose-lowering medications and study-specific psoriasis outcomes. Filled squares indicate hazard ratios (HRs), and open circles indicate odds ratios (ORs); horizontal lines indicate 95% confidence intervals. The numerical forest axis is logarithmic, and the vertical dashed line indicates 1. HRs and ORs are retained as reported without conversion to a common effect measure. Hung is represented by the selected cutaneous psoriasis-coded outcome, with psoriatic arthritis and composite outcomes reported separately. Chiu, Chen YC, and Tseng contributed broader psoriasis-related outcomes. Asterisks identify unconfirmed restriction to cutaneous psoriasis for Ching’s first-recorded outcome and Ma SH’s study-reported incident outcome. A first-recorded diagnosis does not necessarily indicate newly developed disease. Study design is abbreviated in each row; detailed temporal and analytical specifications are provided in Supplementary Table S4A. Differences in comparators, outcome definitions, effect measures, designs, and follow-up preclude direct interchangeability across rows. Marker sizes do not represent statistical weights, and no pooled estimate was calculated. Potentially overlapping reports were not treated as independent replications. EHR, electronic health record; PsO, psoriasis.

### 3.3.2 Glucagon-like peptide-1 receptor agonists

In comparisons with DPP-4i, Chiang et al. reported an HR of 1.190 (95% CI 1.110–1.280) for GLP-1RA and study-reported incident psoriasis (Figure 3) (Chiang et al., 2026). Lee et al. reported an HR of 1.17 (95% CI 1.12–1.22) for the same medication comparison (Lee et al., 2025). These TriNetX reports were considered likely to include overlapping populations and were not treated as independent replications. Mahajan et al. reported the reverse comparison, DPP-4i versus GLP-1RA, with an HR of 0.79 (95% CI 0.70–0.85); the original comparison direction was retained (Mahajan et al., 2026). Separately, Karacabeyli et al. reported an estimate above 1 with a wide confidence interval for GLP-1RA versus DPP-4i using a composite psoriasis/psoriatic arthritis outcome (Karacabeyli et al., 2026). This composite estimate was interpreted separately from the study-reported psoriasis estimates.

For GLP-1RA versus SGLT2i, Chiang et al. (2026) reported an HR of 0.99 (95% CI 0.91–1.07), and Mahajan et al. (2026) reported an HR of 1.02 (95% CI 0.91–1.15). Both point estimates were close to 1, but the confidence intervals did not establish equivalence between treatments. Additional active-comparator estimates are presented in Supplementary Figure S4A.

Nonuser comparisons differed in direction. In the All of Us study, Ching et al. reported an adjusted OR of 0.410 (95% CI 0.350–0.480) for first-recorded psoriasis among patients with any recorded GLP-1RA exposure versus those without recorded exposure (Figure 4) (Ching et al., 2025). An equivalent index date for nonusers was not clearly specified, limiting temporal comparability. By contrast, Lee et al. reported HRs of 1.20 (95% CI 1.09–1.32), 1.27 (95% CI 1.18–1.37), 1.27 (95% CI 1.19–1.36), and 1.59 (95% CI 1.49–1.69) for GLP-1RA use versus nonuse over follow-up windows from 3 months to 2, 4, 6, and 8 years, respectively (Lee et al., 2025). These were related analyses from one report. The 8-year analysis was flagged for a proportional-hazards assumption test with P < 0.05. Differences in exposure definitions, time zero, follow-up, and analytical methods precluded direct comparison with Ching’s estimate.

Restriction of the psoriasis outcomes reported by Chiang, Lee, Mahajan, and Ching to cutaneous disease could not be confirmed. Detailed coding definitions, baseline exclusions, and temporal specifications are provided in Supplementary Table S4A, S5, S10; potential population overlap is summarized in Supplementary Table S8. Overall, GLP-1RA associations varied across comparator frameworks and nonuser analyses, without establishing a fixed medication-class effect.

#### 3.3.3 Sodium-glucose cotransporter 2 inhibitors

Most point estimates for SGLT2i were above 1. Ma KSK et al. compared SGLT2i with DPP-4i and reported an HR of 1.080 (95% CI 1.030–1.130) for study-reported incident psoriasis (Figure 3). The supplementary methods identified the data source as TriNetX electronic health records from 92 US healthcare organizations and specified psoriasis using ICD-10 code L40. For the analysis of the study-defined psoriasis outcome, patients with that outcome recorded before baseline were excluded. The supplementary methods did not specify a fixed-duration lookback window for this outcome exclusion; the reported 12-month baseline window referred to covariate assessment. However, the available coding specification did not establish whether the L40.5 branch for arthropathic psoriasis was excluded; restriction to cutaneous psoriasis therefore remained unconfirmed. A separate study using the same medication comparison reported a composite psoriasis/psoriatic arthritis outcome with an estimate in the same direction but a wide confidence interval. Because the outcome explicitly combined psoriasis and psoriatic arthritis, this estimate was retained as supporting composite-outcome evidence. It was not treated as interchangeable with the study-reported psoriasis estimate from Ma KSK et al., for which psoriasis specificity remained unconfirmed. Ma SH et al. compared persistent SGLT2i users with a heterogeneous non-SGLT2i treatment population and reported an HR of 1.240 (95% CI 0.950–1.640) (Figure 4). The outcome used ICD-9-CM 696.1 or ICD-10 L40 with repeated specialist or inpatient diagnoses; restriction to cutaneous psoriasis remained unconfirmed because exclusion of the arthropathic-psoriasis branch was not specified. Mahajan et al. provided additional active-comparator evidence involving SGLT2i, including DPP-4i versus SGLT2i and GLP-1RA versus SGLT2i comparisons; the latter was close to the null. Subgroup analyses are presented in Supplementary Figure S4C. Overall, SGLT2i estimates were comparator dependent and should not be interpreted as directly equivalent across different treatment contrasts and outcome definitions.

#### 3.3.4 Other glucose-lowering medications

Studies of metformin primarily used heterogeneous treatment populations or medication non-users as comparators. Tseng compared metformin users with a heterogeneous non-metformin treatment population and reported an HR of 0.898 (95% CI 0.845–0.954) for a psoriasis-related outcome; the per-protocol analysis yielded a similar result. Other studies using medication non-users as the reference group also reported estimates below 1, although the effect estimates were modest and were based mainly on non-active or heterogeneous comparator groups.

The active-comparator estimate for TZDs versus sulfonylureas was below 1 but highly imprecise. In comparisons with nonuse, findings varied across reports and exposure strata. Wu et al. reported an OR of 0.89 (95% CI 0.81–0.98) for frequent TZD use. In Brauchli et al. (2008), current use with ≥5 prescriptions yielded an OR of 0.33 (95% CI 0.16–0.66), whereas current use with 1–4 prescriptions and both past-use strata had confidence intervals that included 1. These findings do not establish a consistent association across TZD exposure categories. Evidence for α-glucosidase inhibitors, including combination therapy with metformin and analyses of acarbose, as well as for insulin and sulfonylureas, was derived from single studies or specific exposure strata. Findings were inconsistent, and some estimates were imprecise. The corresponding effect estimates are shown in Supplementary Figures S2, S4C.

### 3.4 Lipid-lowering medications

Evidence on lipid-lowering medications focused mainly on statins. Brauchli et al. compared any statin use with no lipid-lowering medication use and reported an OR of 1.030 (95% CI 0.950–1.130) for first-recorded psoriasis. Estimates stratified by current, recent, or past use and by the number of prescriptions were distributed on both sides of the null and did not show a consistent exposure pattern.

Han et al. conducted a nested case-control analysis within the Korean NHIS-HEALS database and reported adjusted estimates below 1. Statin exposure was classified by cumulative prescription days during the 2 years preceding the index date, which was the first psoriasis diagnosis for cases and the corresponding date for matched controls. Psoriasis diagnoses recorded in 2002–2003 were excluded using a fixed calendar-period washout. The crude and overlap-weighted adjusted estimates differed in direction. Such a reversal can arise after adjustment and is not, by itself, proof of a reporting error. The reported adjusted estimates were retained as supplementary results, with residual confounding and weighting assumptions considered in interpretation. Chodick et al. compared statin adherence categories among patients who initiated statin treatment, using a proportion of days covered (PDC) below 20% as the reference. PDC was calculated over the observed follow-up period rather than within a fixed baseline exposure window. The reported estimates did not show a consistent monotonic association between increasing PDC and psoriasis risk. Detailed findings are presented in Supplementary Figures S3A, S3B. Overall, the available studies did not show a consistent association between statin use and psoriasis risk or a clear gradient according to exposure duration, prescription frequency, or medication adherence. Representative findings, overall directions, and the main interpretive limitations for each medication class are summarized in Table 2.

**TABLE 2:** Summary of the main evidence by medication class.

Medication class	Primary or representative comparison	Representative effect estimate	Other evidence	Main interpretation
DPP-4i	DPP-4i vs. sulfonylurea	HR 0.422 (95% CI 0.273–0.716)	Three Taiwan database reports using differently composed non-DPP-4i treatment groups and broader or differently ascertained psoriasis-related outcomes also reported estimates below 1. Mahajan et al. (2026) additionally reported lower estimates for study-reported incident psoriasis with unconfirmed cutaneous specificity with DPP-4i versus GLP-1RA (HR 0.79, 95% CI 0.70–0.85) and versus SGLT2i (HR 0.81, 95% CI 0.70–0.91)	Reported DPP-4i estimates were directionally lower relative to some comparators. However, these observations do not demonstrate a reduction in psoriasis incidence. Comparator composition, potential population overlap, differences in outcome ascertainment and temporal design, and residual confounding preclude a stable class-level or causal interpretation. The representative DPP-4i versus sulfonylurea estimate concerned a broader psoriasis-related outcome including PsA codes and was not specific to cutaneous psoriasis
GLP-1RA	GLP-1RA vs. DPP-4i	HR 1.190 (95% CI 1.110–1.280)	Lee reported an estimate in the same direction for the GLP-1RA–DPP-4i comparison, but possible TriNetX overlap limits independence. Mahajan reported DPP-4i versus GLP-1RA HR 0.79 (95% CI 0.70–0.85). For GLP-1RA versus SGLT2i, Chiang reported HR 0.99 (95% CI 0.91–1.07) and Mahajan reported HR 1.02 (95% CI 0.91–1.15). Restriction of these coded psoriasis outcomes to cutaneous psoriasis was unconfirmed. The Canadian report provided separate composite PsO/PsA evidence with a wide confidence interval. Ching reported an OR of 0.410 (95% CI 0.350–0.480) for GLP-1RA exposure versus nonuse. Lee’s supplementary nonuser comparisons yielded HRs of 1.20 (95% CI 1.09–1.32), 1.27 (1.18–1.37), 1.27 (1.19–1.36), and 1.59 (1.49–1.69) for follow-up windows from 3 months to 2, 4, 6, and 8 years, respectively. The 8-year analysis was flagged for a proportional-hazards assumption test with P < 0.05. These analyses differed from Ching’s comparison in their temporal and analytical frameworks and were not treated as interchangeable estimates	Findings varied by comparator, outcome definition, and temporal design. The nonuser comparisons are not directly interchangeable, and near-null GLP-1RA–SGLT2i estimates do not establish equivalence. Potentially overlapping reports are not confirmed independent replications. The evidence does not establish a fixed GLP-1RA class effect
SGLT2i	SGLT2i vs. DPP-4i	HR 1.080 (95% CI 1.030–1.130)	Persistent SGLT2i use versus mixed non-SGLT2i therapy yielded an HR of 1.240 (95% CI 0.950–1.640) for study-reported incident psoriasis with unconfirmed cutaneous specificity. Separately, a Canadian study using a composite psoriasis/psoriatic arthritis outcome reported an estimate in the same direction but with limited precision. Mahajan et al. (2026) reported DPP-4i versus SGLT2i HR 0.81 (95% CI 0.70–0.91) and GLP-1RA versus SGLT2i HR 1.02 (95% CI 0.91–1.15) for study-reported incident psoriasis with unconfirmed cutaneous specificity	The representative SGLT2i versus DPP-4i estimate from Ma KSK et al. concerned study-reported incident psoriasis, with psoriasis specificity unconfirmed. The available estimates were comparator dependent. Study-reported psoriasis with unconfirmed cutaneous specificity and composite PsO/PsA outcomes were interpreted separately. The number of mutually independent studies was small, and differences in comparator, outcome ascertainment, and study design preclude a stable class-level interpretation. Residual confounding related to cardiorenal risk, obesity, diabetes characteristics, and treatment selection remains a major concern. The modestly elevated estimates should not be interpreted as evidence that SGLT2i increase psoriasis incidence
Metformin	Metformin vs. mixed non-metformin therapy	HR 0.898 (95% CI 0.845–0.954)	Estimates from nonuser comparisons and other exposure definitions were also below 1, although these studies used broader or differently ascertained psoriasis-related outcomes	Lower estimates were observed in some studies, but the evidence was based primarily on mixed or non-active comparators and included broader or first-recorded psoriasis-related outcomes. These findings do not establish a lower risk of psoriasis-specific incident disease and remain susceptible to residual confounding
TZD	TZD vs. sulfonylurea	HR 0.347 (95% CI 0.039–3.049)	Nonuser comparisons differed across reports and exposure strata. Wu reported an OR of 0.89 (95% CI 0.81–0.98) for frequent TZD use. In Brauchli 2008, the markedly lower estimate was confined to current use with ≥5 prescriptions; the confidence intervals for current use with 1–4 prescriptions and both past-use strata included 1. These findings do not establish a consistent association across TZD exposure categories	The active-comparator estimate concerned a broader psoriasis-related outcome including PsA codes. It was based on very few outcome events and was severely imprecise. The available evidence is insufficient to establish a stable association or an association specific to cutaneous psoriasis
Other glucose-lowering medications	Combination-treatment, medication-nonuser, or exposure-intensity comparisons	No single estimate was considered sufficiently representative of this evidence group	The estimate for an α-glucosidase inhibitor added to metformin was elevated but imprecise and was based on a composite psoriasis/psoriatic arthritis outcome. Evidence for insulin, sulfonylureas, and acarbose was derived mainly from single studies or specific exposure strata using heterogeneous outcome definitions	The available evidence was sparse, methodologically heterogeneous, and based on heterogeneous psoriasis-related outcomes. No consistent psoriasis-specific incident pattern has been established
Statins	Any statin use vs. no lipid-lowering medication use	OR 1.030 (95% CI 0.950–1.130)	Cumulative-use analyses based on a broader psoriasis-related outcome reported estimates below 1, but crude and adjusted estimates differed in direction, which does not by itself establish a reporting error. Adherence analyses among statin initiators evaluated incident psoriasis recorded by a specialist or during hospitalization and showed no stable gradient	The representative estimate for first-recorded psoriasis was close to the null. Supporting studies using broader or incident psoriasis outcomes showed no stable pattern according to exposure duration, prescription frequency, or medication adherence. Current evidence does not establish a consistent association across these distinct outcome definitions
Other lipid-lowering medications	Fibrate use vs. no lipid-lowering medication use	No estimate was considered suitable for primary interpretation	The number of exposed cases was small, and the available estimates were imprecise	Evidence for non-statin lipid-lowering medications was sparse and imprecise, and no stable association could be inferred

The “Main interpretation” column summarizes the direction and principal limitations of the available observational evidence and does not represent a formal certainty-of-evidence rating. Incident psoriasis, first-recorded psoriasis, broader psoriasis-related outcomes, and composite psoriasis/psoriatic arthritis outcomes were treated as distinct outcome categories and were not considered interchangeable. First-recorded psoriasis was not assumed to represent true disease onset. Effect estimates were retained as reported in the original studies and were not transformed or quantitatively pooled. Potentially overlapping reports were not treated as fully independent replications. The three pairwise psoriasis estimates reported by Mahajan et al. (2026) arose from a single report and were not treated as three independent replications.

### 3.5 Sensitivity assessment

In a revision-stage qualitative sensitivity assessment, Chodick’s sex-specific PDC results did not show progressively lower hazard ratios with increasing adherence. At PDC ≥80%, the adjusted HR was 0.88 (95% CI 0.79–0.98) in women and 1.00 (0.90–1.11) in men; intermediate categories had lower estimates. These differences do not establish a sex interaction. In Brauchli 2008, current TZD use yielded ORs of 1.01 (0.34–3.01) for 1–4 prescriptions and 0.33 (0.16–0.66) for ≥5 prescriptions, while past-use estimates were 1.21 (0.41–3.60) and 0.93 (0.38–2.31), respectively. Thus, the lower estimate was specific to one exposure stratum and should not be generalized to all TZD use. In Han, both duration strata yielded adjusted estimates below 1 (1–365 days: OR 0.70, 95% CI 0.66–0.74; >365 days: OR 0.77, 95% CI 0.73–0.82), although this did not establish a progressively stronger association with longer exposure. Considering the alternative strata therefore weakens a generalized interpretation of the Brauchli TZD result, while leaving the cautious interpretation of statin evidence unchanged.

### 3.6 Overlap in data sources and risk of bias

Several groups of reports may have used the same or partially overlapping study populations, particularly those based on TriNetX networks, the General Practice Research Database in the United Kingdom, and the National Health Insurance Research Database in Taiwan. Chiang et al. and Lee et al. both used the TriNetX US Collaborative Network and evaluated GLP-1RA versus DPP-4i in adults with T2DM. Their study periods overlapped (2018–2022 and 2015–2022, respectively), supporting a judgment of likely population overlap; participant-level overlap could not be verified. The GLP-1RA–DPP-4i estimates from Chiang et al. and Lee et al. were interpreted together within this likely overlapping evidence cluster. Chiang et al. is displayed in Figure 3, and Lee et al. in Supplementary Figure S4A; this presentation does not establish methodological superiority of one report over the other. Their reported HRs were 1.19 (95% CI 1.11–1.28) and 1.17 (95% CI 1.12–1.22), respectively. Considering either estimate leaves the descriptive direction of this comparison unchanged, but their agreement was not treated as independent replication. Mahajan et al. also used TriNetX, but through the broader Global Collaborative Network and with different eligibility and temporal specifications; possible partial population overlap with the Chiang and Lee reports could not be excluded, and the study was therefore retained as supporting rather than independent confirmatory evidence. The 2008 and 2011 reports by Brauchli et al. used the same underlying source population of cases and controls from the General Practice Research Database but evaluated different medication classes; both reports were therefore retained. The supplementary methods of Ma KSK et al. also identified TriNetX as the data source, comprising 92 US healthcare organizations. Possible participant overlap with other TriNetX reports could not be excluded, although the extent of overlap could not be established from the available report-level information. Its SGLT2i–DPP-4i estimate was retained for that specific comparison but was not assumed to provide independent replication of findings from other TriNetX reports. Several studies using Taiwan’s National Health Insurance Research Database may also have included partially overlapping populations, although the extent of overlap could not be determined from the available reports. Detailed judgments are provided in Supplementary Table S8 and Supplementary Figure S6. Accordingly, the 18 included reports should not be interpreted as 18 independent study populations. The exact number of independent populations could not be determined from the available report-level information. We therefore describe the shared data sources and the evidence supporting potential overlap rather than provide an unverified numerical count of independent populations. Within potentially overlapping evidence clusters, representative estimates were prioritized, whereas related reports were retained as alternative or supporting evidence and were not treated as independent confirmation of an association. Accordingly, overlap was classified as confirmed for the two Brauchli reports, likely for Chiang and Lee, and possible for the other identified TriNetX and Taiwan database clusters. These classifications describe the evidence for overlap rather than quantify the number of shared participants.

Eighteen report-level ROBINS-I assessment anchors, one from each included report, were assessed. Five estimates (27.8%) were judged to have a moderate overall risk of bias and 13 (72.2%) a serious overall risk of bias; none was judged to have a low overall risk of bias. Thus, no medication-specific finding was supported by evidence judged to be at low risk of bias. Confounding was the most common source of bias, particularly in studies using non-active or heterogeneous comparators and providing insufficient control for treatment indication and disease severity. Other potential sources of bias included participant selection related to persistent-treatment requirements or landmark eligibility and differences in time-zero specification between treatment groups. Reliance on administrative diagnoses also raised concerns about outcome misclassification and differences in diagnostic recording. Separately, broad psoriasis-related definitions and composite psoriasis/psoriatic arthritis outcomes limited the directness of the evidence for cutaneous psoriasis. Neither administrative coding nor a composite outcome was, by itself, sufficient to justify a serious risk-of-bias judgment. These risk-of-bias concerns were considered directly when interpreting the direction and apparent magnitude of the reported associations. Domain-level and overall judgments are presented in Figure 5, Supplementary Figure S5, and Supplementary Tables S9A-C.

**FIGURE 5:**
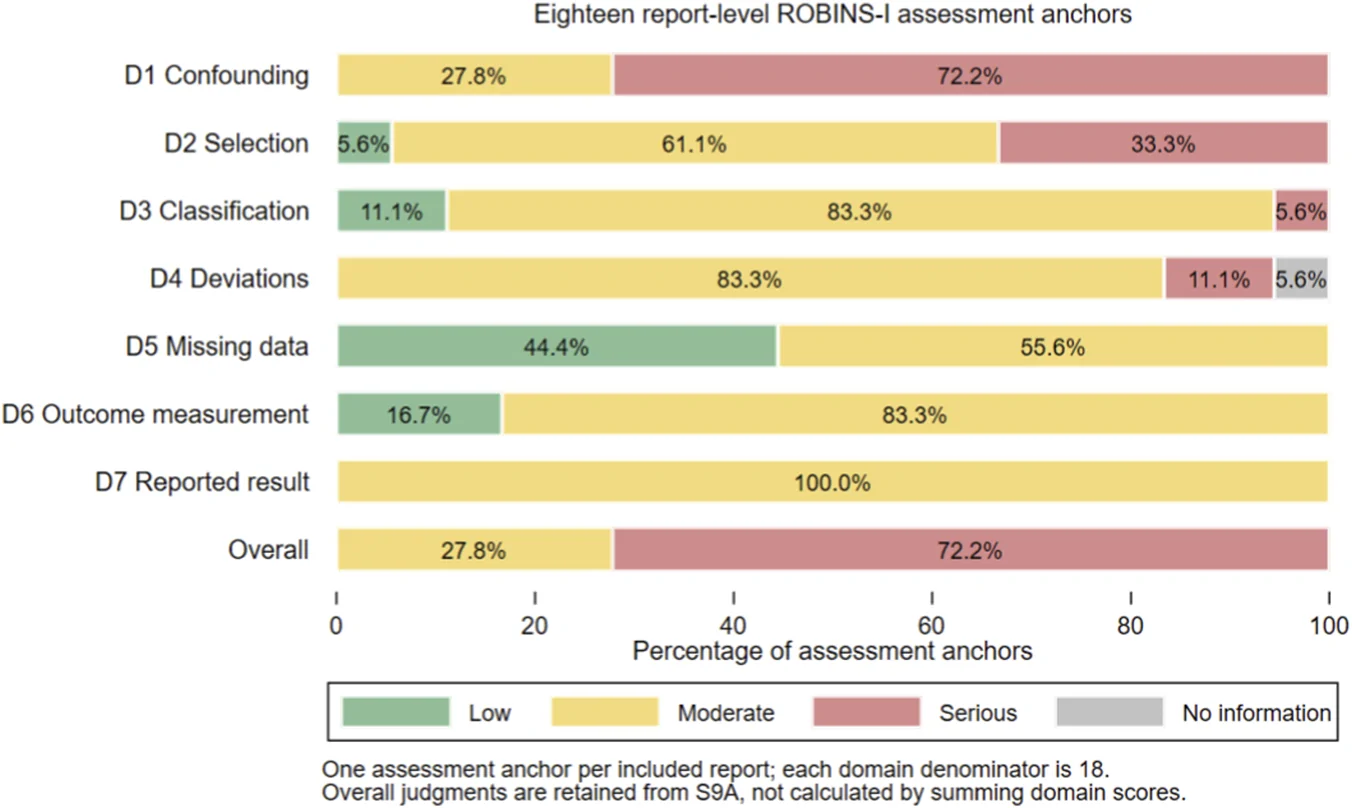
Distribution of ROBINS-I risk-of-bias judgments for report-level assessment anchors. One assessment anchor was retained for each of the 18 included reports; these reports do not necessarily represent 18 independent study populations. Stacked bars show the percentages of anchors assigned low, moderate, serious, or no-information judgments in each domain and overall. The denominator is 18 for every bar. Judgments were taken from Supplementary Table S9A, and their distributions were checked against Supplementary Table S9B. Overall judgments were retained from the report-level assessments and were not calculated by summing domain scores: five anchors (27.8%) were judged moderate and 13 (72.2%) serious. No anchor received a critical judgment in any domain or overall; this absent category is not displayed. Percentages are rounded to one decimal place and may not sum to exactly 100.0%. D1, bias due to confounding; D2, bias in selection of participants into the study; D3, bias in classification of interventions; D4, bias due to deviations from intended interventions; D5, bias due to missing data; D6, bias in measurement of outcomes; D7, bias in selection of the reported result.

## 4 Discussion

### 4.1 Main findings

This systematic review included 18 longitudinal observational reports. Associations varied according to medication, comparator, outcome definition, and study design. Dipeptidyl peptidase-4 inhibitor (DPP-4i) estimates were generally below 1, findings for glucagon-like peptide-1 receptor agonists (GLP-1RA) and sodium-glucose cotransporter-2 inhibitors (SGLT2i) varied across comparisons, and statin studies did not show a consistent pattern across use and adherence contrasts. Of the 18 report-level ROBINS-I assessment anchors, 13 (72.2%) were judged at serious risk of bias and five (27.8%) at moderate risk; none was judged at low risk. These limitations, together with uncertainty about evidence independence, preclude a stable interpretation of the findings as medication-class effects.

Psoriasis involves immune-inflammatory pathways, including IL-23/Th17 signaling, and is associated with cardiometabolic comorbidities (Armstrong and Read, 2020; Griffiths et al., 2021). Whether modulation of these pathways by glucose-lowering or lipid-lowering medications contributes to the observed epidemiological associations remains uncertain. Most included epidemiological analyses did not directly evaluate these pathways. Hung et al. additionally reported *in vitro* experiments in human skin cells (Hung et al., 2025). These experiments provide biological context but do not establish that the same mechanisms mediated the associations observed in the cohort.

Reported DPP-4i estimates were below 1 in the comparison with sulfonylureas (Chen et al., 2025) and in comparisons with heterogeneous non-DPP-4i treatment groups (Hung et al., 2025; Chiu et al., 2024; Chen et al., 2020). Mahajan et al. also reported estimates below 1 for DPP-4i versus GLP-1RA and SGLT2i (Mahajan et al., 2026). These comparisons address different clinical questions and should not be interpreted as interchangeable estimates of a single treatment effect. Several Taiwan reports may also have included overlapping populations, limiting their interpretation as independent replications. DPP-4/CD26 participates in immune regulation, including T-cell activation and effector function (Chitadze et al., 2021), but the available epidemiological evidence does not establish that this pathway explains the observed associations. Confirmation in independent populations with clinically comparable treatment groups is therefore needed.

GLP-1RA findings varied across comparator frameworks. Estimates were above 1 in comparisons with DPP-4i (Chiang et al., 2026; Lee et al., 2025), whereas the GLP-1RA versus SGLT2i estimates reported by Chiang and Mahajan were close to 1 (Chiang et al., 2026; Mahajan et al., 2026). Estimates also differed in direction across nonuser comparisons (Lee et al., 2025; Ching et al., 2025). Differences in obesity, diabetes severity, previous treatment, cardiorenal disease, and healthcare contact may contribute to these patterns despite statistical adjustment. Nonuser comparisons also differed in exposure definitions and follow-up and should not be interpreted as estimates of the same treatment effect. Previous reviews have discussed potential anti-inflammatory effects of GLP-1RA (Karacabeyli and Lacaille, 2024) and their effects on psoriasis severity in patients with established disease (Ku and Chang, 2024). These questions differ from whether GLP-1RA use is associated with incident or first-recorded psoriasis. Such evidence does not resolve the comparator and temporal-design differences among the observational studies included here.

SGLT2i estimates also depended on the comparator and treatment definition. Ma KSK et al. reported a modestly elevated estimate for SGLT2i versus DPP-4i (Ma et al., 2024), whereas Chiang and Mahajan reported estimates close to 1 for GLP-1RA versus SGLT2i (Chiang et al., 2026; Mahajan et al., 2026). The latter comparisons retained GLP-1RA as the exposure group and should not be read as SGLT2i–nonuse comparisons. Persistent-user analyses require particular attention to eligibility and follow-up. In Ma SH et al., follow-up began after the qualifying 3-month exposure period (Ma et al., 2022); that period should not be assumed to have contributed exposed follow-up time. Selection of persistent users and differences between treated patients and the never-user comparator remain relevant concerns.

Metformin was associated with lower estimates in some analyses, although the comparisons differed in exposure definition, treatment context, and outcome scope (Tseng, 2023; Wu et al., 2015). Findings for thiazolidinediones (TZDs) also varied across exposure strata. In Brauchli et al. (2008), current TZD use yielded ORs of 1.01 (95% CI 0.34–3.01) for 1–4 prescriptions and 0.33 (0.16–0.66) for ≥5 prescriptions; the corresponding past-use estimates were 1.21 (0.41–3.60) and 0.93 (0.38–2.31) (Brauchli et al., 2008). The markedly lower point estimate was confined to the current-use ≥5-prescription stratum, while the other strata were imprecise and did not establish a consistent exposure pattern. Anti-inflammatory mechanisms have been discussed for metformin (Feng et al., 2023), while metabolic and anti-inflammatory effects of pioglitazone have been reviewed primarily in a cardiovascular context (Nesti et al., 2021). These findings provide background plausibility but do not establish prevention of cutaneous psoriasis or a shared preventive effect across all TZDs. Evidence for other medication classes remained limited and should be interpreted within the specific reported comparison.

Statin-use and statin-adherence analyses addressed different questions. Brauchli et al. (2011) evaluated first-recorded psoriasis, with restriction to cutaneous psoriasis unconfirmed (Brauchli et al., 2011). Han et al. used a broader psoriasis/psoriatic arthritis-coded outcome and reported adjusted ORs below 1 for both 1–365 days and >365 days of statin use, without a progressively stronger association with longer exposure (Han et al., 2025). Chodick et al. compared sex-specific proportion-of-days-covered (PDC) categories among statin initiators rather than initiation against nonuse (Chodick et al., 2015). The estimates did not form a monotonic adherence gradient, and differences between sex-specific estimates did not establish a sex interaction. Findings concerning first diagnosis should also be distinguished from research on statin effects on severity among patients with established psoriasis (Socha et al., 2020). Variation across these contrasts establishes neither a consistent preventive effect nor the absence of an association.

### 4.2 Influence of study design on evidence interpretation

Comparator choice determines the clinical question addressed by each estimate. Nonusers and heterogeneous treatment groups may differ in treatment indication, disease stage, and healthcare contact. Active-comparator new-user designs can improve comparability, but validity still depends on appropriate alignment of eligibility and follow-up and adequate confounding control. Propensity-score methods cannot eliminate confounding from unmeasured or poorly measured factors, and design or analytical errors can introduce additional bias (Schneeweiss and Patorno, 2021; Bykov et al., 2022).

Temporal ascertainment and diagnostic scope impose separate limits. A first-recorded diagnosis may represent previously undocumented disease, and exclusion of prior codes does not establish biological onset. Cutaneous psoriasis, broader coding definitions, and composite psoriasis/psoriatic arthritis outcomes address different endpoints. Outcomes with unconfirmed cutaneous specificity were therefore distinguished from confirmed cutaneous outcomes in this review.

Potential population overlap further limits evidence independence. Reports using the same healthcare database may include some of the same participants even when their treatment comparisons or eligibility criteria differ. Supplementary Table S8 describes the related TriNetX, General Practice Research Database, and Taiwan database reports included in this review. For example, the GLP-1RA–SGLT2i estimates from Chiang and Mahajan were both close to 1, but possible overlap and differences in eligibility and follow-up limit their interpretation as independent replication. Within the medication–comparator–outcome groups, insufficient comparability and uncertain independence supported retaining study-specific results without quantitative pooling. Structured tabulation and visual presentation were used to make these differences explicit, with reference to SWiM and Cochrane guidance.

### 4.3 Strengths and limitations

A strength of this review was its structured organization of evidence by medication comparison, temporal design, outcome definition, and data source. Treatment-initiation, nonuser, treatment-strategy, and exposure-intensity comparisons were considered separately. Event counts and person-time, where available, provide additional context in Supplementary Table S12.

The evidence remains limited by residual confounding, heterogeneous exposure and outcome definitions, and uncertainty about population independence. Routine healthcare records may incompletely capture prior disease and relevant clinical characteristics, while sparse events limit several estimates. These concerns reduce confidence even when confidence intervals are narrow. Supplementary Table S13 provides comparison-specific interpretation without constituting formal GRADE ratings. In addition, the report-level ROBINS-I assessment-anchor judgments should not be assumed to apply automatically to every secondary comparison or exposure stratum within the same report.

At the review level, retrospective registration limited verification of which decisions had been specified before the review was conducted. Contemporaneous reasons for the original selection of individual assessment anchors were unavailable, limiting auditability. The report-level full-text exclusion log, the precise scope of update deduplication, and detailed database-interface date-filter expressions could not be recovered. These gaps limit the reproducibility and auditability of the review process. Revision-stage changes and clarifications are disclosed in Supplementary Table S14.

### 4.4 Clinical implications and future research

Glucose-lowering medication selection should remain guided by established indications and individual clinical characteristics, including cardiovascular and kidney disease (Davies et al., 2022; de Boer et al., 2022). For patients with psoriasis, statin decisions should be based on cardiovascular risk and lipid-management indications (Gonzalez-Cantero et al., 2023). The associations synthesized in this review do not provide a sufficient basis for prescribing or avoiding these medications solely to prevent psoriasis.

Future studies should use prospectively specified active-comparator new-user designs with clinically appropriate comparators (Schneeweiss and Patorno, 2021). Eligibility, treatment strategies, time zero, and the start of follow-up should be explicitly aligned using target-trial principles where appropriate (Hernán et al., 2022; Matthews et al., 2022). Medication washout, outcome lags, switching, discontinuation, and censoring should be clearly specified and reported in sufficient detail to permit replication (Wang et al., 2021). Psoriasis and psoriatic arthritis should be reported separately using validated algorithms. Event counts, absolute rates, and risk differences should accompany relative estimates, and findings should be replicated in independent data sources. Reports using the same database should provide sufficient information on study periods, participating healthcare settings, eligibility criteria, and analytic populations to permit assessment of potential participant overlap.

## 5 Conclusion

Current observational evidence does not establish causal effects of glucose- or lipid-lowering medications on incident or first-recorded psoriasis. Findings remain specific to the medication comparison and outcome definition and are limited by substantial risk of bias and uncertainty about evidence independence. They do not support selecting or avoiding these medications solely to prevent psoriasis or changing standard diabetes or cardiovascular treatment on the basis of the reported associations.

## Data Availability

All data analyzed in this systematic review were obtained from previously published studies. The data supporting the findings of this review are included in the article and its Supplementary Material. Further inquiries can be directed to the corresponding author.
