# Supplementary material for: Associations of glucose- and lipid-lowering medications with incident or first-recorded psoriasis: a systematic review of real-world observational studies
Source: Front Pharmacol. 2026 Oct 14;17:1942307. doi: 10.3389/fphar.2026.1942307 (PMC13713673; doi:10.3389/fphar.2026.1942307)
Supplement: Supplementary file 1 [file Supplementaryfile1.docx]

contents

[1	Supplementary Figures and Tables	4](#_Toc240412920)

[1.1	Supplementary Tables	4](#_Toc240412921)

[1.1.1	Supplementary Table S1. Detailed search strategies for all electronic databases	4](#_Toc240412922)

[1.1.2	Supplementary Table S2. Key effect estimates for glucose-lowering and lipid-lowering medications and their roles in the evidence framework	9](#_Toc240412923)

[1.1.3	Supplementary Table S3. Overall ROBINS-I judgments for report-level assessment anchors and key result-specific limitations	14](#_Toc240412924)

[1.1.4	Supplementary Table S4A. report design, time zero, and follow-up strategies	18](#_Toc240412925)

[1.1.5	Supplementary Table S4B. Confounding control and statistical methods	20](#_Toc240412926)

[1.1.6	Supplementary Table S5. Exposure, comparator, and outcome definitions of the included reports	23](#_Toc240412927)

[1.1.7	Supplementary Table S6A. Selected report-specific effect estimates used in the evidence synthesis	26](#_Toc240412928)

[1.1.8	Supplementary Table S6B. Alternative, sensitivity, secondary-outcome, exposure-timing, adherence, and subgroup estimates	28](#_Toc240412929)

[1.1.9	Supplementary Table S7. Selection and interpretation of estimates by medication class	31](#_Toc240412930)

[1.1.10	Supplementary Table S8. Potential overlap among databases and report populations and its handling in the evidence synthesis	33](#_Toc240412931)

[1.1.11	Supplementary Table S9A. ROBINS-I assessments for the report-level assessment anchor retained from each included report	35](#_Toc240412932)

[1.1.12	Supplementary Table S9B. Distribution of ROBINS-I judgments across domains	38](#_Toc240412933)

[1.1.13	Supplementary Table S9C. Applicability of ROBINS-I assessment-anchor judgments to related estimates and result-specific concerns	39](#_Toc240412934)

[1.1.14	Supplementary Table S9D. Design-specific considerations for interpreting the ROBINS-I judgments	41](#_Toc240412935)

[1.1.15	Supplementary Table S9E. Source-based clarification of selected ROBINS-I concerns	42](#_Toc240412936)

[1.1.16	Supplementary Table S10. Reporting discrepancies, unavailable details, and their handling in this review	45](#_Toc240412937)

[1.1.17	Supplementary Table S11. Characteristics of reports evaluating medications and incident or first-recorded psoriasis and broader psoriatic outcomes	48](#_Toc240412938)

[1.1.18	Supplementary Table S12. Events and absolute outcome measures	51](#_Toc240412939)

[1.1.19	Supplementary Table S13. Comparison-specific limitations and interpretation of the included evidence	54](#_Toc240412940)

[1.1.20	Supplementary Table S14. Changes and clarifications relative to the registered protocol and initial manuscript	56](#_Toc240412941)

[1.2	Supplementary Figures	58](#_Toc240412942)

[1.2.1	Supplementary Figure S1. Drug-by-report coverage matrix across medication classes.	58](#_Toc240412943)

[1.2.2	Supplementary Figure S2. Drug-nonuser and exposure-intensity comparisons for glucose-lowering medications	59](#_Toc240412944)

[1.2.3	Supplementary Figure S3. Statin exposure timing, cumulative use, and adherence in relation to psoriasis risk	60](#_Toc240412945)

[1.2.4	Supplementary Figure S4. Additional, sensitivity, and exploratory effect estimates across medication comparisons and outcome definitions.	60](#_Toc240412946)

[1.2.5	Supplementary Figure S5. ROBINS-I traffic-light plot for report-level assessment anchors.	62](#_Toc240412947)

[1.2.6	Supplementary Figure S6. Database sources and potential population overlap among the included reports.	63](#_Toc240412948)

[1.3	Updated literature search	63](#_Toc240412949)

[1.3.1	Supplementary Figure S7. Flow diagram of the updated literature search and study selection conducted during manuscript revision.	64](#_Toc240412950)

# 1 Supplementary Figures and Tables

## 1.1 Supplementary Tables

### 2.1.1 Supplementary Table S1. Detailed search strategies for all electronic databases

| **Database** | **Search query** | **Results (hits)** |
| --- | --- | --- |
| **PubMed** | ("Psoriasis"[Mesh] OR psoriasis[Title/Abstract] OR psoriases[Title/Abstract] OR psoriasiform[Title/Abstract] OR "Pustulosis of Palms and Soles"[Title/Abstract] OR "Pustulosis Palmaris et Plantaris"[Title/Abstract] OR "Palmoplantaris Pustulosis"[Title/Abstract] OR "Palmoplantar Pustulosis"[Title/Abstract] OR "Pustular Psoriasis of Palms and Soles"[Title/Abstract]) AND (("Hypoglycemic Agents"[Mesh] OR "Insulin"[Mesh] OR "Metformin"[Mesh] OR "Sulfonylurea Compounds"[Mesh] OR "Thiazolidinediones"[Mesh] OR "Dipeptidyl-Peptidase IV Inhibitors"[Mesh] OR "Glucagon-Like Peptide-1 Receptor Agonists"[Mesh] OR "Sodium-Glucose Transporter 2 Inhibitors"[Mesh] OR "Glycoside Hydrolase Inhibitors"[Mesh] OR "Insulin Secretagogues"[Mesh] OR "Amylin Receptor Agonists"[Mesh] OR antidiabet[Title/Abstract] OR hypoglycemi[Title/Abstract] OR hypoglycaemi[Title/Abstract] OR antihyperglycemi[Title/Abstract] OR antihyperglycaemi[Title/Abstract] OR "glucose-lowering"[Title/Abstract] OR "glucose lowering"[Title/Abstract] OR metformin[Title/Abstract] OR biguanide[Title/Abstract] OR insulin[Title/Abstract] OR sulfonylurea[Title/Abstract] OR sulphonylurea[Title/Abstract] OR glimepiride[Title/Abstract] OR glipizide[Title/Abstract] OR gliclazide[Title/Abstract] OR glyburide[Title/Abstract] OR glibenclamide[Title/Abstract] OR meglitinide[Title/Abstract] OR repaglinide[Title/Abstract] OR nateglinide[Title/Abstract] OR thiazolidinedione[Title/Abstract] OR glitazone[Title/Abstract] OR pioglitazone[Title/Abstract] OR rosiglitazone[Title/Abstract] OR "DPP-4 inhibitor"[Title/Abstract] OR "DPP-4 inhibitors"[Title/Abstract] OR DPP4[Title/Abstract] OR gliptin[Title/Abstract] OR sitagliptin[Title/Abstract] OR saxagliptin[Title/Abstract] OR linagliptin[Title/Abstract] OR alogliptin[Title/Abstract] OR vildagliptin[Title/Abstract] OR "GLP-1 receptor agonist"[Title/Abstract] OR "GLP-1 receptor agonists"[Title/Abstract] OR GLP-1RA[Title/Abstract] OR GLP1RA[Title/Abstract] OR exenatide[Title/Abstract] OR liraglutide[Title/Abstract] OR semaglutide[Title/Abstract] OR dulaglutide[Title/Abstract] OR lixisenatide[Title/Abstract] OR albiglutide[Title/Abstract] OR tirzepatide[Title/Abstract] OR "SGLT2 inhibitor"[Title/Abstract] OR "SGLT2 inhibitors"[Title/Abstract] OR gliflozin[Title/Abstract] OR dapagliflozin[Title/Abstract] OR empagliflozin[Title/Abstract] OR canagliflozin[Title/Abstract] OR ertugliflozin[Title/Abstract] OR ipragliflozin[Title/Abstract] OR luseogliflozin[Title/Abstract] OR tofogliflozin[Title/Abstract] OR "alpha-glucosidase inhibitor"[Title/Abstract] OR "alpha-glucosidase inhibitors"[Title/Abstract] OR acarbose[Title/Abstract] OR miglitol[Title/Abstract] OR voglibose[Title/Abstract] OR pramlintide[Title/Abstract]) OR ("Hypolipidemic Agents"[Mesh] OR "Hydroxymethylglutaryl-CoA Reductase Inhibitors"[Mesh] OR "Ezetimibe"[Mesh] OR "Fenofibrate"[Mesh] OR "Gemfibrozil"[Mesh] OR "Niacin"[Mesh] OR "Fatty Acids, Omega-3"[Mesh] OR "Proprotein Convertase 9"[Mesh] OR "lipid-lowering"[Title/Abstract] OR "lipid lowering"[Title/Abstract] OR "cholesterol-lowering"[Title/Abstract] OR "cholesterol lowering"[Title/Abstract] OR hypolipidemic[Title/Abstract] OR hypolipidaemic[Title/Abstract] OR antilipemic[Title/Abstract] OR antilipaemic[Title/Abstract] OR antihyperlipidemic[Title/Abstract] OR antihyperlipidaemic[Title/Abstract] OR statin[Title/Abstract] OR "HMG-CoA reductase inhibitor"[Title/Abstract] OR "HMG-CoA reductase inhibitors"[Title/Abstract] OR atorvastatin[Title/Abstract] OR simvastatin[Title/Abstract] OR rosuvastatin[Title/Abstract] OR pravastatin[Title/Abstract] OR lovastatin[Title/Abstract] OR fluvastatin[Title/Abstract] OR pitavastatin[Title/Abstract] OR fibrate[Title/Abstract] OR fenofibrate[Title/Abstract] OR gemfibrozil[Title/Abstract] OR bezafibrate[Title/Abstract] OR clofibrate[Title/Abstract] OR ciprofibrate[Title/Abstract] OR ezetimibe[Title/Abstract] OR "PCSK9 inhibitor"[Title/Abstract] OR "PCSK9 inhibitors"[Title/Abstract] OR PCSK9i[Title/Abstract] OR alirocumab[Title/Abstract] OR evolocumab[Title/Abstract] OR inclisiran[Title/Abstract] OR "bile acid sequestrant"[Title/Abstract] OR "bile acid sequestrants"[Title/Abstract] OR cholestyramine[Title/Abstract] OR colestyramine[Title/Abstract] OR colestipol[Title/Abstract] OR colesevelam[Title/Abstract] OR niacin[Title/Abstract] OR "nicotinic acid"[Title/Abstract] OR bempedoic[Title/Abstract] OR "bempedoic acid"[Title/Abstract] OR "omega-3"[Title/Abstract] OR "omega 3"[Title/Abstract] OR "n-3 fatty acid"[Title/Abstract] OR "n-3 fatty acids"[Title/Abstract] OR "fish oil"[Title/Abstract] OR icosapent[Title/Abstract] OR "icosapent ethyl"[Title/Abstract])) | **509** |
| **Embase** | ('psoriasis'/exp OR psoriasis:ti,ab,kw OR psoriases:ti,ab,kw OR psoriasiform:ti,ab,kw OR 'psoriasiform dermatitis':ti,ab,kw OR 'psoriasiform dermatosis':ti,ab,kw OR 'psoriasiform lesion':ti,ab,kw OR 'psoriasiform rash':ti,ab,kw OR 'psoriatic epidermis':ti,ab,kw OR 'psoriatic skin':ti,ab,kw OR 'pustular psoriasis':ti,ab,kw OR 'palmoplantar pustulosis':ti,ab,kw OR 'pustulosis palmaris et plantaris':ti,ab,kw OR 'pustulosis of palms and soles':ti,ab,kw) AND (('antidiabetic agent'/exp OR 'insulin'/exp OR 'metformin'/exp OR 'sulfonylurea derivative'/exp OR 'thiazolidinedione derivative'/exp OR 'dipeptidyl peptidase 4 inhibitor'/exp OR 'glucagon like peptide 1 receptor agonist'/exp OR 'sodium glucose cotransporter 2 inhibitor'/exp OR 'alpha glucosidase inhibitor'/exp OR 'amylin receptor agonist'/exp OR antidiabet:ti,ab,kw OR hypoglycemi:ti,ab,kw OR hypoglycaemi:ti,ab,kw OR antihyperglycemi:ti,ab,kw OR antihyperglycaemi:ti,ab,kw OR 'glucose-lowering':ti,ab,kw OR 'glucose lowering':ti,ab,kw OR metformin:ti,ab,kw OR biguanide:ti,ab,kw OR insulin:ti,ab,kw OR sulfonylurea:ti,ab,kw OR sulphonylurea:ti,ab,kw OR glimepiride:ti,ab,kw OR glipizide:ti,ab,kw OR gliclazide:ti,ab,kw OR glyburide:ti,ab,kw OR glibenclamide:ti,ab,kw OR meglitinide:ti,ab,kw OR repaglinide:ti,ab,kw OR nateglinide:ti,ab,kw OR thiazolidinedione:ti,ab,kw OR glitazone:ti,ab,kw OR pioglitazone:ti,ab,kw OR rosiglitazone:ti,ab,kw OR 'dpp-4 inhibitor':ti,ab,kw OR 'dpp-4 inhibitors':ti,ab,kw OR dpp4:ti,ab,kw OR gliptin:ti,ab,kw OR sitagliptin:ti,ab,kw OR saxagliptin:ti,ab,kw OR linagliptin:ti,ab,kw OR alogliptin:ti,ab,kw OR vildagliptin:ti,ab,kw OR 'glp-1 receptor agonist':ti,ab,kw OR 'glp-1 receptor agonists':ti,ab,kw OR glp-1ra:ti,ab,kw OR glp1ra:ti,ab,kw OR exenatide:ti,ab,kw OR liraglutide:ti,ab,kw OR semaglutide:ti,ab,kw OR dulaglutide:ti,ab,kw OR lixisenatide:ti,ab,kw OR albiglutide:ti,ab,kw OR tirzepatide:ti,ab,kw OR 'sglt2 inhibitor':ti,ab,kw OR 'sglt2 inhibitors':ti,ab,kw OR 'sglt-2 inhibitor':ti,ab,kw OR 'sglt-2 inhibitors':ti,ab,kw OR gliflozin:ti,ab,kw OR dapagliflozin:ti,ab,kw OR empagliflozin:ti,ab,kw OR canagliflozin:ti,ab,kw OR ertugliflozin:ti,ab,kw OR ipragliflozin:ti,ab,kw OR luseogliflozin:ti,ab,kw OR tofogliflozin:ti,ab,kw OR 'alpha-glucosidase inhibitor':ti,ab,kw OR 'alpha-glucosidase inhibitors':ti,ab,kw OR acarbose:ti,ab,kw OR miglitol:ti,ab,kw OR voglibose:ti,ab,kw OR pramlintide:ti,ab,kw) OR ('hypolipidemic agent'/exp OR 'antihyperlipidemic agent'/exp OR 'antilipemic agent'/exp OR 'hydroxymethylglutaryl coenzyme a reductase inhibitor'/exp OR 'pcsk9 inhibitor'/exp OR 'ezetimibe'/exp OR 'fenofibrate'/exp OR 'gemfibrozil'/exp OR 'nicotinic acid'/exp OR 'omega 3 fatty acid'/exp OR 'bempedoic acid'/exp OR 'lipid-lowering':ti,ab,kw OR 'lipid lowering':ti,ab,kw OR 'cholesterol-lowering':ti,ab,kw OR 'cholesterol lowering':ti,ab,kw OR hypolipidemic:ti,ab,kw OR hypolipidaemic:ti,ab,kw OR antilipemic:ti,ab,kw OR antilipaemic:ti,ab,kw OR antihyperlipidemic:ti,ab,kw OR antihyperlipidaemic:ti,ab,kw OR statin:ti,ab,kw OR 'hmg-coa reductase inhibitor':ti,ab,kw OR atorvastatin:ti,ab,kw OR simvastatin:ti,ab,kw OR rosuvastatin:ti,ab,kw OR pravastatin:ti,ab,kw OR lovastatin:ti,ab,kw OR fluvastatin:ti,ab,kw OR pitavastatin:ti,ab,kw OR fibrate:ti,ab,kw OR fenofibrate:ti,ab,kw OR gemfibrozil:ti,ab,kw OR bezafibrate:ti,ab,kw OR clofibrate:ti,ab,kw OR ciprofibrate:ti,ab,kw OR ezetimibe:ti,ab,kw OR 'pcsk9 inhibitor':ti,ab,kw OR pcsk9i:ti,ab,kw OR alirocumab:ti,ab,kw OR evolocumab:ti,ab,kw OR inclisiran:ti,ab,kw OR 'bile acid sequestrant':ti,ab,kw OR cholestyramine:ti,ab,kw OR colestyramine:ti,ab,kw OR colestipol:ti,ab,kw OR colesevelam:ti,ab,kw OR niacin:ti,ab,kw OR 'nicotinic acid':ti,ab,kw OR bempedoic:ti,ab,kw OR 'bempedoic acid':ti,ab,kw OR 'omega-3':ti,ab,kw OR 'omega 3':ti,ab,kw OR 'n-3 fatty acid':ti,ab,kw OR 'fish oil':ti,ab,kw OR icosapent:ti,ab,kw OR 'icosapent ethyl':ti,ab,kw)) | **2057** |
| **Web of Science Core Collection** | TS=(psoriasis OR psoriases OR psoriasiform OR "pustular psoriasis" OR "palmoplantar pustulosis" OR "pustulosis palmaris et plantaris" OR "pustulosis of palms and soles" OR "pustular psoriasis of palms and soles") AND TS=(antidiabet OR hypoglycemi OR hypoglycaemi OR antihyperglycemi OR antihyperglycaemi OR "glucose-lowering" OR "glucose lowering" OR metformin OR biguanide OR insulin OR sulfonylurea OR sulphonylurea OR glimepiride OR glipizide OR gliclazide OR glyburide OR glibenclamide OR meglitinide OR repaglinide OR nateglinide OR thiazolidinedione OR glitazone OR pioglitazone OR rosiglitazone OR "alpha-glucosidase inhibitor" OR acarbose OR miglitol OR voglibose OR "DPP-4 inhibitor" OR DPP4 OR "dipeptidyl peptidase-4 inhibitor" OR gliptin OR sitagliptin OR saxagliptin OR linagliptin OR alogliptin OR vildagliptin OR "GLP-1 receptor agonist" OR GLP-1RA OR GLP1RA OR "glucagon-like peptide-1 receptor agonist" OR exenatide OR liraglutide OR semaglutide OR dulaglutide OR lixisenatide OR albiglutide OR tirzepatide OR "SGLT2 inhibitor" OR "SGLT-2 inhibitor" OR gliflozin OR dapagliflozin OR empagliflozin OR canagliflozin OR ertugliflozin OR ipragliflozin OR luseogliflozin OR tofogliflozin OR pramlintide OR "lipid-lowering" OR "lipid lowering" OR "cholesterol-lowering" OR "cholesterol lowering" OR hypolipidemic OR hypolipidaemic OR antilipemic OR antilipaemic OR antihyperlipidemic OR antihyperlipidaemic OR statin OR "HMG-CoA reductase inhibitor" OR atorvastatin OR simvastatin OR rosuvastatin OR pravastatin OR lovastatin OR fluvastatin OR pitavastatin OR fibrate OR fenofibrate OR gemfibrozil OR bezafibrate OR clofibrate OR ciprofibrate OR ezetimibe OR "PCSK9 inhibitor" OR PCSK9i OR alirocumab OR evolocumab OR inclisiran OR "bile acid sequestrant" OR cholestyramine OR colestyramine OR colestipol OR colesevelam OR niacin OR "nicotinic acid" OR bempedoic OR "bempedoic acid" OR "omega-3" OR "omega 3" OR "n-3 fatty acid" OR "fish oil" OR icosapent OR "icosapent ethyl") | **1017** |
| **Cochrane Library** | ((MeSH descriptor: [Psoriasis] explode all trees) OR (psoriasis OR psoriases OR psoriasiform OR "pustular psoriasis" OR "palmoplantar pustulosis" OR "pustulosis palmaris et plantaris" OR "pustulosis of palms and soles" OR "pustular psoriasis of palms and soles"):ti,ab,kw) AND (((MeSH descriptor: [Hypoglycemic Agents] explode all trees) OR (antidiabet OR hypoglycemi OR hypoglycaemi OR antihyperglycemi OR antihyperglycaemi OR "glucose-lowering" OR "glucose lowering" OR metformin OR biguanide OR insulin OR sulfonylurea OR sulphonylurea OR glimepiride OR glipizide OR gliclazide OR glyburide OR glibenclamide OR meglitinide OR repaglinide OR nateglinide OR thiazolidinedione OR glitazone OR pioglitazone OR rosiglitazone OR "DPP-4 inhibitor" OR DPP4 OR gliptin OR sitagliptin OR saxagliptin OR linagliptin OR alogliptin OR vildagliptin OR "GLP-1 receptor agonist" OR GLP-1RA OR GLP1RA OR exenatide OR liraglutide OR semaglutide OR dulaglutide OR lixisenatide OR albiglutide OR tirzepatide OR "SGLT2 inhibitor" OR "SGLT-2 inhibitor" OR gliflozin OR dapagliflozin OR empagliflozin OR canagliflozin OR ertugliflozin OR ipragliflozin OR luseogliflozin OR tofogliflozin OR "alpha-glucosidase inhibitor" OR acarbose OR miglitol OR voglibose OR pramlintide):ti,ab,kw) OR ((MeSH descriptor: [Hypolipidemic Agents] explode all trees) OR ("lipid-lowering" OR "lipid lowering" OR "cholesterol-lowering" OR "cholesterol lowering" OR hypolipidemic OR hypolipidaemic OR antilipemic OR antilipaemic OR antihyperlipidemic OR antihyperlipidaemic OR statin OR "HMG-CoA reductase inhibitor" OR atorvastatin OR simvastatin OR rosuvastatin OR pravastatin OR lovastatin OR fluvastatin OR pitavastatin OR fibrate OR fenofibrate OR gemfibrozil OR bezafibrate OR clofibrate OR ciprofibrate OR ezetimibe OR "PCSK9 inhibitor" OR PCSK9i OR alirocumab OR evolocumab OR inclisiran OR "bile acid sequestrant" OR cholestyramine OR colestyramine OR colestipol OR colesevelam OR niacin OR "nicotinic acid" OR bempedoic OR "bempedoic acid" OR "omega-3" OR "omega 3" OR "n-3 fatty acid" OR "fish oil" OR icosapent OR "icosapent ethyl"):ti,ab,kw)) | **23** |

Note: Initial searches ran from database inception to 1 July 2026. On 11 September 2026, the same search strategies were rerun with a publication-year restriction to 2026, covering records available on that date. No language restriction was applied. The exact interface-specific date-filter expressions and search-history exports were not retained; the queries above must not be interpreted as reconstructed execution logs for the update.

### 2.1.2 Supplementary Table S2. Key effect estimates for glucose-lowering and lipid-lowering medications and their roles in the evidence framework

| **Report** | **Main comparison** | **Effect estimate (95% CI)** | **Outcome type** | **Comparator category** | **Evidence role** | **Presentation in the review** |
| --- | --- | --- | --- | --- | --- | --- |
| Chen WS 2025 | DPP-4i vs sulfonylurea | HR 0.422 (0.273–0.716) | Broader psoriasis-related outcome including PsA codes | Active drug comparator | Primary DPP-4i evidence | Figure 3 |
| Chen WS 2025 | TZD vs sulfonylurea | HR 0.347 (0.039–3.049) | Broader psoriasis-related outcome including PsA codes | Active drug comparator | Exploratory active-comparator evidence with sparse events | Supplementary Figure S4C |
| Chiang 2026 | GLP-1RA vs DPP-4i | HR 1.190 (1.110–1.280) | Study-reported incident psoriasis; restriction to cutaneous psoriasis unconfirmed. | Active drug comparator | Primary GLP-1RA estimate | Figure 3 |
| Huang 2021 | AGI plus metformin vs metformin alone | HR 8.770 (1.580–48.580) | Composite PsO/PsA outcome | Non-equivalent treatment-intensity comparison | Composite-outcome treatment-intensity evidence | Supplementary Figure S4C |
| Tseng 2023 | Metformin vs mixed non-metformin therapy | HR 0.898 (0.845–0.954) | Broad psoriasis-related outcome | Mixed drug comparator | Supporting evidence | Figure 4 |
| Hung 2025 | DPP-4i vs non-DPP-4i oral glucose-lowering therapy | HR 0.573 (0.428–0.687) | PsO-specific outcome | Mixed drug comparator | Supporting estimate from the Taiwan NHIRD evidence cluster | Figure 4 |
| Chiu 2024 | DPP-4i vs mixed non-DPP-4i therapy | HR 0.890 (0.870–0.910) | Broad PsO/PsA-coded outcome | Mixed drug comparator | Supporting estimate from a potentially overlapping database | Figure 4 |
| Chen YC 2020 | DPP-4i vs DPP-4i-naïve mixed therapy | HR 0.560 (0.520–0.610) | Broad post-index psoriasis-related diagnosis | Mixed drug comparator | Supporting estimate with limitations in baseline outcome ascertainment | Figure 4 |
| Wu CY 2015 | Frequent metformin use vs nonuse | OR 0.940 (0.880–0.990) | Broad PsO/PsA outcome | Drug nonuser comparison | Exposure-frequency supporting evidence | Supplementary Figure S2 |
| Wu CY 2015 | Frequent TZD use vs nonuse | OR 0.890 (0.810–0.980) | Broad PsO/PsA outcome | Drug nonuser comparison | Exposure-frequency supporting evidence | Supplementary Figure S2 |
| Wu CY 2015 | Frequent insulin use vs nonuse | OR 1.290 (1.180–1.420) | Broad PsO/PsA outcome | Drug nonuser comparison | Exposure-frequency supporting evidence | Supplementary Figure S2 |
| Ma KSK 2024 | SGLT2i vs DPP-4i | HR 1.080 (1.030–1.130) | Study-reported incident psoriasis; psoriasis specificity unconfirmed | Active drug comparator | Primary SGLT2i estimate | Figure 3 |
| Ma SH 2022 | Persistent SGLT2i use vs mixed non-SGLT2i therapy | HR 1.240 (0.950–1.640) | Study-reported incident psoriasis; restriction to cutaneous psoriasis unconfirmed | Mixed drug comparator | Supporting SGLT2i estimate | Figure 4 |
| Brauchli 2008 | Current TZD use with ≥5 prescriptions vs nonuse | OR 0.330 (0.160–0.660) | First-recorded psoriasis; restriction to cutaneous psoriasis unconfirmed | Non-active nonuser comparison | Exposure-timing and cumulative-prescription evidence | Supplementary Figure S2 |
| Brauchli 2008 | Current metformin use with ≥15 prescriptions vs nonuse | OR 0.770 (0.620–0.960) | First-recorded psoriasis; restriction to cutaneous psoriasis unconfirmed | Non-active nonuser comparison | Exposure-timing and cumulative-prescription evidence | Supplementary Figure S2 |
| Brauchli 2008 | Current sulfonylurea use with ≥15 prescriptions vs nonuse | OR 1.070 (0.880–1.310) | First-recorded psoriasis; restriction to cutaneous psoriasis unconfirmed | Non-active nonuser comparison | Supporting estimate with a confidence interval including the null | Supplementary Figure S2 |
| Brauchli 2008 | Current acarbose use with ≥5 prescriptions vs nonuse | OR 1.330 (0.560–3.160) | First-recorded psoriasis; restriction to cutaneous psoriasis unconfirmed | Non-active nonuser comparison | Exploratory estimate with sparse exposure | Supplementary Figure S2 |
| Ching 2025 | Any prior GLP-1RA exposure vs no GLP-1RA exposure | OR 0.410 (0.350–0.480) | Newly recorded psoriasis after exposure | Non-active comparator | Supporting estimate sensitive to time-zero and comparator definition | Figure 4 |
| Brauchli 2011 | Any statin use vs no lipid-lowering drug use | OR 1.030 (0.950–1.130) | First-recorded psoriasis; restriction to cutaneous psoriasis unconfirmed | Non-active nonuser comparison | Principal report-specific statin estimate | Supplementary Figure S3A |
| Han 2025 | >365 cumulative days of statin use vs nonuse | Reported OR 0.770 (0.730–0.820) | Broad PsO/PsA outcome | Non-active nonuser comparison | Supporting statin exposure-duration estimate; reported adjusted OR retained. The crude-to-adjusted reversal does not by itself establish a reporting error. | Supplementary Figure S3A |
| Chodick 2015 | PDC ≥80% vs PDC <20%, women | HR 0.880 (0.790–0.980) | Incident psoriasis | Adherence comparison | Statin adherence evidence | Supplementary Figure S3B |
| Chodick 2015 | PDC ≥80% vs PDC <20%, men | HR 1.000 (0.900–1.110) | Incident psoriasis | Adherence comparison | Statin adherence evidence | Supplementary Figure S3B |
| Karacabeyli 2026 | GLP-1RA vs DPP-4i | HR 1.180 (0.770–1.820) | Composite PsO/PsA outcome | Active drug comparator | Independent Canadian composite-outcome supporting estimate | Supplementary Figure S4A |
| Karacabeyli 2026 | SGLT2i vs DPP-4i | HR 1.210 (0.840–1.750) | Composite PsO/PsA outcome | Active drug comparator | Independent Canadian composite-outcome supporting estimate | Supplementary Figure S4A |
| Lee 2025 | GLP-1RA vs DPP-4i | HR 1.170 (1.120–1.220) | Study-reported incident psoriasis; psoriasis specificity unconfirmed | Active drug comparator | TriNetX replacement estimalternative estimate within the likely overlapping TriNetX US evidence cluster ate because of potential population overlap | Supplementary Figure S4A |
| Mahajan 2026 | DPP-4i vs GLP-1RA | HR 0.79 (0.70–0.85) | Study-reported incident psoriasis; restriction to cutaneous psoriasis unconfirmed. | Active drug comparator | Supporting active-comparator estimate within the potentially overlapping TriNetX evidence cluster | Supplementary Figure S4A |
| Mahajan 2026 | DPP-4i vs SGLT2i | HR 0.81 (0.70–0.91) | Study-reported incident psoriasis; restriction to cutaneous psoriasis unconfirmed. | Active drug comparator | Supporting active-comparator estimate | Supplementary Figure S4A |
| Mahajan 2026 | GLP-1RA vs SGLT2i | HR 1.02 (0.91–1.15) | Study-reported incident psoriasis; restriction to cutaneous psoriasis unconfirmed. | Active drug comparator | Supporting active-comparator estimate | Supplementary Figure S4A |

Note: Effect estimates are presented as reported in the original studies and were not transformed or pooled. HRs and ORs should be interpreted according to the corresponding report design, exposure definition, comparator, and outcome definition. Evidence roles were assigned in this review according to comparator type, methodological relevance, outcome specificity, reporting completeness, and the potential for overlap between report populations. Selected estimates were used for the main interpretation within each medication class. Supporting, exploratory, replacement, composite-outcome, exposure-intensity, and adherence estimates were retained to provide complementary information. These labels describe the role of each estimate in this review and do not represent a formal hierarchy of evidence or ROBINS-I judgment.

Abbreviations: AGI, alpha-glucosidase inhibitor; CI, confidence interval; DPP-4i, dipeptidyl peptidase-4 inhibitor; GLP-1RA, glucagon-like peptide-1 receptor agonist; HR, hazard ratio; NHIRD, National Health Insurance Research Database; OR, odds ratio; PDC, proportion of days covered; PsA, psoriatic arthritis; PsO, psoriasis; SGLT2i, sodium-glucose cotransporter-2 inhibitor; TZD, thiazolidinedione.

### 2.1.3 Supplementary Table S3. Overall ROBINS-I judgments for report-level assessment anchors and key result-specific limitations

| **Report** | **ROBINS-I assessment anchor** | **Overall ROBINS-I judgment** | **Key ROBINS-I concern(s)** | **Additional result-specific limitation(s)** | **Handling of related results from the same report** |
| --- | --- | --- | --- | --- | --- |
| Chen WS 2025 | DPP-4i vs sulfonylurea; broader psoriasis-related outcome including PsA codes | Serious | Residual confounding and residual covariate imbalance after matching | Psoriasis was identified using administrative diagnostic codes | The TZD vs sulfonylurea estimate shared most design-level judgments; sparse events, imprecision, and drug-specific residual confounding were considered separately |
| Chiang 2026 | GLP-1RA vs DPP-4i; study-reported incident psoriasis with unconfirmed cutaneous specificity. | Moderate | Residual prescribing-selection confounding | The outcome was defined using at least one ICD record | Other active-comparator estimates shared the same overall design framework; comparator-specific confounding was considered separately |
| Huang 2021 | AGI plus metformin vs metformin alone; composite PsO/PsA outcome | Serious | Selection related to persistent-use eligibility, non-equivalent treatment strategies, and residual confounding | Composite PsO/PsA outcome and sparse events | Analyses using alternative treatment-gap windows shared the principal assessment; imprecision was described separately |
| Tseng 2023 | Metformin vs mixed non-metformin therapy; broad psoriasis-related outcome | Serious | Confounding by indication, mixed comparator composition, and selection associated with the 12-month landmark design | Broad outcome based on ICD-9-CM code 696 | The per-protocol analysis shared most domain judgments; deviations from the assigned exposure strategy were considered separately |
| Hung 2025 | DPP-4i vs non-DPP-4i therapy; psoriasis-specific outcome | Serious | Non-new-user design, selection based on 90-day treatment persistence, and residual confounding from a mixed comparator group | Outcome ascertainment relied on administrative records | PsA and composite PsO/PsA results shared most judgments; differences in outcome measurement and directness were noted separately |
| Chiu 2024 | DPP-4i vs mixed non-DPP-4i therapy; broad PsO/PsA-coded outcome | Serious | Different index-date definitions between groups, time-zero bias, and residual confounding | The outcome definition included PsA diagnostic codes | Sensitivity analyses shared the principal assessment unless the analysis changed the eligible population or time-zero definition |
| Chen YC 2020 | DPP-4i vs DPP-4i-naïve mixed therapy; post-index psoriasis-related outcome | Serious | Residual confounding in a mixed-treatment comparison; uncertainty in baseline psoriasis exclusion and routine coded ascertainment. The outcome is a broader post-index diagnosis rather than confirmed incident cutaneous psoriasis. | The same repeated-diagnosis algorithm was reported for both groups, but psoriasis-specific validation and differential ascertainment were not established. | Related analyses using the same population and analytical framework shared the principal assessment |
| Wu CY 2015 | Frequent metformin use vs nonuse; broad PsO/PsA outcome | Serious | Confounding by indication and concomitant use of multiple glucose-lowering medications | Combined PsO/PsA outcome | TZD, insulin, and other drug-specific estimates shared several design-level judgments; drug-specific confounding was considered separately |
| Ma KSK 2024 | SGLT2i vs DPP-4i; study-reported incident psoriasis; restriction to cutaneous psoriasis unconfirmed | Moderate | Residual confounding despite extensive propensity-score matching; incomplete covariate data and unmeasured treatment-selection factors remain relevant. | The supplementary methods identify TriNetX data from 92 US healthcare organizations, prior-outcome exclusion, and an L40-coded psoriasis outcome. They do not explicitly establish exclusion of L40.5. | Related results should be assessed according to their specific comparison and outcome. Shared methods do not automatically establish identical bias across all results. |
| Ma SH 2022 | Persistent SGLT2i use vs mixed non-SGLT2i therapy; study-reported incident psoriasis; restriction to cutaneous psoriasis unconfirmed | Serious | Selection of persistent users and a never-user comparator remains a concern, but follow-up began after the three-month qualifying exposure period. For the explicitly landmark-based target, D2 was rated moderate in the revision-stage assessment; confounding by treatment indication remains a separate concern. | Prior psoriasis was excluded before index. The outcome algorithm required repeated specialist diagnoses or hospitalization, but restriction of L40 coding to cutaneous psoriasis was unconfirmed. | The kidney-disease subgroup requires separate consideration of subgroup selection and precision. The D2 judgment was reassessed against the reported eligibility and follow-up timeline during revision; the final judgment and rationale are presented in Tables S9A and S9E. |
| Brauchli 2008 | Current TZD use with ≥5 prescriptions vs nonuse; first-recorded psoriasis; restriction to cutaneous psoriasis unconfirmed | Serious | Confounding by indication and use of a general-population nonuser comparator | First-recorded psoriasis may not represent true disease onset; the estimate was imprecise | Metformin, sulfonylurea, acarbose, and other exposure-stratum estimates shared several design-level judgments; drug-specific confounding and exposure classification were considered separately |
| Ching 2025 | Any recorded GLP-1RA exposure vs no recorded GLP-1RA exposure; first-recorded psoriasis; restriction to cutaneous psoriasis unconfirmed | Serious | Time-zero selection and potential immortal-time bias | The detailed psoriasis diagnostic algorithm was not reported | Related analyses using the same exposure definition and cohort were considered in relation to the assessment-anchor judgment. |
| Brauchli 2011 | Any statin use vs no lipid-lowering drug use; first-recorded psoriasis; restriction to cutaneous psoriasis unconfirmed | Serious | Residual confounding by lipid-related indications and prescribing factors remains possible despite adjustment for recorded characteristics. | First-recorded psoriasis may not represent biological disease onset. The available code information did not establish restriction to cutaneous psoriasis. | Current, recent, past-use, and prescription-count analyses require attention to their own exposure definitions. Their timing categories differ from those in Brauchli 2008. |
| Han 2025 | Statin use for >365 days vs nonuse; broad PsO/PsA outcome | Serious | Residual confounding in a non-active-comparator analysis | Crude and adjusted estimates differed in direction; this does not by itself establish a reporting error; the outcome included PsA codes | The 1–365-day estimate shared the principal assessment and was interpreted qualitatively |
| Chodick 2015 | Women: PDC ≥80% vs PDC <20%; psoriasis; adjusted HR 0.88 (95% CI 0.79–0.98), original Table 3. This assessment anchor was specified retrospectively during revision | Serious | PDC was defined over follow-up without explicitly stopping accumulation at psoriasis onset, creating serious concern for exposure classification. Selection and adherence-related confounding require separate assessment; the actual analytical code was unavailable. | The implemented code was unavailable, so actual use of post-outcome information could not be verified. Sex-specific results did not establish a stable monotonic adherence gradient. | Exposure classification and deviations from the target treatment strategy require distinct rationales. Related sex-specific and PDC-category results should not automatically inherit every judgment without review. |
| Karacabeyli 2026 | GLP-1RA vs DPP-4i; composite PsO/PsA outcome | Moderate | Residual confounding and potential informative censoring in the as-treated analysis | PsO and PsA outcomes could not be separated | The SGLT2i vs DPP-4i estimate shared most design-level judgments; comparator-specific confounding was considered separately |
| Lee 2025 | GLP-1RA vs DPP-4i; study-reported incident psoriasis; psoriasis specificity unconfirmed | Moderate | Residual confounding, missing laboratory information, and differences in calendar period or follow-up | eMethod confirms L40; no explicit L40.5 exclusion. Supplementary Table 4 contains invalid intervals; see S10. | The estimate was retained as a potentially overlapping replacement result within the TriNetX evidence cluster |
| Mahajan 2026 | DPP-4i vs GLP-1RA; study-reported incident psoriasis with unconfirmed cutaneous specificity. | Moderate | Residual confounding related to diabetes severity and duration, obesity, socioeconomic or insurance factors, and incompletely captured clinical characteristics; selection associated with the 6-month landmark design | Psoriasis was identified from structured ICD-10-coded EHR data; missing BMI and other incompletely captured risk factors may contribute to residual confounding | DPP-4i vs SGLT2i and GLP-1RA vs SGLT2i estimates shared most design-level judgments; comparator-specific confounding was considered separately |

Note: ROBINS-I judgments were indexed to one report-level assessment anchor per included report. An assessment anchor identifies the population, exposure, comparator, outcome and model used for traceability; it is not necessarily the primary result of the original report or the result that dominates the synthesis. Related estimates require an explicit check of whether the same domain judgments apply, with separate assessment when population, exposure timing, comparator, outcome or analytical method changes.

### 2.1.4 Supplementary Table S4A. report design, time zero, and follow-up strategies

| **Report** | **report design** | **Index date/time zero** | **Washout or baseline period** | **Lag period** | **Primary analytical strategy** | **Primary follow-up endpoint** |
| --- | --- | --- | --- | --- | --- | --- |
| Chen WS 2025 | Time-stratified propensity score–matched active-comparator new-user cohort; target trial emulation | Exposed group: first prescription of the target medication. Control group: an index date randomly assigned with reference to the exposed group’s distribution of time from T2DM diagnosis to treatment. Alignment of treatment initiation and follow-up between groups was not clearly established. | Prior medication-use exclusion criteria defined according to the target trial framework | No additional lag period | 1:4 PSM; Cox regression; cluster-robust standard errors | 365 days after the index date |
| Chiang 2026 | Active-comparator new-user target trial emulation | First initiation date of GLP-1RA or DPP-4i therapy | Six-month washout period for both drug classes | 90 days | ITT analysis; 1:1 PSM; Cox regression | Up to 4 years |
| Huang 2021 | Retrospective matched cohort report | First date of metformin plus AGI or metformin-alone treatment | Required cumulative glucose-lowering treatment for ≥90 days | No uniform outcome lag period | Censoring based on 30-, 90-, and 180-day treatment-gap windows; Cox regression | Treatment discontinuation, outcome occurrence, or end of report |
| Tseng 2023 | Landmark cohort with a 12-month exposure-assessment period | End of the 12-month exposure-classification window after diabetes diagnosis | Initial 12 months used for exposure classification | None | IPTW; ITT primary analysis; per-protocol sensitivity analysis | Outcome occurrence, death, or end of 2011 |
| Hung 2025 | 1:4 matched retrospective cohort report | DPP-4i eligibility index date | Continuous DPP-4i use for ≥90 days; no explicit washout period reported | None | Multivariable Cox regression | Outcome occurrence or end of 2021 |
| Chiu 2024 | Propensity score–adjusted retrospective cohort report | First DPP-4i prescription date for the exposed group; T2DM diagnosis date for the comparator group | No new-user washout period reported | None | Propensity score–adjusted Cox regression | Outcome occurrence or end of 2019 |
| Chen YC 2020 | Prescription-time distribution matching plus 1:1 propensity score–matched cohort | First DPP-4i prescription date; simulated index date assigned to comparators | Pre-index incretin-based therapy excluded; washout duration not fully reported | None | PSM; multivariable Cox regression | Outcome occurrence or end of 2013 |
| Wu CY 2015 | Nested case-control report within a diabetes cohort | Date of first psoriasis or PsA diagnosis among cases | Participants with psoriasis or PsA before cohort entry were excluded | Not applicable | Conditional logistic regression | Three-year exposure window before the index date |
| Ma KSK 2024 | Propensity score–matched active-comparator treatment-initiator cohort | Date of SGLT2i or DPP-4i initiation | Prior use of the opposite comparator drug was excluded. Covariates were assessed during the 12 months before index. Patients with a prior diagnosis of the outcome were excluded from the corresponding outcome analysis. A fixed-duration psoriasis lookback or class-specific medication washout interval was not specified. | Not reported | ITT analysis; PSM; Cox regression | Up to 5 years |
| Ma SH 2022 | Matched cohort restricted to persistent medication users | Date after completion of 3 consecutive months of SGLT2i use | SGLT2i use for ≥28 days per month and continuously for ≥3 months | None | 1:2 matching; competing-risk model | Outcome occurrence, withdrawal from the NHI program, or end of 2018 |
| Brauchli 2008 | 1:1 matched case-control report | First psoriasis diagnosis; controls assigned the same index date | At least 3 years of pre-index GPRD records; prior psoriasis excluded | Not applicable | Conditional logistic regression | Prescription exposure before the index date |
| Ching 2025 | Retrospective cohort analysis using All of Us EHR data | For exposed patients, first GLP-1RA exposure preceded the psoriasis diagnosis; all included psoriasis diagnoses occurred after T2DM; no equivalent medication index for unexposed patients was reported | No standard medication washout period reported | No uniform lag period | Multivariable logistic regression | No uniform person-time model was used |
| Brauchli 2011 | 1:1 matched case-control report | Date of first-recorded psoriasis | At least 3 years of pre-index GPRD records | Not applicable | Conditional logistic regression | Lipid-lowering medication exposure before the index date |
| Han 2025 | Matched case-control report nested within a health-screening cohort | Date of first psoriasis-related diagnosis | Outcome washout period during 2002–2003 | Not applicable | 1:4 matching; overlap-weighted logistic regression | Cumulative statin-use days during the 2 years before the index date |
| Chodick 2015 | Retrospective cohort of new statin users | Date of first statin dispensing | At least 1 year of continuous MHS enrollment; prior PsO or PsA excluded | None | Cox regression; PDC classified over the full follow-up period | Outcome occurrence, death, disenrollment, or end of 2010 |
| Karacabeyli 2026 | Active-comparator new-user cohort | Date of GLP-1RA, SGLT2i, or DPP-4i initiation | Common 12-month washout period for all three drug classes; 2 years of continuous enrollment | None | Matching weights; as-treated primary analysis | Treatment discontinuation, switching, outcome occurrence, or end of 2023 |
| Lee 2025 | Active-comparator new-user ITT cohort | First prescription date of GLP-1RA or DPP-4i | Six-month washout period for both drug classes; 2-year covariate-assessment window | 90 days | 1:1 PSM; Cox regression | Last available record or January 1, 2025 |
| Mahajan 2026 | Active-comparator new-user emulated target trials using TriNetX EHR data | First prescription of DPP-4i, GLP-1RA, or SGLT2i within 1 year after a new T2DM diagnosis | Main report excludes prior autoimmune disease and other study-agent use before/on index; supplementary cohort note specifies a 12-month observation period for prior T2DM and autoimmune diagnoses | 6-month landmark after the index date | ITT analysis; 1:1 PSM; Cox regression; Benjamini–Hochberg false-discovery-rate adjustment | Outcome surveillance begins at day 183 after first prescription; endpoint is day 1095 from the prescription, not from the landmark (supplementary Table 2); censor at outcome, database withdrawal or death |

### 2.1.5 Supplementary Table S4B. Confounding control and statistical methods

| **Report** | **Matching or weighting method** | **Major adjustment factors** | **Effect measure** | **Handling of competing risks or repeated observations** | **Key methodological notes** |
| --- | --- | --- | --- | --- | --- |
| Chen WS 2025 | 1:4 PSM | Demographic characteristics, diabetes complications, comorbidities, medications, and healthcare utilization | HR | Cluster-robust standard errors for matched sets | Several healthcare-utilization variables remained imbalanced after matching |
| Chiang 2026 | 1:1 PSM | BMI, HbA1c, eGFR, comorbidities, medications, and lifestyle factors | HR | Competing-risk analysis not reported | Extensive sensitivity analyses and positive and negative control analyses were conducted |
| Huang 2021 | 1:10 matching on age, sex, and index date | Comorbidities and concomitant medications | HR | No inverse probability of censoring weighting | Only six primary outcome events occurred |
| Tseng 2023 | IPTW | Demographic characteristics, comorbidities, medications, and diabetes complications | HR | ITT and per-protocol analyses | The non-metformin comparator group was highly heterogeneous |
| Hung 2025 | 1:4 matching on age, sex, and index date | Comorbidities, geographic region, urbanization level, and healthcare-facility level | HR | Not adequately reported | Matching variables were limited |
| Chiu 2024 | Propensity score adjustment | Age, sex, comorbidities, Charlson Comorbidity Index, and related covariates | HR | Not adequately reported | Index-date definitions differed between the two groups |
| Chen YC 2020 | 1:1 PSM | Diabetes duration, comorbidities, glucose-lowering medications, statins, and related factors | HR | Not reported | Good balance was achieved for measured covariates |
| Wu CY 2015 | Risk-set matching plus propensity score matching | Comorbidities, diabetes duration, number of healthcare visits, and other medications | OR | Conditional logistic regression | The same patient could have been exposed to multiple glucose-lowering medications |
| Ma KSK 2024 | PSM | Covariates were not fully reported in the main text | HR | Not reported | Main text abbreviated; consult supplied online Methods and baseline table. Residual confounding remains possible |
| Ma SH 2022 | 1:2 PSM | Diabetes duration, insulin use, comorbidities, and concomitant medications | HR | Death was treated as a competing risk | Follow-up duration differed between the two groups |
| Brauchli 2008 | 1:1 matching on age, sex, practice, calendar time, and years of GPRD history | BMI, smoking, dermatologic/infectious and cardiovascular conditions, selected medications, and other antidiabetics; Table III footnote | OR | Conditional logistic regression | Most general-population nonusers did not have diabetes |
| Ching 2025 | Multivariable adjustment | Age, race, gender, income, smoking, weight, BMI, HbA1c, diabetic complications, adverse social determinants, and prior antidiabetics; Table I footnote | OR | No time-to-event model was used | Susceptible to time-zero and potential immortal-time bias |
| Brauchli 2011 | Individual matching | BMI, smoking, diabetes, cardiovascular risk factors, and related covariates | OR | Conditional logistic regression | Based on the same underlying source population as Brauchli 2008 |
| Han 2025 | Exact matching plus case-status-based overlap weighting | BMI, smoking, alcohol consumption, blood glucose, Charlson Comorbidity Index, and related factors | OR | None reported | Crude and adjusted effect estimates differed in direction; this alone does not establish a reporting error |
| Chodick 2015 | Multivariable Cox regression | Socioeconomic status, BMI, smoking, lipid levels, number of healthcare visits, and related factors | HR | Sex-stratified analyses | PDC may have incorporated medication information occurring after outcome onset |
| Karacabeyli 2026 | Generalized propensity score matching weights | Demographic characteristics, comorbidities, medications, healthcare utilization, and related factors | HR | Cluster-robust standard errors at the patient level | The unit of analysis was the treatment episode |
| Lee 2025 | 1:1 PSM; eMethod 2 describes greedy nearest-neighbour matching, caliper 0.1 pooled SD of propensity scores | Demographic characteristics, BMI, HbA1c, eGFR, comorbidities, medications, and related factors | HR | Competing-risk analysis not reported | Missing laboratory values were handled using categorical indicators and indicators of whether testing had been performed |
| Mahajan 2026 | 1:1 PSM using greedy nearest-neighbor matching | Age, sex, race/ethnicity, obesity/overweight, hypertension, smoking, infectious conditions, comorbidities, medication use, HbA1c, eGFR, and other prespecified clinical covariates | HR | Death was treated as a censoring event; no competing-risk model was reported | Baseline covariates were well balanced after matching (SMD <0.10); a 6-month landmark and positive/negative control analyses were used; residual confounding and missing clinical data remained possible |

**Abbreviations:** AGI, alpha-glucosidase inhibitor; BMI, body mass index; CCI, Charlson Comorbidity Index; DPP-4i, dipeptidyl peptidase-4 inhibitor; eGFR, estimated glomerular filtration rate; EHR, electronic health record; GLP-1RA, glucagon-like peptide-1 receptor agonist; GPRD, General Practice Research Database; HbA1c, glycated hemoglobin; HR, hazard ratio; IPCW, inverse probability of censoring weighting; IPTW, inverse probability of treatment weighting; ITT, intention-to-treat; MHS, Maccabi Healthcare Services; NHI, National Health Insurance; OR, odds ratio; PDC, proportion of days covered; PSM, propensity score matching; PsA, psoriatic arthritis; PsO, psoriasis; SE, standard error; SGLT2i, sodium-glucose cotransporter-2 inhibitor; SU, sulfonylurea; T2DM, type 2 diabetes mellitus; TZD, thiazolidinedione.

### 2.1.6 Supplementary Table S5. Exposure, comparator, and outcome definitions of the included reports

| **report** | **Exposure definition** | **Comparator** | **Comparator category** | **Outcome definition** | **Inclusion of PsA** | **Baseline outcome exclusion clearly defined** |
| --- | --- | --- | --- | --- | --- | --- |
| Chen WS 2025 | New initiation of DPP-4i or TZD | New initiation of sulfonylurea | Active drug comparator | At least two visits with ICD-9-CM codes 696.0, 696.1, 696.5, or 696.8 within 365 days after the index date. | Yes, at the outcome-definition level: ICD-9-CM 696.0 was explicitly included. PsA event counts were not reported separately. | Unclear for the medication-initiation baseline. Psoriasis diagnosed before T2DM diagnosis was excluded, but exclusion of all psoriasis recorded before medication initiation was not clearly established. |
| Chiang 2026 | New initiation of GLP-1RA | New initiation of DPP-4i | Active drug comparator | At least one L40-coded psoriasis diagnosis after a 90-day lag period; exclusion of arthropathic psoriasis codes was not explicitly documented. | Unclear; exclusion of arthropathic psoriasis codes from the L40 definition was not explicitly documented. | Yes |
| Huang 2021 | Metformin plus AGI | Metformin monotherapy | Non-equivalent treatment-intensity comparison | At least one inpatient or three outpatient diagnoses of PsO or PsA | Yes; composite outcome | Yes |
| Tseng 2023 | Metformin use during the initial 12-month exposure-assessment period | Other non-metformin glucose-lowering therapies | Mixed drug comparator | ICD-9-CM code 696 | May include PsA | Yes |
| Hung 2025 | Continuous DPP-4i use for at least 90 days | Non-DPP-4i oral glucose-lowering therapy | Mixed drug comparator | PsO: ICD-9-CM 696.1; ICD-10-CM L40.0, L40.4, L40.9. PsA was defined separately using 696.0 and L40.5-family codes. | Selected PsO codes exclude the stated PsA codes; PsA and composite results retained separately | Yes |
| Chiu 2024 | At least one DPP-4i prescription | Never use of DPP-4i | Mixed drug comparator | At least two broad psoriasis-related diagnostic codes | Yes | Yes |
| Chen YC 2020 | DPP-4i prescription use | DPP-4i-naïve mixed therapy | Mixed drug comparator | At least two broad psoriasis-related diagnostic codes | Yes | Not confirmed in the main report |
| Wu CY 2015 | At least 90 prescription days per year | Nonuse of the target medication | Exposure-frequency/nonuser comparison | First PsO or PsA diagnosis, defined by hospitalization or three consecutive specialist diagnoses | Yes | Yes |
| Ma KSK 2024 | New initiation of SGLT2i | New initiation of DPP-4i | Active drug comparator | Study-reported incident psoriasis identified using ICD-10 code L40; exclusion of arthropathic psoriasis codes was not explicitly documented. | Unconfirmed: eMethod specifies L40, without an explicit L40.5 exclusion. | Yes |
| Ma SH 2022 | SGLT2i use for at least 28 days per month and continuously for at least 3 months | Never use of SGLT2i | Mixed drug comparator | ICD-9-CM 696.1 or ICD-10 L40; at least three specialist outpatient diagnoses or one inpatient diagnosis | Restriction to cutaneous psoriasis unconfirmed; exclusion of the L40.5 branch was not documented | Prior psoriasis before the index date excluded; fixed-duration psoriasis lookback not specified in the main report |
| Brauchli 2008 | Current use: last prescription within 89 days before index; past use: at least 90 days before index. Prescription-count strata were also reported. | Nonuse of the target medication | Non-active nonuser comparison | First-recorded psoriasis identified using OXMIS/Read codes, with at least three years of prior GPRD records. Restriction to cutaneous psoriasis was unconfirmed. | Unconfirmed. A complete diagnostic code list was not reported, and restriction to cutaneous psoriasis could not be established. | Yes. Patients with prior psoriasis or a psoriasis-history code were excluded. |
| Ching 2025 | Any recorded GLP-1RA exposure | No GLP-1RA exposure | Non-active comparator | First-recorded psoriasis after T2DM and, for exposed patients, after first GLP-1RA exposure | Diagnostic algorithm not reported | Psoriasis diagnoses predating T2DM or, for exposed patients, first GLP-1RA exposure were excluded |
| Brauchli 2011 | Any statin use; current use: less than 30 days before index; recent use: 30–89 days before index; past use: at least 90 days before index. Prescription-count strata were also reported. | No lipid-lowering medication use | Non-active nonuser comparison | First-recorded psoriasis identified using OXMIS/Read codes, with at least three years of prior GPRD records. Restriction to cutaneous psoriasis was unconfirmed. | Unconfirmed. Exclusion of PsA from the case-identification algorithm was not established. | Yes. Patients with prior psoriasis or a psoriasis-history code were excluded. |
| Han 2025 | Cumulative days of statin use during the 2 years before the index date | Zero days of statin use | Non-active nonuser comparison | At least two broad PsO/PsA diagnostic codes | Yes | Psoriasis recorded during the fixed calendar period 2002–2003 was excluded; this was not a rolling 2-year pre-index washout for each participant. |
| Chodick 2015 | PDC calculated over the full follow-up period | Statin initiators with PDC <20% | Adherence comparison | ICD-9 696.1 recorded by a dermatologist or rheumatologist, or hospitalization record | Primarily PsO | Yes |
| Karacabeyli 2026 | New initiation of GLP-1RA or SGLT2i | New initiation of DPP-4i | Active drug comparator | Composite psoriatic disease outcome comprising PsO or PsA | Yes; PsO and PsA could not be separated | Ten-year baseline exclusion period |
| Lee 2025 | New initiation of GLP-1RA | New initiation of DPP-4i | Active drug comparator | Study-reported incident psoriasis from day 90; eMethod: UMLS:ICD10CM:L40 for outcome and baseline exclusion; no explicit L40.5 exclusion. | Unconfirmed: eMethod specifies L40, without an explicit L40.5 exclusion. | Yes |
| Mahajan 2026 | New initiation/first prescription of DPP-4i, GLP-1RA, or SGLT2i monotherapy | Pairwise initiators of GLP-1RA, SGLT2i, or DPP-4i | Active drug comparator | Study-reported incident psoriasis identified using ICD-10-CM code L40 after a 6-month landmark; restriction to cutaneous psoriasis remained unconfirmed. | Unclear; psoriasis and PsA were reported separately, but mutual exclusivity of the coding definitions was not established. | Any autoimmune disease before/on index excluded in main report; supplementary cohort note specifies a 12-month observation period for prior T2DM and autoimmune diagnoses |

**Note:** Exposure, comparator, and outcome definitions are presented as reported in the original studies. Comparator categories were assigned in this review according to the clinical meaning of the comparison and the underlying report design. “Baseline outcome exclusion clearly defined” indicates whether the report explicitly described exclusion of pre-existing psoriasis or psoriatic arthritis, including the diagnostic criteria, lookback interval, and temporal anchor when reported. Medication washout periods, covariate-assessment windows, and post-index outcome lags are recorded separately and are not substitutes for baseline outcome exclusion. Broad psoriasis-related coding algorithms may have included PsA or other psoriatic disease diagnoses even when the outcome was described as psoriasis.

**Abbreviations:** AGI, alpha-glucosidase inhibitor; DPP-4i, dipeptidyl peptidase-4 inhibitor; GLP-1RA, glucagon-like peptide-1 receptor agonist; GPRD, General Practice Research Database; ICD, International Classification of Diseases; PDC, proportion of days covered; PsA, psoriatic arthritis; PsO, psoriasis; SGLT2i, sodium-glucose cotransporter-2 inhibitor; TZD, thiazolidinedione.

### 2.1.7 Supplementary Table S6A. Selected report-specific effect estimates used in the evidence synthesis

| **Report** | **Medication/comparison** | **Outcome** | **Effect measure** | **Effect estimate** | **95% CI** | **Evidence role** | **Presentation in this review** |
| --- | --- | --- | --- | --- | --- | --- | --- |
| Chen WS 2025 | DPP-4i vs sulfonylurea | Broader psoriasis-related outcome including PsA codes | HR | 0.422 | 0.273–0.716 | Selected active-comparator estimate | Yes; presented separately |
| Chen WS 2025 | TZD vs sulfonylurea | Broader psoriasis-related outcome including PsA codes | HR | 0.347 | 0.039–3.049 | Exploratory estimate with sparse events | Yes; presented separately |
| Chiang 2026 | GLP-1RA vs DPP-4i | Study-reported incident psoriasis; restriction to cutaneous psoriasis unconfirmed. | HR | 1.19 | 1.110–1.280 | Selected GLP-1RA active-comparator estimate | Yes |
| Huang 2021 | AGI plus metformin vs metformin | Composite PsO/PsA outcome | HR | 8.77 | 1.580–48.580 | Exploratory composite-outcome evidence | Not pooled |
| Tseng 2023 | Metformin vs mixed non-metformin therapy | Broad psoriasis-related outcome (ICD-9-CM code 696) | HR | 0.898 | 0.845–0.954 | Supporting mixed-comparator evidence | Not pooled |
| Hung 2025 | DPP-4i vs non-DPP-4i therapy | PsO | HR | 0.573 | 0.428–0.687 | Supporting estimate from a potentially overlapping Taiwan database | Not pooled |
| Chiu 2024 | DPP-4i vs non-DPP-4i therapy | Broad PsO/PsA outcome | HR | 0.89 | 0.870–0.910 | Supporting estimate from a potentially overlapping Taiwan database | Not pooled |
| Chen YC 2020 | DPP-4i vs DPP-4i-naïve therapy | Broad psoriasis-related outcome | HR | 0.56 | 0.520–0.610 | Supporting evidence with limitations in baseline outcome ascertainment | Not pooled |
| Wu CY 2015 | Frequent metformin use vs nonuse | Broad PsO/PsA outcome | OR | 0.94 | 0.880–0.990 | Exposure-frequency evidence | Not pooled |
| Wu CY 2015 | Frequent TZD use vs nonuse | Broad PsO/PsA outcome | OR | 0.89 | 0.810–0.980 | Exposure-frequency evidence | Not pooled |
| Wu CY 2015 | Frequent insulin use vs nonuse | Broad PsO/PsA outcome | OR | 1.29 | 1.180–1.420 | Exposure-frequency evidence | Not pooled |
| Ma KSK 2024 | SGLT2i vs DPP-4i | Study-reported incident psoriasis; psoriasis specificity unconfirmed | HR | 1.08 | 1.030–1.130 | Selected SGLT2i active-comparator estimate | Yes |
| Ma SH 2022 | SGLT2i vs mixed non-SGLT2i therapy | Study-reported incident psoriasis; cutaneous specificity unconfirmed | HR | 1.24 | 0.950–1.640 | Supporting mixed-comparator evidence | Not pooled |
| Brauchli 2008 | Current TZD use with ≥5 prescriptions vs nonuse | First-recorded psoriasis; restriction to cutaneous psoriasis unconfirmed | OR | 0.33 | 0.160–0.660 | Parallel exposure-stratum result | Presented with the complete current/past TZD prescription strata in S6B; not pooled |
| Brauchli 2008 | Current metformin use with ≥15 prescriptions vs nonuse | First-recorded psoriasis; restriction to cutaneous psoriasis unconfirmed | OR | 0.77 | 0.620–0.960 | Exposure-timing evidence | Not pooled |
| Brauchli 2008 | Current sulfonylurea use with ≥15 prescriptions vs nonuse | First-recorded psoriasis; restriction to cutaneous psoriasis unconfirmed | OR | 1.07 | 0.880–1.310 | Supplementary estimate with a confidence interval including 1 | Not pooled |
| Brauchli 2008 | Current acarbose use with ≥5 prescriptions vs nonuse | First-recorded psoriasis; restriction to cutaneous psoriasis unconfirmed | OR | 1.33 | 0.560–3.160 | Evidence based on sparse exposure | Not pooled |
| Ching 2025 | Any GLP-1RA exposure vs no GLP-1RA exposure | First-recorded psoriasis; cutaneous specificity unconfirmed | OR | 0.41 | 0.350–0.480 | Supporting non-active-comparator evidence | Not pooled |
| Brauchli 2011 | Any statin use vs no lipid-lowering drug use | First-recorded psoriasis; restriction to cutaneous psoriasis unconfirmed | OR | 1.03 | 0.950–1.130 | Selected report-specific statin estimate | Not pooled |
| Han 2025 | Statin use for >365 days vs nonuse | Broad PsO/PsA outcome | OR | 0.77 | 0.730–0.820 | Parallel exposure-stratum result | Presented with the 1–365-day duration stratum; interpreted qualitatively and not pooled |
| Karacabeyli 2026 | GLP-1RA vs DPP-4i | Composite PsO/PsA outcome | HR | 1.18 | 0.770–1.820 | Active-comparator composite-outcome evidence | Not combined with PsO-specific estimates |
| Karacabeyli 2026 | SGLT2i vs DPP-4i | Composite PsO/PsA outcome | HR | 1.21 | 0.840–1.750 | Active-comparator composite-outcome evidence | Not combined with PsO-specific estimates |
| Lee 2025 | GLP-1RA vs DPP-4i | Study-reported incident psoriasis; psoriasis specificity unconfirmed | HR | 1.17 | 1.120–1.220 | Alternative estimate within the likely overlapping TriNetX US evidence cluster within the TriNetX evidence cluster | Not included simultaneously with Chiang 2026 |
| Mahajan 2026 | DPP-4i vs GLP-1RA | Study-reported incident psoriasis; restriction to cutaneous psoriasis unconfirmed. | HR | 0.79 | 0.70–0.85 | Supporting active-comparator estimate within the potentially overlapping TriNetX evidence cluster | Not pooled; interpreted as supporting evidence |
| Mahajan 2026 | DPP-4i vs SGLT2i | Study-reported incident psoriasis; restriction to cutaneous psoriasis unconfirmed. | HR | 0.81 | 0.70–0.91 | Supporting active-comparator estimate | Not pooled; interpreted separately |
| Mahajan 2026 | GLP-1RA vs SGLT2i | Study-reported incident psoriasis; restriction to cutaneous psoriasis unconfirmed. | HR | 1.02 | 0.91–1.15 | Supporting active-comparator estimate | Not pooled; interpreted separately |

### 2.1.8 Supplementary Table S6B. Alternative, sensitivity, secondary-outcome, exposure-timing, adherence, and subgroup estimates

| **report** | **Alternative or supplementary analysis** | **Effect estimate (95% CI)** | **Purpose in the review** |
| --- | --- | --- | --- |
| Huang 2021 | 90-day treatment-gap window | HR 4.580 (1.510–13.890) | Exposure-window sensitivity analysis |
| Huang 2021 | 180-day treatment-gap window | HR 3.290 (1.160–9.360) | Exposure-window sensitivity analysis |
| Tseng 2023 | Per-protocol analysis | HR 0.768 (0.714–0.826) | Alternative analysis |
| Hung 2025 | PsA outcome | HR 0.630 (0.462–0.751) | Secondary-outcome analysis |
| Hung 2025 | Composite psoriatic disease outcome | HR 0.583 (0.430–0.696) | Composite-outcome analysis |
| Chiu 2024 | DPP-4i monotherapy | HR 0.860 (0.840–0.880) | Sensitivity analysis |
| Chiu 2024 | Exclusion of patients with prior other chronic inflammatory skin diseases (CISDs) | HR 0.930 (0.900–0.960) | Sensitivity analysis |
| Chiu 2024 | Additional adjustment for diabetes duration | HR 0.830 (0.780–0.870) | Sensitivity analysis |
| Chiang 2026 | GLP-1RA vs insulin | HR 1.170 (1.100–1.250) | Supporting within-report comparison |
| Chiang 2026 | GLP-1RA vs biguanides | HR 1.140 (1.070–1.230) | Supporting within-report comparison |
| Chiang 2026 | GLP-1RA vs sulfonylurea | HR 1.340 (1.200–1.500) | Supporting within-report comparison |
| Chiang 2026 | GLP-1RA vs SGLT2i | HR 0.990 (0.910–1.070) | Supporting within-report comparison |
| Chiang 2026 | GLP-1RA vs TZD | HR 1.050 (0.640–1.710) | Supporting within-report comparison |
| Brauchli 2011 | Current statin use, 1–4 prescriptions | OR 0.600 (0.450–0.800) | Exposure-timing and prescription-count analysis |
| Brauchli 2011 | Current statin use, 5–19 prescriptions | OR 1.000 (0.840–1.180) | Exposure-timing and prescription-count analysis |
| Brauchli 2011 | Current statin use, ≥20 prescriptions | OR 1.080 (0.920–1.280) | Exposure-timing and prescription-count analysis |
| Brauchli 2011 | Recent statin use, 1–4 prescriptions | OR 1.000 (0.730–1.370) | Exposure-timing and prescription-count analysis |
| Brauchli 2011 | Recent statin use, 5–19 prescriptions | OR 1.130 (0.920–1.390) | Exposure-timing and prescription-count analysis |
| Brauchli 2011 | Recent statin use, ≥20 prescriptions | OR 1.020 (0.810–1.290) | Exposure-timing and prescription-count analysis |
| Brauchli 2011 | Past statin use, 1–4 prescriptions | OR 1.390 (1.090–1.780) | Exposure-timing and prescription-count analysis |
| Brauchli 2011 | Past statin use, 5–19 prescriptions | OR 1.100 (0.800–1.510) | Exposure-timing and prescription-count analysis |
| Brauchli 2011 | Past statin use, ≥20 prescriptions | OR 0.790 (0.450–1.380) | Exposure-timing and prescription-count analysis |
| Chodick 2015 | Women: PDC 20–39% vs PDC <20% | HR 0.910 (0.800–1.030) | Parallel exposure-stratum result |
| Chodick 2015 | Women: PDC 40–59% vs PDC <20% | HR 0.740 (0.650–0.850) | Parallel exposure-stratum result |
| Chodick 2015 | Women: PDC 60–79% vs PDC <20% | HR 0.780 (0.690–0.880) | Parallel exposure-stratum result |
| Chodick 2015 | Women: PDC ≥80% vs PDC <20% | HR 0.880 (0.790–0.980) | Parallel exposure-stratum result |
| Chodick 2015 | Men: PDC 20–39% vs PDC <20% | HR 0.900 (0.790–1.030) | Parallel exposure-stratum result |
| Chodick 2015 | Men: PDC 40–59% vs PDC <20% | HR 0.840 (0.740–0.950) | Parallel exposure-stratum result |
| Chodick 2015 | Men: PDC 60–79% vs PDC <20% | HR 0.870 (0.770–0.980) | Parallel exposure-stratum result |
| Chodick 2015 | Men: PDC ≥80% vs PDC <20% | HR 1.000 (0.900–1.110) | Parallel exposure-stratum result |
| Han 2025 | Statin use for 1–365 days vs nonuse | Reported OR 0.700 (0.660–0.740) | Parallel exposure-stratum result |
| Ma SH 2022 | Subgroup with kidney disease | HR 2.730 (1.450–5.130) | Exploratory subgroup analysis; interaction *P* value not reported |
| Lee 2025 | 6-month lag; GLP-1RA vs DPP-4i | HR 1.21 (1.16–1.27) | Supplement Table 2; events differ unexpectedly from main lag analysis; see S10 |
| Lee 2025 | Alternative matching models 1 / 2 / 3 | HR 1.23 (1.19–1.29) / 1.23 (1.18–1.28) / 1.20 (1.16–1.25) | Supplement Table 3; alternative covariate models, not independent replications |
| Lee 2025 | Per-treatment design; GLP-1RA vs DPP-4i | HR 1.35 (1.29–1.42) | Supplement Table 5; distinct adherence/censoring estimand |
| Lee 2025 | Exclude immune-related medication before index | HR 1.20 (1.15–1.26) | Supplement Table 6; restricted cohort |
| Lee 2025 | GLP-1RA vs nonuse: 3 months–2 / 4 / 6 / 8 years | HR 1.20 (1.09–1.32) / 1.27 (1.18–1.37) / 1.27 (1.19–1.36) / 1.59 (1.49–1.69) | Supplement Table 8; separate nonuser comparator. Eight-year row marked for proportional-hazards assumption P<0.05 |
| Lee 2025 | Semaglutide / liraglutide / dulaglutide vs DPP-4i | HR 1.25 (1.18–1.33) / 1.21 (1.14–1.30) / 1.22 (1.16–1.29) | Supplement Table 9; within-report drug-subtype analyses |
| Brauchli 2008 | Current TZD use, 1–4 prescriptions vs nonuse | OR 1.01 (0.34–3.01) | Parallel exposure-stratum result |
| Brauchli 2008 | Current TZD use, ≥5 prescriptions vs nonuse | OR 0.33 (0.16–0.66) | Parallel exposure-stratum result |
| Brauchli 2008 | Past TZD use, 1–4 prescriptions vs nonuse | OR 1.21 (0.41–3.60) | Parallel exposure-stratum result |
| Brauchli 2008 | Past TZD use, ≥5 prescriptions vs nonuse | OR 0.93 (0.38–2.31) | Parallel exposure-stratum result |

**Note:** Effect estimates are presented in their originally reported form and were not transformed or statistically pooled. Supplementary Table S6A presents the selected report-specific estimates used in the evidence synthesis. Supplementary Table S6B presents alternative analyses, sensitivity analyses, secondary or composite outcomes, exposure-timing and prescription-count analyses, adherence analyses, and exploratory subgroup results.

Temporal ascertainment and diagnostic scope are described separately. Study-reported incident outcomes and first-recorded outcomes retain their original ascertainment framework. Confirmed cutaneous psoriasis, broader psoriasis-related coding definitions, composite PsO/PsA outcomes, and outcomes with unconfirmed cutaneous specificity are distinguished and are not treated as interchangeable. Exclusion of previously recorded psoriasis does not by itself establish biological disease onset.

Contemporaneous reasons for the original selection of individual principal results were unavailable. The selection rationale presented during revision is retrospective and cannot establish that the original choices were independent of effect direction, magnitude, statistical significance, or precision. Estimates based on different comparators, outcome definitions, exposure windows, exposure intensities, effect measures, or study designs should not be interpreted as directly comparable.

Mahajan et al. (2026) was retained as supporting evidence within the potentially overlapping TriNetX evidence cluster. Its three pairwise estimates were not interpreted as three independent replications. The GLP-1RA–SGLT2i estimates from Chiang and Mahajan were likewise not assumed to represent independent replication because participant overlap could not be excluded. Related secondary and subgroup results do not automatically inherit the principal result’s ROBINS-I judgment without consideration of their specific design and analysis.

The PDC analyses from Chodick et al. (2015) used statin initiators with PDC <20% as the reference group. Han et al. (2025) reported crude and adjusted estimates that differed in direction. The reported adjusted estimate was retained without inversion; the change in direction does not by itself establish a reporting error. Interpretation also considers the study’s weighting strategy, exposure definition, and outcome scope.

**Abbreviations:** AGI, alpha-glucosidase inhibitor; CI, confidence interval; CISD, chronic inflammatory skin disease; DPP-4i, dipeptidyl peptidase-4 inhibitor; GLP-1RA, glucagon-like peptide-1 receptor agonist; HR, hazard ratio; OR, odds ratio; PDC, proportion of days covered; PsA, psoriatic arthritis; PsO, psoriasis; SGLT2i, sodium-glucose cotransporter-2 inhibitor; TZD, thiazolidinedione.

### 2.1.9 Supplementary Table S7. Selection and interpretation of estimates by medication class

| **Medication class** | **Selected estimate for the evidence synthesis** | **Replacement estimate** | **Supporting evidence** | **Main rationale for evidence selection and handling** |
| --- | --- | --- | --- | --- |
| DPP-4i | Chen WS 2025: selected active-comparator estimate, DPP-4i vs sulfonylurea | No replacement estimate for the same target comparison | Hung 2025; Chiu 2024; Chen YC 2020; Mahajan 2026 | Chen WS 2025 was retained as the representative estimate for DPP-4i versus sulfonylureas within the broader psoriasis-related outcome category. Its outcome definition included PsA codes, so it was not used as a psoriasis-specific estimate. Mahajan 2026 provided additional active-comparator evidence against GLP-1RA and SGLT2i, whereas the Taiwan mixed-comparator studies were retained as supporting evidence. Estimates based on different comparators were interpreted separately. |
| GLP-1RA | Chiang 2026: GLP-1RA vs DPP-4i; displayed in Figure 3 | Lee 2025: alternative report within the likely overlapping TriNetX US evidence cluster | Mahajan 2026; Karacabeyli 2026 composite-outcome estimate; Ching 2025 non-active-comparator estimate | Chiang 2026 and Lee 2025 were interpreted within the same likely overlapping TriNetX US evidence cluster. Their placement in the main and supplementary figures is a presentation choice and does not imply methodological superiority or independent replication. Both estimates were above 1, and considering either leaves the descriptive direction unchanged. Mahajan 2026 was retained as additional evidence with possible population overlap. The Canadian composite-outcome estimate and nonuser comparisons were interpreted separately because they address different outcomes or comparators. |
| SGLT2i | Ma KSK 2024: selected active-comparator estimate, SGLT2i vs DPP-4i | None | Mahajan 2026; Ma SH 2022; Karacabeyli 2026 composite-outcome estimate | Ma KSK 2024 was retained as the representative estimate for SGLT2i versus DPP-4i for study-reported incident psoriasis. The supplementary methods identified TriNetX as the data source and L40 as the outcome code, but did not explicitly establish exclusion of arthropathic psoriasis codes. Restriction to cutaneous psoriasis therefore remained unconfirmed. Mahajan 2026 provided additional active-comparator evidence with similarly unconfirmed cutaneous specificity. Ma SH 2022 used a mixed comparator, whereas Karacabeyli 2026 reported a composite PsO/PsA outcome. These estimates were interpreted separately according to comparator and outcome definition, and possible overlap among TriNetX populations was considered. |
| Metformin | Tseng 2023: representative longitudinal estimate | None | Wu CY 2015 frequent-use estimate; Brauchli 2008 long-term current-use estimate | Comparator groups, exposure definitions, and report designs differed substantially across studies; therefore, the estimates were interpreted separately. |
| TZD | Chen WS 2025: exploratory active-comparator estimate, TZD vs sulfonylurea | None | Wu CY 2015; Brauchli 2008 | Chen WS 2025 provided an active-comparator estimate, but only two outcome events occurred in the TZD group, resulting in substantial imprecision. The estimate was therefore interpreted as exploratory rather than definitive evidence for main interpretation. Brauchli 2008 current and past prescription strata are considered in parallel; its ≥5-prescription result alone is not treated as representative of all TZD exposure. |
| AGI/acarbose | No sufficiently comparable estimate was selected | None | Huang 2021; Brauchli 2008 | The available evidence was based on either a composite PsO/PsA outcome with non-equivalent treatment intensity or sparse acarbose exposure and was therefore interpreted as exploratory. |
| Insulin | No estimate was selected as principal evidence | None | Wu CY 2015 | Only one exposure-frequency report was available, and its estimate was highly susceptible to confounding by indication and concomitant medication use. |
| Statins | Brauchli 2011: principal report-specific statin estimate | None | Han 2025; Chodick 2015 | Brauchli 2011 provides an overall any-statin estimate for its own nonuser contrast. Han contributes both duration strata for a broader coded outcome; Chodick contributes all sex-specific PDC strata among initiators. None of these contrasts is treated as interchangeable. |
| Sulfonylureas | No estimate was selected as principal evidence | None | Brauchli 2008 | Only a supplementary estimate with a confidence interval including 1 based on a nonuser comparison was available. |
| Fibrates | No estimate was selected as principal evidence | None | Brauchli 2011 | The number of exposed cases was small, and the resulting estimate was imprecise. |

**Note:** Evidence roles were assigned separately from ROBINS-I assessment anchors. During revision, we applied the rules described in Methods and presented all relevant strata in parallel where no natural primary result existed. The original selection reasons were not recorded contemporaneously. Revision-stage rules and sensitivity checks improve current transparency but cannot establish that the original selection process was independent of the observed results.

Replacement estimates were retained as alternatives when reports may have used the same or partially overlapping populations. They were not treated as independent confirmation of the corresponding selected estimates. Supporting evidence included mixed-comparator, nonuser, exposure-frequency, exposure-intensity, adherence, and composite-outcome analyses; these analyses were interpreted within their specific clinical and methodological contexts.

The evidence-role terminology in this table describes how estimates were handled in the synthesis. In Supplementary Figure S1, filled squares indicate report–medication coverage only. They do not encode evidence role, effect direction, risk of bias, certainty, or independent replication. Outcome scope is identified separately in the labels and accompanying tables. No pooled summary estimate was calculated.

**Abbreviations:** AGI, alpha-glucosidase inhibitor; DPP-4i, dipeptidyl peptidase-4 inhibitor; GLP-1RA, glucagon-like peptide-1 receptor agonist; PsA, psoriatic arthritis; PsO, psoriasis; SGLT2i, sodium-glucose cotransporter-2 inhibitor; TZD, thiazolidinedione.

### 2.1.10 Supplementary Table S8. Potential overlap among databases and report populations and its handling in the evidence synthesis

| **Evidence cluster** | **Studies** | **Basis for potential overlap** | **Overlap judgment** | **Final handling in this review** |
| --- | --- | --- | --- | --- |
| TriNetX evidence cluster | Chiang 2026; Lee 2025; Mahajan 2026; Ma KSK 2024 | Chiang and Lee used the TriNetX US Collaborative Network, similar T2DM populations, overlapping study periods, and the same GLP-1RA–DPP-4i comparison. Mahajan used the Global Collaborative Network with different eligibility criteria and temporal specifications. The supplementary methods of Ma KSK identified TriNetX data from 92 US healthcare organizations. | Likely overlap between Chiang and Lee. Possible overlap involving Mahajan or Ma KSK and other reports in this cluster. The extent of participant overlap could not be determined. | Chiang was retained as the representative GLP-1RA–DPP-4i report, Lee as an alternative report, and Mahajan as supporting evidence for its reported comparisons. Ma KSK was retained for the SGLT2i–DPP-4i comparison. Reports were interpreted within their specific comparisons and were not treated as independent replications or quantitatively combined. |
| UK GPRD case-control cluster | Brauchli 2008; Brauchli 2011 | Both reports used the same underlying GPRD case-control population, including 36,702 cases and 36,702 controls, with the same study period and matching framework. | Confirmed use of the same underlying study population. | Both reports were retained because they evaluated different medication classes. Their findings were not treated as independent replications. |
| Taiwan NHIRD DPP-4i cluster | Chen WS 2025; Hung 2025; Chiu 2024; Chen YC 2020 | Reports used NHIRD or related subdatabases, included T2DM populations, and evaluated DPP-4i during overlapping study periods. Differences in eligibility criteria and database subsets prevented determination of the extent of overlap. | Possible population overlap within this cluster and with other Taiwan NHIRD reports. | Chen WS was retained as the representative DPP-4i–sulfonylurea estimate for the broader psoriasis-related outcome. Other reports were presented within their own comparator and outcome categories and were not counted as independent replications. |
| Taiwan NHIRD metformin, TZD, insulin, and AGI evidence cluster | Tseng 2023; Wu CY 2015; Huang 2021 | Reports used NHIRD or NHIRD-derived subdatabases and potentially overlapping diabetes populations and study periods, but evaluated different medications or treatment strategies. | Possible population overlap within this cluster and with other Taiwan NHIRD reports. | Findings were presented separately according to medication, comparator, exposure definition, and outcome. Reports were not assumed to represent independent populations and were not quantitatively combined. |
| Taiwan NHIRD SGLT2i evidence | Ma SH 2022 | The report used the same national database system as other Taiwan diabetes studies, although the medication comparison and inclusion period differed. | Possible overlap with other Taiwan NHIRD reports; the extent could not be determined. | Retained as supporting evidence for persistent SGLT2i use versus mixed non-SGLT2i therapy. Population independence from other Taiwan reports was not assumed. |
| All of Us evidence | Ching 2025 | The report used the All of Us database, a distinct named data source from those used by the other included reports. | No overlap identified from the available report-level information. | Presented separately as evidence comparing recorded GLP-1RA exposure with no recorded GLP-1RA exposure. Complete participant independence could not be verified. |
| Canadian Population Data BC evidence | Karacabeyli 2026 | The report used Population Data BC, a distinct named data source from those used by the other included reports. | No overlap identified from the available report-level information. | Retained as supporting active-comparator evidence for the composite psoriasis/psoriatic arthritis outcome. Its different outcome definition was preserved in interpretation. |
| Korean and Israeli statin evidence | Han 2025; Chodick 2015 | Han used the South Korean NHIS-HEALS database, whereas Chodick used Maccabi Healthcare Services data in Israel. These were distinct data sources and geographic populations. | No overlap identified between these reports or with other included reports. | Presented separately because cumulative statin-use and statin-adherence analyses addressed different exposure contrasts. |

Note: Potential population overlap was assessed using the data source, country or region, study period, eligibility criteria, medication comparison, sample characteristics, and other available report-level information. “Confirmed overlap” indicates explicit use of the same underlying study population. “Likely overlap” indicates strong similarities in the data source, population, study period, and comparison without verification of shared participants. “Possible overlap” indicates that shared or nested data sources and compatible populations could have included some of the same participants, but the extent was uncertain. “No overlap identified” indicates that the available information did not reveal overlap and does not prove complete participant independence. These categories describe the evidence supporting overlap rather than its magnitude. The 18 reports were not assumed to represent 18 independent study populations, and an exact number of independent populations could not be established.

### 2.1.11 Supplementary Table S9A. ROBINS-I assessments for the report-level assessment anchor retained from each included report

| Report | ROBINS-I assessment anchor | D1 | D2 | D3 | D4 | D5 | D6 | D7 | Overall |
| --- | --- | --- | --- | --- | --- | --- | --- | --- | --- |
| Chen WS 2025 | DPP-4i vs sulfonylurea; broader psoriasis-related outcome including PsA codes | S | M | L | M | L | M | M | S |
| Chiang 2026 | GLP-1RA vs DPP-4i; study-reported incident psoriasis with unconfirmed cutaneous specificity. | M | M | M | M | M | M | M | M |
| Huang 2021 | AGI plus metformin vs metformin alone; composite PsO/PsA outcome | S | S | M | S | L | M | M | S |
| Tseng 2023 | Metformin vs mixed non-metformin therapy; broad psoriasis-related outcome | S | M | M | M | M | M | M | S |
| Hung 2025 | DPP-4i vs non-DPP-4i therapy; psoriasis-specific outcome | S | S | M | M | L | M | M | S |
| Chiu 2024 | DPP-4i vs mixed non-DPP-4i therapy; broad PsO/PsA-coded outcome | S | S | M | M | L | M | M | S |
| Chen YC 2020 | DPP-4i vs DPP-4i-naïve mixed therapy; broad psoriasis-related outcome | S | S | M | M | L | M | M | S |
| Wu CY 2015 | Frequent metformin use vs nonuse; broad PsO/PsA outcome | S | M | M | M | L | M | M | S |
| Ma KSK 2024 | SGLT2i vs DPP-4i; study-reported incident psoriasis; psoriasis specificity unconfirmed | M | M | M | M | M | M | M | M |
| Ma SH 2022 | Persistent SGLT2i use vs mixed non-SGLT2i therapy; study-reported incident psoriasis; restriction to cutaneous psoriasis unconfirmed | S | M | M | M | L | L | M | S |
| Brauchli 2008 | Current TZD use with ≥5 prescriptions vs nonuse; first-recorded psoriasis; restriction to cutaneous psoriasis unconfirmed | S | M | M | M | M | M | M | S |
| Ching 2025 | Any recorded GLP-1RA exposure vs no recorded GLP-1RA exposure; first-recorded psoriasis; restriction to cutaneous psoriasis unconfirmed | S | S | M | S | M | M | M | S |
| Brauchli 2011 | Any statin use vs no lipid-lowering drug use; first-recorded psoriasis; restriction to cutaneous psoriasis unconfirmed | S | M | M | M | M | M | M | S |
| Han 2025 | Statin use for >365 days vs nonuse; broad PsO/PsA outcome | S | M | M | M | M | M | M | S |
| Chodick 2015 | Women: PDC ≥80% vs PDC <20%; psoriasis; adjusted HR 0.88 (0.79–0.98), original Table 3. Explicit anchor specified retrospectively during revision. | S | S | S | NI | M | L | M | S |
| Karacabeyli 2026 | GLP-1RA vs DPP-4i; composite PsO/PsA outcome | M | L | L | M | L | L | M | M |
| Lee 2025 | GLP-1RA vs DPP-4i; study-reported incident psoriasis; psoriasis specificity unconfirmed | M | M | M | M | M | M | M | M |
| Mahajan 2026 | DPP-4i vs GLP-1RA; study-reported incident psoriasis with unconfirmed cutaneous specificity. | M | M | M | M | M | M | M | M |

**Note:** Each row represents one report-level ROBINS-I assessment anchor retained for traceability. The anchor is not assumed to be the original report’s primary result or a synthesis representative. Related estimates may share domain-level judgments only after checking that the population, exposure timing, comparator, outcome, missing-data structure, and analytical method are sufficiently aligned. Result-specific deviations or additional concerns are summarized in Supplementary Table S9C. Overall judgments were determined according to ROBINS-I guidance and were not calculated by numerical scoring.

Abbreviations: L, low risk of bias; M, moderate risk of bias; S, serious risk of bias; AGI, alpha-glucosidase inhibitor; DPP-4i, dipeptidyl peptidase-4 inhibitor; GLP-1RA, glucagon-like peptide-1 receptor agonist; PDC, proportion of days covered; PsA, psoriatic arthritis; PsO, psoriasis; SGLT2i, sodium-glucose cotransporter-2 inhibitor; TZD, thiazolidinedione. D1, confounding; D2, participant selection; D3, exposure classification; D4, deviations from intended exposure; D5, missing data; D6, outcome measurement; D7, selection of the reported result.

### 2.1.12 Supplementary Table S9B. Distribution of ROBINS-I judgments across domains

| **Domain** | **Low** | **Moderate** | **Serious** | **No information** |
| --- | --- | --- | --- | --- |
| **D1 Confounding** | 0 (0.0%) | 5 (27.8%) | 13 (72.2%) | 0 (0.0%) |
| **D2 Selection** | 1 (5.6%) | 11 (61.1%) | 6 (33.3%) | 0 (0.0%) |
| **D3 Classification** | 2 (11.1%) | 15 (83.3%) | 1 (5.6%) | 0 (0.0%) |
| **D4 Deviations** | 0 (0.0%) | 15 (83.3%) | 2 (11.1%) | 1 (5.6%) |
| **D5 Missing data** | 8 (44.4%) | 10 (55.6%) | 0 (0.0%) | 0 (0.0%) |
| **D6 Outcome measurement** | 3 (16.7%) | 15 (83.3%) | 0 (0.0%) | 0 (0.0%) |
| **D7 Reported result** | 0 (0.0%) | 18 (100.0%) | 0 (0.0%) | 0 (0.0%) |
| **Overall** | 0 (0.0%) | 5 (27.8%) | 13 (72.2%) | 0 (0.0%) |

### 2.1.13 Supplementary Table S9C. Applicability of ROBINS-I assessment-anchor judgments to related estimates and result-specific concerns

| **Report** | **Related estimate(s)** | **Judgments potentially shared with the assessment anchor** | **Result-specific concern(s)** | **Handling in this review** |
| --- | --- | --- | --- | --- |
| Chen WS 2025 | TZD vs sulfonylurea | Participant selection, exposure classification, missing data, outcome measurement, and selective reporting judgments were based on the same overall design framework | Drug-specific residual confounding, residual imbalance, sparse events, and severe imprecision | Retained as exploratory active-comparator evidence; not used as the sole basis for a class-level conclusion |
| Chiang 2026 | GLP-1RA vs insulin, biguanides, sulfonylurea, SGLT2i, or TZD | Time zero, exposure classification, follow-up, outcome measurement, and principal analytical framework | Comparator-specific prescribing and residual confounding | Presented as supporting comparator-specific analyses; the GLP-1RA vs DPP-4i result remained the principal comparison |
| Huang 2021 | Analyses using 30-, 90-, and 180-day treatment-gap windows | Most participant-selection, exposure, outcome, and confounding judgments | Different censoring and exposure-persistence assumptions; sparse events | Presented as sensitivity analyses without separate overall ROBINS-I grades |
| Tseng 2023 | Per-protocol analysis | Population, baseline exposure classification, outcome definition, and most confounding judgments | Deviations from treatment strategy and potentially informative censoring | Presented as a sensitivity analysis sharing the principal assessment, with additional deviation-related concerns noted |
| Hung 2025 | PsA and composite PsO/PsA outcomes | Population, exposure, time zero, confounding, and most analytical judgments | Outcome measurement and directness differed from the psoriasis-specific outcome | Presented as supporting alternative outcomes and not treated as equivalent to psoriasis-specific evidence |
| Chiu 2024 | Monotherapy and additional-adjustment sensitivity analyses | Most design and outcome judgments | Changes in analysis population or adjustment did not resolve the underlying time-zero concern | Presented as sensitivity analyses sharing the principal overall judgment |
| Wu CY 2015 | Frequent TZD, insulin, sulfonylurea, and other drug use vs nonuse | Study population, control selection, outcome ascertainment, missing data, and reporting judgments | Confounding by indication differed by medication class; concomitant treatments complicated exposure interpretation | Drug-specific estimates were presented separately as supporting or exploratory evidence |
| Ma SH 2022 | Kidney-disease subgroup | Population source, exposure framework, outcome definition, and main analysis | Subgroup selection, limited events, and imprecision | Retained as exploratory subgroup evidence |
| Brauchli 2008 | Metformin, sulfonylurea, acarbose, insulin, fibrates, and exposure-timing or prescription-count estimates | Control source, outcome ascertainment, missing data, and most design judgments | Confounding by indication and exposure classification varied across medication classes and exposure strata | Current and past TZD prescription strata are presented in parallel for the TZD comparison; the ≥5-prescription stratum is not treated as representative of all TZD exposure. |
| Brauchli 2011 | Current, recent, and past statin use and prescription-count strata | Population, control source, outcome ascertainment, and most design judgments | Time-related prescribing and exposure-window concerns varied across strata | Presented as exposure-timing and intensity evidence without separate overall ROBINS-I grades |
| Han 2025 | Statin use for 1–365 days vs nonuse | Population, comparator, outcome, and analytical framework | Crude and adjusted directions differed; the cause was not independently verified | Both duration strata are presented and interpreted in parallel; neither is treated as inherently primary because of duration or effect magnitude. |
| Chodick 2015 | Sex-specific PDC categories | Common timing, model, missing-data and ascertainment judgments require a documented check across sex and PDC strata; no automatic sharing is assumed. | PDC may include post-outcome medication information; the magnitude varied across sex and adherence categories | All sex-specific PDC categories are presented in parallel. Separate assessment is required if a stratum changes the relevant population, exposure timing, comparator, outcome, or analytical method. |
| Karacabeyli 2026 | SGLT2i vs DPP-4i | Population, new-user design, time zero, outcome, follow-up, and analytical framework | Comparator-specific residual confounding and differences in treatment discontinuation | Presented as independent supporting active-comparator evidence |
| Lee 2025 | Alternative follow-up or subgroup analyses, where reported | Most design-level judgments | Missing laboratory information and potential overlap with Chiang 2026 remained | Retained as replacement or supporting evidence within the TriNetX cluster |
| Mahajan 2026 | DPP-4i vs SGLT2i; GLP-1RA vs SGLT2i | Participant selection, exposure classification, deviations from intended exposure, missing data, outcome measurement, reporting, and the overall landmark/analytical framework | Comparator-specific residual confounding and differences in the matched treatment populations and follow-up distributions | Presented as supporting active-comparator analyses. The DPP-4i vs GLP-1RA psoriasis estimate remained the principal result selected for the report-level ROBINS-I assessment; no separate overall ROBINS-I grades were assigned to the other pairwise comparisons. |
| Lee 2025 | GLP-1RA vs nonuse; follow-up windows from 3 months to 2, 4, 6, and 8 years | Outcome-coding information may be relevant to both comparisons. Other domain judgments should not be transferred automatically from the GLP-1RA–DPP-4i assessment. | Differences in treatment indication, eligibility, time-zero alignment, covariate balance, and follow-up require comparison-specific evaluation. The eight-year analysis was flagged for a proportional-hazards assumption test with P<0.05. | Reported separately as secondary nonuser-comparator evidence. No separate overall ROBINS-I judgment is currently documented for this comparison, and the active-comparator rating is not assigned to it. |

**Note:** This table does not assign new formal ROBINS-I grades to every secondary, subgroup, sensitivity, or exposure-intensity estimate. It records whether the principal assessment was considered applicable and identifies concerns that were specific to related results. A separate full ROBINS-I assessment should be undertaken if a related estimate becomes central to the main conclusion and differs materially in comparator, eligible population, time zero, outcome definition, or analytical strategy.

### 2.1.14 Supplementary Table S9D. Design-specific considerations for interpreting the ROBINS-I judgments

| **Design** | **Considerations** | **Domains or interpretation** | **Evidence locations** |
| --- | --- | --- | --- |
| Cohort / target-trial emulation | Align eligibility, initiation, baseline covariates, and start of follow-up; inspect persistence requirements and delayed entry | Confounding; selection; classification; deviations; missing data | Chen WS Methods/flow and Table 1; Mahajan supplementary Table 2; S4A–S4B |
| Case-control / nested case-control | Identify source population and control sampling; compare index dates and observation history; establish pre-index prescriptions; preserve matching in analysis | Selection; classification; confounding; outcome measurement | Wu Methods and Table II; Brauchli 2011 Methods and Table II; Han Methods and Table 2 |
| Adherence categories | Separate medication initiation from adherence accumulated during follow-up; assess post-outcome information and healthy-adherer differences | Confounding; selection; classification; deviations | Chodick Methods and Tables 2–3 |
| All designs | Distinguish broad outcome scope from differential misclassification; do not rate serious merely because an interval is wide | Outcome measurement; indirectness and imprecision discussed separately | S5 coding definitions; S12 events; original ROBINS-I framework |
| All designs | Current grades require report-specific evidence; an active comparator or matching alone cannot determine the grade | All seven domains | S3, S9A–S9C; original pre-consensus forms unavailable |

### 2.1.15 Supplementary Table S9E. Source-based clarification of selected ROBINS-I concerns

| **Report and result** | **Domain(s) requiring clarification** | **Source location** | **Documented study feature** | **Implication for assessment** |
| --- | --- | --- | --- | --- |
| Ma SH 2022; persistent SGLT2i use vs mixed non-SGLT2i therapy | D2: selection of participants | Ma SH et al. (2022), Study population and Outcome sections | The index date followed three months of SGLT2i use. Comparator pseudo-index dates were assigned to match diabetes duration. Prior psoriasis was excluded, and follow-up began at index. | Follow-up began after the three-month qualifying exposure period, so that period was not counted as exposed follow-up. The estimate concerns patients eligible at this landmark rather than initiation from first prescription. Participant-selection concerns remain because eligibility required persistent treatment and comparators were defined by never use. For the landmark-based target, D2 was rated moderate in the revision-stage assessment; confounding by treatment indication remains separate. |
| Ma KSK 2024; SGLT2i vs DPP-4i | D1: confounding; D5: missing data; D6: outcome measurement | JAAD online only supplement, Supplementary Methods and Tables S1–S2 | The supplement identifies TriNetX data from 92 US healthcare organizations, extensive matching covariates, prior-outcome exclusion, no imputation, and an L40 outcome definition. | The supplementary methods identified TriNetX data from 92 US healthcare organizations. Residual confounding and incomplete covariate data remained relevant concerns. Unconfirmed restriction to cutaneous psoriasis was considered separately from the severity of outcome-measurement bias. |
| Chodick 2015; women with PDC ≥80% vs <20% | D2: selection; D3: exposure classification; D4: deviations from intended exposure | Original article, printed pages 481–482; Table 3 on page 485; limitations on page 486 | PDC was described as accumulating until disenrollment, death, or study end. Psoriasis was not listed as a stopping event in that definition. Minimum follow-up and high-PDC exclusions were applied. | D3: PDC was defined over follow-up without explicitly stopping accumulation at psoriasis onset, so classification could incorporate post-outcome information; serious concern remains defensible, although the analytical code was unavailable. D4: information on relevant co-interventions and adherence to a defined sustained strategy was insufficient, so D4 was recorded as no information rather than repeating the PDC-timing concern. |
| Chen YC 2020; DPP-4i comparison | D6: outcome measurement | Chen YC et al. (2020), Methods section | Psoriasis used repeated diagnoses and a broad code set. Catastrophic-illness certification described for other autoimmune conditions was not established for psoriasis. | The same repeated-diagnosis algorithm was reported for both groups, but psoriasis-specific validation and differential ascertainment were not established. D6 was rated moderate for the reported post-index psoriasis-related outcome. Unclear prior-psoriasis exclusion limits incident-disease interpretation, and the broad code set limits cutaneous specificity; neither limitation alone was treated as evidence of serious differential outcome measurement. |
| Tseng 2023; metformin comparison | D2: selection and timing | Tseng (2023), Methods, Figure 1, and Table 2 | Follow-up began after the 12-month exposure-assessment period. ITT and per-protocol analyses used different subsequent treatment rules. | Outcome follow-up began after the 12-month exposure-assessment period; this period therefore did not contribute exposed outcome follow-up. Selection and censoring concerns were considered separately for the ITT and per-protocol analyses because their subsequent treatment rules differed. |
| Brauchli 2008; selected TZD comparison | D1: confounding; D6: outcome measurement | Original article, printed page 423, Methods; Table III on page 426 | The study used a general-population nonuser comparison and adjusted recorded factors. Cases had first-recorded psoriasis, prior records, and prior-psoriasis exclusions. A complete cutaneous-only code list was not provided. | Residual confounding by indication remained a concern in the comparison with general-population nonusers. First-recorded ascertainment and unconfirmed cutaneous specificity limited interpretation of the outcome but did not, by themselves, establish serious outcome-measurement bias. |
| Brauchli 2011; any statin use vs no lipid-lowering treatment | D1: confounding; D3: exposure classification; D6: outcome measurement | Original article, printed pages 78–79, Methods and Table II | Exposure was assessed before index using any, current, recent, and past-use definitions. Cases had first-recorded psoriasis and at least three years of prior records. | Residual differences in treatment indication and prescribing remained possible despite adjustment for recorded characteristics. Exposure classification was assessed using this report’s definitions of any, current, recent, and past use before the index date. Restriction of the first-recorded psoriasis outcome to cutaneous disease remained unconfirmed. |
| Karacabeyli 2026; GLP-1RA vs DPP-4i | D1: confounding; D4: deviations and censoring | Karacabeyli et al. (2026), Methods and Statistical analysis sections | The study used a common medication washout and as-treated follow-up. Repeated courses were handled using patient-clustered robust standard errors. | The original analysis accounted for correlation between repeated treatment courses using patient-clustered robust standard errors. Residual confounding and potentially informative treatment discontinuation or switching remained relevant concerns in the as-treated analysis. |
| Han 2025; >365 days of statin use vs nonuse | D1: confounding; reporting interpretation | Han et al. (2025), Methods and Table 2 | Overlap weights were based on psoriasis case-control status. Statin exposure was measured before index. Crude and adjusted estimates differed in direction. | The study used overlap weighting based on psoriasis case-control status, rather than propensity-score matching on statin treatment. The difference in direction between crude and adjusted estimates did not, by itself, establish a reporting error or reversal of the comparison. |

**Note:** This table documents source-based clarifications identified during revision. It does not replace the complete ROBINS-I signalling-question assessment and does not constitute a reconstructed record of the original independent assessments. Final domain and overall judgments require reviewer assessment of the specified result and target effect. Diagnostic scope and statistical imprecision are considered separately from risk-of-bias severity.

### 2.1.16 Supplementary Table S10. Reporting discrepancies, unavailable details, and their handling in this review

| **Report** | **Issue identified in the original report** | **Value or handling adopted in this review** | **Rationale** |
| --- | --- | --- | --- |
| Chen WS 2025 | The post-matching sample sizes for the TZD comparison were reported as 1,066 vs 4,264 in some sections and as 1,064 vs 4,624 elsewhere | The sample sizes were recorded as 1,066 vs 4,264 | These values were consistent with Table 2, Section 3.1 of the Results, and the stated 1:4 matching ratio |
| Chen WS 2025 | The authors described the matched groups as balanced, although several post-matching standardized mean differences remained relatively large | Serious concern regarding residual confounding was retained in the risk-of-bias assessment | The assessment was based on the SMD values reported in the report tables rather than on the authors’ general statement of balance |
| Huang 2021 | The numbers of participants in the AGI and metformin groups were reversed in one section of the main text | The original cohort sizes were recorded as 1,390 vs 47,514, and the matched cohort sizes as 1,299 vs 12,990 | These values were consistent across the abstract, report flow diagram, and Table 1 |
| Hung 2025 | The lower age limit reported in the main text was inconsistent with that shown in the report flow diagram | The population was recorded as adults, with the discrepancy between ≥18 and ≥20 years explicitly noted | No unsupported assumption was made regarding the correct lower age threshold |
| Chen YC 2020 | The main report did not clearly state whether patients with pre-existing psoriasis were excluded at baseline | The baseline psoriasis-free status was classified as unconfirmed | The report was not treated unconditionally as a strict incident-psoriasis cohort |
| Ma KSK 2024 | The main report provided limited database information; the supplementary methods identified TriNetX data from 92 US healthcare organizations. | The data source was recorded as TriNetX, and possible overlap with other included TriNetX reports was considered. | Classification incorporated the available supplementary methods rather than relying only on the main report. |
| Brauchli 2011 | The report used the same underlying cases and controls as Brauchli 2008 | The two reports were classified within the same GPRD evidence cluster | The sample sizes, report period, and matching framework were identical |
| Han 2025 | Original Table 2 reports crude OR 1.37 (1.28–1.47) and overlap-weighted adjusted OR 0.77 (0.73–0.82) for long-term any-statin use. The direction changes after adjustment. | The reported overlap-weighted adjusted OR of 0.77 (95% CI 0.73–0.82) was retained and distinguished from the crude OR and exposure frequencies. No reciprocal transformation was applied, and the change in direction alone was not considered evidence of a reporting error. | The reversal may reflect adjustment/weighting. Its cause cannot be independently verified without the underlying data and code; residual confounding remains relevant. |
| Chodick 2015 | The ordering of the high-adherence estimates for women and men in the abstract was reversed relative to Table 3 | The estimates were recorded as HR 0.88 for women and HR 1.00 for men | The values reported in the main text and Table 3 were used |
| Karacabeyli 2026 | The reported sample sizes represented treatment courses, allowing the same patient to contribute more than one treatment episode | Sample sizes were explicitly labelled as “treatment courses” | The original analysis accounted for within-patient correlation using patient-level clustering |
| Lee 2025 | The supplied eMethod identifies UMLS:ICD10CM:L40 for psoriasis outcome and baseline exclusion, but does not state exclusion of the L40.5 branch. | The psoriasis outcome and baseline exclusion were recorded as UMLS:ICD10CM:L40, as specified in the supplementary eMethod. Restriction to cutaneous psoriasis remained unconfirmed because exclusion of the L40.5 branch was not explicitly documented. | The L40 code is now verified; lack of an explicit L40.5 exclusion leaves cutaneous specificity unconfirmed. |
| Chen WS 2025 | The outcome was described as psoriasis, but the reported diagnostic code set included ICD-9-CM 696.0 for psoriatic arthropathy. | Both the DPP-4i and TZD comparisons were classified as broader psoriasis-related outcomes including PsA codes. | Classification followed the explicitly reported diagnostic codes. A separate effect estimate restricted to cutaneous psoriasis could not be extracted from the published results. |
| Ma KSK 2024 | The supplementary methods specified ICD-10 code L40, but did not explicitly establish exclusion of arthropathic psoriasis codes. | The outcome was classified as study-reported incident psoriasis with unconfirmed restriction to cutaneous psoriasis. | The available coding specification did not establish a cutaneous-only outcome; neither inclusion nor exclusion of arthropathic psoriasis was assumed. |
| Mahajan 2026 | Main report did not anchor the three-year horizon precisely; the supplied supplementary target-trial table specifies days 183–1095 after the first prescription | The outcome follow-up window was recorded as days 183–1095 after the first prescription, according to supplementary Table 2. Restriction of the L40-coded outcome to cutaneous psoriasis remained unconfirmed. | Supplementary Table 2 specified the analytic follow-up window more precisely. Information on baseline exclusions was attributed to its respective source in the main report or supplementary material. |
| Lee 2025 | Supplementary Table 4 prints psoriasis HR 1.19 (1.42–1.25) for 3 months–4 years and 1.18 (1.28–1.23) for 3 months–6 years. Both intervals have reversed bounds and exclude the point estimate. | The two internally inconsistent confidence intervals were not corrected or plotted. The main active-comparator estimate, HR 1.17 (95% CI 1.12–1.22), was retained. | These are source-table inconsistencies, not corrected estimates. Other internally valid sensitivity results are separately identified in S6B. |
| Lee 2025 | The 6-month-lag supplement (Table 2) lists 4,373/4,166 psoriasis events, exceeding the main 90-day-lag counts (4,123/4,021), despite identical displayed matched N of 290,770 per arm. | Event counts were retained with explicit labels identifying the source analysis and lag period. The 6-month-lag and 90-day-lag counts were not assumed to represent nested risk sets, and their discrepancy remained unresolved. | Whether the risk sets were redefined is not resolved by the tabulated information. The 6-month-lag analysis is supplementary only. |

**Note:** Discrepancies were identified by cross-checking the abstracts, main texts, tables, figures, flow diagrams, and supplementary materials of the included reports. Where inconsistencies could be resolved using internally consistent information, the value supported by the greatest number of concordant sources was used. Unresolved discrepancies were explicitly flagged and were not used as the sole basis for the principal interpretation. No unreported source values were invented; the explicitly labelled Ma KSK crude incidence rates in Table S12 were calculated from reported events and person-time.

Abbreviations: AGI, alpha-glucosidase inhibitor; GPRD, General Practice Research Database; HR, hazard ratio; ICD, International Classification of Diseases; OR, odds ratio; PsA, psoriatic arthritis; SMD, standardized mean difference; TZD, thiazolidinedione.

### 2.1.17 Supplementary Table S11. Characteristics of reports evaluating medications and incident or first-recorded psoriasis and broader psoriatic outcomes

| **Report** | **Country/region and data source** | **Study design** | **Study period** | **Analytical sample size** | **Exposure and comparator** | **Outcome definition** | **Follow-up or exposure window** |
| --- | --- | --- | --- | --- | --- | --- | --- |
| **Chen WS 2025** | Taiwan; NHIRD | Time-stratified propensity score–matched active-comparator new-user cohort; target trial emulation | 2006–2015 | DPP-4i: 9,962 vs SU: 39,848; TZD: 1,066 vs SU: 4,264 | DPP-4i or TZD vs sulfonylurea | Broader psoriasis-related outcome including PsA codes, defined by at least two visits with ICD-9-CM codes 696.0, 696.1, 696.5, or 696.8 within 365 days after the index date. | 365 days from the index date |
| **Chiang 2026** | United States; TriNetX US Collaborative Network | Active-comparator new-user target trial emulation; ITT; 1:1 PSM | 2018–2022 | 169,630 vs 169,630 | GLP-1RA vs DPP-4i | Study-reported incident psoriasis identified using ICD-10-CM code L40; restriction to cutaneous psoriasis unconfirmed. | 90-day lag; up to 4 years |
| **Huang 2021** | Taiwan; LHDB/NHIRD | Retrospective matched cohort study | 1999–2013 | 1,299 vs 12,990 | AGI plus metformin vs metformin alone | Incident psoriatic disease, a composite of PsO or PsA | Treatment-gap windows of 30, 90, and 180 days |
| **Tseng 2023** | Taiwan; NHIRD | Retrospective landmark cohort with a 12-month exposure-assessment window; IPTW | 1999–2009; follow-up through 2011 | 234,585 vs 125,921 | Metformin vs mixed non-metformin therapy | Incident psoriasis-related diagnosis, ICD-9-CM 696 | Follow-up after the 12-month landmark period |
| **Hung 2025** | Taiwan; LHID/NHIRD | 1:4 matched retrospective cohort study | 2009–2021 | 15,773 vs 63,092 | DPP-4i vs non-DPP-4i oral glucose-lowering therapy | PsO, PsA, and composite PsD reported separately | Median follow-up, 7.15 vs 7.04 years |
| **Chiu 2024** | Taiwan; NHIRD | Propensity score–adjusted retrospective cohort study | 2009–2019 | 1,312,521 vs 2,700,856 | DPP-4i vs mixed non-DPP-4i therapy | Broad psoriasis-related outcome including PsA codes | Follow-up through 2019 |
| **Chen YC 2020** | Taiwan; NHIRD diabetes subdatabase | Prescription-time distribution matching plus 1:1 PSM cohort | 2009–2013 | 387,099 vs 387,099 | DPP-4i vs DPP-4i-naïve mixed therapy | Broad psoriasis-related diagnosis after the index date; baseline exclusion not clearly specified in the main report | Follow-up through 2013 |
| **Wu CY** | Taiwan; NHIRD | Nested case-control study within a nationwide diabetes cohort | 1997–2011 | 9,243 cases; 36,972 controls | Frequent metformin, TZD, or insulin use vs corresponding nonuse | First-recorded broader/composite PsO/PsA outcome | 3-year exposure window before the index date |
| **2015** |  |  |  |  |  |  |  |
| **Ma KSK 2024** | United States; TriNetX electronic health records from 92 healthcare organizations. | PSM active-comparator treatment-initiator cohort; ITT | 2014–2024 | 550,195 vs 550,195 | SGLT2i vs DPP-4i | Study-reported incident psoriasis identified using ICD-10 code L40; exclusion of arthropathic psoriasis codes was not explicitly documented. | Up to 5 years |
| **Ma SH 2022** | Taiwan; NHIRD-MOHW | Matched cohort of persistent medication users; competing-risk analysis | 2007–2018 | 103,745 vs 207,490 | Persistent SGLT2i use vs mixed non-SGLT2i therapy | Study-reported incident psoriasis: ICD-9-CM 696.1 or ICD-10 L40; at least three specialist outpatient diagnoses or one inpatient diagnosis. Restriction to cutaneous psoriasis unconfirmed. | Mean follow-up, 1.19 vs 1.52 years |
| **Brauchli 2008** | United Kingdom; GPRD | 1:1 matched case-control study | 1994–2005 | 36,702 cases; 36,702 controls | TZD, metformin, SU, or acarbose vs corresponding nonuse | First-recorded psoriasis identified using OXMIS/Read codes; restriction to cutaneous psoriasis unconfirmed. | ≥3 years of database history before the index date |
| **Ching 2025** | United States; All of Us | Retrospective cohort analysis using All of Us EHR data; multivariable logistic regression | Database queried May 2018–October 2023; historical EHR data have no finite start date | 73,225 in psoriasis analysis: 14,178 exposed / 59,047 unexposed; 74,910 before outcome-specific exclusions | Any prior GLP-1RA exposure vs no GLP-1RA exposure | First-recorded psoriasis after T2DM and, for exposed patients, after first GLP-1RA exposure | No uniform person-time model reported |
| **Brauchli 2011** | United Kingdom; GPRD | 1:1 matched case-control study | 1994–2005 | 36,702 cases; 36,702 controls | Statin or fibrate use vs no lipid-lowering drug use | First-recorded psoriasis identified using OXMIS/Read codes; restriction to cutaneous psoriasis unconfirmed. | ≥3 years of database history before the index date |
| **Han 2025** | South Korea; NHIS-HEALS | Matched case-control study nested within a health-screening cohort | 2002–2019 | 8,285 cases; 33,140 controls | Cumulative statin use during the 2 years before the index date vs nonuse | Broad psoriasis-related outcome including PsA | 2-year exposure window |
| **Chodick 2015** | Israel; Maccabi Healthcare Services | Retrospective cohort of new statin users | 1998–2009; follow-up through 2010 | 205,820 | Different PDC categories; PDC <20% as the reference | Incident psoriasis recorded by a specialist or during hospitalization | Median follow-up, 5.74 years |
| **Karacabeyli 2026** | Canada; Population Data BC | Active-comparator new-user cohort; generalized propensity score matching weights | 2014–2022; follow-up through 2023 | GLP-1RA: 49,514 treatment episodes; SGLT2i: 101,925; DPP-4i: 77,861 | GLP-1RA or SGLT2i vs DPP-4i | Incident psoriatic disease, a composite of PsO or PsA | Mean follow-up, 1.27, 1.64, and 1.82 years, respectively |
| **Lee 2025** | United States; TriNetX US Collaborative Network | Active-comparator new-user ITT cohort; 1:1 PSM | 2015–2022; observation through 2025 | 290,770 vs 290,770 | GLP-1RA vs DPP-4i | Study-reported incident psoriasis from day 90; eMethod: UMLS:ICD10CM:L40; cutaneous restriction unconfirmed because no L40.5 exclusion is specified. | 90-day lag; up to 8 years |
| **Mahajan 2026** | Multinational; TriNetX Global Collaborative Network | Three active-comparator new-user emulated target trials; ITT; 1:1 PSM | 2016–2023; follow-up through 25 February 2025 | DPP-4i vs GLP-1RA: 118,419 vs 118,419; DPP-4i vs SGLT2i: 102,810 vs 102,810; GLP-1RA vs SGLT2i: 105,869 vs 105,869 | New initiation of DPP-4i, GLP-1RA, or SGLT2i monotherapy in pairwise active-drug comparisons | Study-reported incident psoriasis identified using ICD-10-CM code L40. Psoriasis and PsA were reported separately, but mutual exclusivity of the coding definitions was not established. | Follow-up began at the 6-month landmark. Supplementary Table 2 specifies outcome follow-up from day 183 after the first prescription until outcome occurrence, database withdrawal or death, or day 1095 after that prescription, whichever occurred first. Thus, the three-year endpoint was anchored to the index prescription, not three additional years after the landmark. Follow-up ended at outcome occurrence, death, loss to follow-up or database withdrawal, or study end, whichever occurred first. |

**Abbreviations:** AGI, alpha-glucosidase inhibitor; DPP-4i, dipeptidyl peptidase-4 inhibitor; EHR, electronic health record; GLP-1RA, glucagon-like peptide-1 receptor agonist; GPRD, General Practice Research Database; ICD, International Classification of Diseases; IPTW, inverse probability of treatment weighting; ITT, intention-to-treat; LHDB, Longitudinal Health Insurance Database; LHID, Longitudinal Health Insurance Database; MOHW, Ministry of Health and Welfare; NHIRD, National Health Insurance Research Database; NHIS-HEALS, National Health Insurance Service–Health Screening Cohort; PDC, proportion of days covered; PsA, psoriatic arthritis; PsD, psoriatic disease; PSM, propensity score matching; PsO, psoriasis; SGLT2i, sodium-glucose cotransporter-2 inhibitor; SU, sulfonylurea; TZD, thiazolidinedione.

### 2.1.18 Supplementary Table S12. Events and absolute outcome measures

| **Report** | **Comparison and outcome** | **Population and events** | **Time at risk** | **Absolute measure** | **Source and qualification** |
| --- | --- | --- | --- | --- | --- |
| Chen WS 2025 | DPP-4i vs SU | N 9,962 / 39,848 | 365-day window; PY 9,019.80 / 38,115.25 | IR 188.47 / 467.00 per 100,000 PY | Table 3A; sample sizes Table 1 |
|  | Broader outcome including PsA | Events 17 / 178 |  |  |  |
| Chen WS 2025 | TZD vs SU | N 1,066 / 4,264 (table-based) | 365-day window; PY 1,014.16 / 4,149.68 | IR 197.21 / 457.87 per 100,000 PY | Tables 2 and 3B; narrative sample counts differ; no derived risk |
|  | Broader outcome including PsA | Events 2 / 19 |  |  |  |
| Chiang 2026 | GLP-1RA vs DPP-4i | N 169,630 / 169,630 | 90-day lag; up to 4 years | Table 2 risk 0.941% / 0.819%; not relabelled as adjusted 4-year cumulative incidence | Main Table 2; supplementary Method S1 |
|  | Study-reported psoriasis; specificity unconfirmed | Events 1,597 / 1,390 |  |  |  |
| Huang 2021 | AGI + metformin vs metformin | N 1,299 / 12,990 | 30-day treatment-gap analysis; PY 859 / 7,727 | IR 349.25 / 38.83 per 100,000 PY; reported proportions 0.23% / 0.02% | Table 2, after 1:10 matching; Table 3 HR 8.77 |
|  | Composite PsO/PsA | Events 3 / 3 |  |  |  |
| Tseng 2023 | Metformin vs non-metformin; ITT | N 234,585 / 125,921 | PY 1,417,780.27 / 909,121.97 | IR 178.87 / 200.19 per 100,000 PY | Table 2, psoriasis ITT rows |
|  | Broader ICD-9 696 outcome | Events 2,536 / 1,820 |  |  |  |
| Hung 2025 | DPP-4i vs other oral therapy | N 15,773 / 63,092 | PY 112,776.35 / 448,512.26 | IR 37.24 / 63.77 per 100,000 PY | Table V; Methods coding definition |
|  | Cutaneous PsO codes | Events 42 / 286 |  |  |  |
| Hung 2025 | DPP-4i vs other oral therapy | N 15,773 / 63,092 | PY 112,776.35 / 448,512.26 | IR 45.22 / 76.25 per 100,000 PY | Table V; not substituted for PsO events |
|  | Composite PsO/PsA (supporting) | Events 51 / 342 |  |  |  |
| Chiu 2024 | DPP-4i vs non-DPP-4i | N 1,312,521 / 2,700,856 | PY 5,550,364.1 / 13,406,331.0 | IR 1.834 / 2.059 per 1,000 PY; proportions 0.78% / 1.02% | Table 1, psoriasis row |
|  | Broader coded outcome | Events 10,177 / 27,598 |  |  |  |
| Chen YC 2020 | DPP-4i vs DPP-4i-naive | N 387,099 / 387,099 | Follow-up through 2013; psoriasis-specific PY not reported in Table 2 | Reported proportions 0.27% / 0.47%; not fixed-horizon adjusted risks | Table 2 |
|  | Broader post-index diagnosis | Events 1,034 / 1,809 |  |  |  |
| Wu CY 2015 | Frequent metformin vs nonuse | 9,243 cases; 36,972 controls | Prescriptions in 3 years before index | Not estimable from case-control sampling; exposure counts are not medication-group incidence | Table II |
|  | First-recorded broader PsO/PsA | Exposed cases/controls 4,587 / 18,406; nonusers 2,099 / 8,580 |  |  |  |
| Ma KSK 2024 | SGLT2i vs DPP-4i | Matched N 550,195 / 550,195 | Outcome-specific PY 1,313,282 / 1,732,071; up to 5 years | Derived crude IR 286.84 / 259.52 per 100,000 PY (events/PY); not adjusted risks | Table I; supplied online supplement |
|  | L40 outcome; specificity unconfirmed | Events 3,767 / 4,495 |  |  |  |
| Ma SH 2022 | Persistent SGLT2i vs non-SGLT2i | N 103,745 / 207,490 | Mean follow-up 1.19 / 1.52 years | Reported rounded proportions 0.1% / 0.1%; not equal-horizon cumulative incidence | Table 1 |
|  | Study-reported psoriasis; specificity unconfirmed | Events 79 / 167 |  |  |  |
| Brauchli 2008 |  | 36,702 cases / 36,702 controls | Case-control sampling; 1994–2005 | Not estimable from case-control sampling | Full text Table III, p.426; adjusted OR 0.33 (0.16–0.66). Exposure counts are not incidence denominators |
|  | Current TZD use with ≥5 prescriptions vs target-drug nonuse; first-recorded psoriasis; restriction to cutaneous psoriasis unconfirmed | Current TZD ≥5 prescriptions: 12 cases / 36 controls |  |  |  |
|  |  | Target-drug nonuse: 36,662 / 36,642 |  |  |  |
| Ching 2025 | GLP-1RA exposure vs no exposure | Psoriasis-analysis N 73,225: exposed 14,178 / unexposed 59,047 | No uniform exposure-based follow-up horizon or person-time denominator reported | No fixed-horizon incidence risk inferred from these diagnosis counts; adjusted OR 0.41 (0.35–0.48) | Full text Table I, p.98; total identified T2DM N 74,910 differs from the outcome-specific analysis cohort |
|  | First-recorded psoriasis; specificity unconfirmed | Psoriasis diagnoses 182 / 1,419 |  |  |  |
| Brauchli 2011 | Any statin use vs no lipid-lowering medication use; first-recorded psoriasis; restriction to cutaneous psoriasis unconfirmed | 36,702 cases; 36,702 controls | Case-control sampling; 1994–2005 | Not estimable from case-control sampling; counts shown for a reported supporting exposure stratum | Table II; overall any-statin OR described in Results |
|  | Supporting exposure stratum: current statin use with ≥20 prescriptions vs no lipid-lowering medication use; first-recorded psoriasis; restriction to cutaneous psoriasis unconfirmed | Current ≥20 prescriptions: 385 cases / 331 controls; nonusers 34,681 / 34,922 |  |  |  |
| Han 2025 | Any statin >365 days vs nonuse | 8,285 cases; 33,140 controls | Exposure during 2 years before index | Not estimable from case-control sampling; frequencies are unweighted | Table 2 |
|  | Broader PsO/PsA-coded outcome | Exposed cases/controls 1,373 / 4,434; nonusers 5,623 / 24,888 |  |  |  |
| Chodick 2015 | Women, PDC ≥80% vs <20% | 111,351 women overall; category denominators not given in Table 3 | Median 5.74 years for whole cohort | Sex-by-PDC absolute rate not reported in Table 3; overall/category rates below | Table 3; HR 0.88 (0.79–0.98) |
|  | Specialist/hospital-recorded PsO | Events 882 / 714 |  |  |  |
| Chodick 2015 | Both sexes, PDC ≥80% vs <20% | N 57,947 / 57,401 | PY 377,196 / 317,409 | IR 4.82 / 4.45 per 1,000 PY; do not pair these mixed-sex rates with women-only adjusted HR | Table 2; whole cohort 5,615 events / 205,820; IR 4.40/1,000 PY |
|  | Supporting crude category comparison | Events 1,818 / 1,414 |  |  |  |
| Karacabeyli 2026 | GLP-1RA / SGLT2i / DPP-4i | 49,514 / 101,925 / 77,861 treatment courses | Mean follow-up 1.27 / 1.64 / 1.82 years | Crude IR 12.5 / 8.4 / 5.6; matching-weighted IR 11.1 / 9.8 / 8.8 per 10,000 PY | Table 3; courses are not necessarily unique individuals |
|  | Composite PsO/PsA | Events 79 / 142 / 80 |  |  |  |
| Lee 2025 | GLP-1RA vs DPP-4i | Matched N 290,770 / 290,770 | 90-day lag; maximum 8 years | Reported cumulative incidence 2.77% / 2.39%; not event count divided by matched N | Table 2 |
|  | Study-reported psoriasis; specificity unconfirmed | Events 4,123 / 4,021 |  |  |  |
| Mahajan 2026 | DPP-4i vs GLP-1RA | Matched N 118,419 / 118,419 | Day 183 through day 1095 from first prescription | Adjusted absolute psoriasis risk not reported in Table 2; counts not converted to three-year risks | Main Table 2; supplementary Table 2; one report, three comparisons |
|  | L40 outcome; specificity unconfirmed | Events 756 / 851 |  |  |  |
| Mahajan 2026 | DPP-4i vs SGLT2i | Matched N 102,810 / 102,810 | Day 183 through day 1095 from first prescription | Adjusted absolute psoriasis risk not reported in Table 2; counts not converted to three-year risks | Main Table 2; supplementary Table 2; one report, three comparisons |
|  | L40 outcome; specificity unconfirmed | Events 519 / 621 |  |  |  |
| Mahajan 2026 | GLP-1RA vs SGLT2i | Matched N 105,869 / 105,869 | Day 183 through day 1095 from first prescription | Adjusted absolute psoriasis risk not reported in Table 2; counts not converted to three-year risks | Main Table 2; supplementary Table 2; one report, three comparisons |
|  | L40 outcome; specificity unconfirmed | Events 777 / 632 |  |  |  |
| Brauchli 2008 | Current metformin use with ≥15 prescriptions vs target-drug nonuse; first-recorded psoriasis; restriction to cutaneous psoriasis unconfirmed | 36,702 cases / 36,702 controls | Last prescription within 89 days before index; case-control sampling | Not an incidence-risk denominator | Full text Table III; adjusted OR 0.77 (0.62–0.96) |
|  |  | Exposed cases/controls 280 / 283; nonusers 36,127 / 36,115 |  |  |  |
| Brauchli 2008 | Current sulfonylurea use with ≥15 prescriptions vs target-drug nonuse; first-recorded psoriasis; restriction to cutaneous psoriasis unconfirmed | 36,702 cases / 36,702 controls | Last prescription within 89 days before index; case-control sampling | Not an incidence-risk denominator | Full text Table III; adjusted OR 1.07 (0.88–1.31) |
|  |  | Exposed cases/controls 311 / 296; nonusers 36,064 / 36,090 |  |  |  |
| Brauchli 2008 | Current acarbose use with ≥5 prescriptions vs target-drug nonuse; first-recorded psoriasis; restriction to cutaneous psoriasis unconfirmed | 36,702 cases / 36,702 controls | Last prescription within 89 days before index; case-control sampling | Not an incidence-risk denominator | Full text Table III; adjusted OR 1.33 (0.56–3.16) |
|  |  | Exposed cases/controls 14 / 10; nonusers 36,654 / 36,645 |  |  |  |

### 2.1.19 Supplementary Table S13. Comparison-specific limitations and interpretation of the included evidence

| **Medication comparison** | **Outcome scope** | **Main limitations** | **Interpretation** |
| --- | --- | --- | --- |
| DPP-4i vs SU | Broader psoriasis-related outcome including PsA codes | The representative Taiwan report included 17 and 178 events in the respective groups. Residual confounding, outcome breadth, and uncertainty about independence from other Taiwan reports limit interpretation. | The lower estimate is hypothesis-generating. Estimates from heterogeneous non-DPP-4i comparator groups address different questions and do not provide confirmed independent replication. |
| GLP-1RA vs DPP-4i | Study-reported psoriasis; restriction to cutaneous psoriasis unconfirmed | Residual confounding and likely or possible overlap among TriNetX reports limit independence. Relatively narrow confidence intervals do not resolve these concerns. | Estimates above 1 do not establish a causal increase in cutaneous psoriasis. Findings remain specific to the comparison and outcome definition. |
| DPP-4i vs SGLT2i | Study-reported psoriasis; restriction to cutaneous psoriasis unconfirmed | Mahajan reported an HR of 0.81 (95% CI 0.70–0.91). Residual confounding and possible overlap with other TriNetX reports remain relevant. | The lower DPP-4i estimate is comparison-specific. The original direction is retained; this row and the reverse-direction SGLT2i–DPP-4i row concern the same medication pair, not independent clinical questions. |
| GLP-1RA vs SGLT2i | Study-reported psoriasis; restriction to cutaneous psoriasis unconfirmed | Chiang reported an HR of 0.99 (95% CI 0.91–1.07), and Mahajan reported an HR of 1.02 (95% CI 0.91–1.15). Residual confounding, differences in eligibility and follow-up, and possible TriNetX population overlap limit comparability and independence. | Both point estimates were close to 1. The confidence intervals allow modest differences in either direction and do not establish equivalence. The reports are not confirmed independent replications. |
| GLP-1RA vs nonuse | Ching: first-recorded psoriasis; Lee: study-reported incident psoriasis. Restriction to cutaneous psoriasis unconfirmed. | Ching did not clearly establish a common time zero and comparable follow-up for the nonuser comparison. Lee reported secondary nonuser comparisons across several follow-up windows, with a proportional-hazards concern at the eight-year horizon. No separate overall ROBINS-I judgment is currently documented for Lee’s nonuser comparison; the rating for Lee’s active-comparator analysis was not transferred to it. This limits the completeness of the comparison-specific risk-of-bias appraisal. | The different directions should not be interpreted as directly contradictory estimates of the same treatment effect. Neither comparison establishes protection or a causal increase. |
| SGLT2i vs DPP-4i | Study-reported psoriasis; restriction to cutaneous psoriasis unconfirmed | Ma KSK reported a modestly elevated estimate. The supplementary methods document extensive matching, but incomplete covariate data, unmeasured confounding, and possible TriNetX overlap remain relevant. | The association is hypothesis-generating and does not establish a stable medication-class effect. |
| GLP-1RA or SGLT2i vs DPP-4i | Composite PsO/PsA outcome | The Canadian report provides separate composite-outcome evidence. Residual confounding, potentially informative treatment discontinuation or switching, and wide confidence intervals limit interpretation. | These estimates do not isolate cutaneous psoriasis and should not be treated as interchangeable with cutaneous-outcome estimates. |
| Persistent SGLT2i use vs mixed non-SGLT2i therapy | Study-reported psoriasis; restriction to cutaneous psoriasis unconfirmed | In Ma SH, follow-up began after the qualifying three-month exposure period. Selection of persistent users, the never-user comparator, residual confounding, and imprecision require consideration. The qualifying period should not be assumed to have contributed exposed follow-up time. | The findings do not establish an increase, decrease, or absence of association. Bias judgments must reflect the actual timing of eligibility and follow-up. |
| Metformin vs mixed therapy | Broad ICD-9 696 outcome | Residual confounding and a heterogeneous comparator limit interpretation. Follow-up in Tseng began after the exposure-assessment period. Other metformin analyses used different comparators and exposure definitions. | The lower estimate does not establish prevention of cutaneous psoriasis. Different metformin contrasts should not be treated as estimates of one common effect. |
| TZD vs SU | Broader psoriasis-related outcome including PsA codes | The comparison had only two exposed events and a very wide confidence interval, in addition to residual-confounding concerns. | The estimate is too imprecise for a stable directional inference. |
| AGI plus metformin vs metformin alone | Composite PsO/PsA outcome | The groups differed in treatment intensity. There were three events in each matched group. Eligibility based on cumulative treatment and its relation to the index date also require consideration. | The large relative estimate is unstable and does not establish a medication-class effect. |
| Statin use vs nonuse | Brauchli: first-recorded psoriasis, with unconfirmed cutaneous specificity. Han: broader PsO/PsA-coded outcome. | Exposure definitions and comparator populations differed. Residual confounding remains relevant. In Han, crude and adjusted estimates differed in direction; this alone does not establish a reporting error. | No consistent association was demonstrated across the distinct contrasts. This does not prove absence of an association. |
| High vs low statin adherence | Specialist- or hospital-recorded psoriasis | Chodick classified PDC over follow-up, with no psoriasis stopping event stated in the PDC definition. This raises concern about possible use of post-outcome information, although the implemented code was unavailable. Selection and sex-specific analyses also require consideration. | The comparison concerns adherence among statin initiators, not statin initiation versus nonuse. It does not establish a stable exposure–response pattern. |
| TZD use vs nonuse, by exposure frequency and timing | Wu: broad PsO/PsA outcome. Brauchli 2008: first-recorded psoriasis with unconfirmed cutaneous specificity. | Nonuser comparators, residual confounding by indication, different exposure definitions, and sparse exposure counts in several strata limit interpretation. Brauchli 2008 reported current-use ORs of 1.01 (95% CI 0.34–3.01) and 0.33 (0.16–0.66) for 1–4 and ≥5 prescriptions, respectively; the corresponding past-use ORs were 1.21 (0.41–3.60) and 0.93 (0.38–2.31). | The markedly lower estimate in Brauchli 2008 was confined to one current-use stratum. The findings do not establish a consistent exposure pattern or a preventive effect shared across TZDs. Wu’s frequent-use comparison addresses a different exposure definition and outcome scope. |

**Note:** These are comparison-specific interpretive appraisals, not formal GRADE ratings. Effect estimates are retained in their originally reported direction and are not quantitatively pooled. Related comparisons and potentially overlapping reports should not be counted as independent replications. The assessment-anchor ROBINS-I judgment for a report should not automatically be applied to all secondary comparisons from that report. DPP-4i, dipeptidyl peptidase-4 inhibitor; GLP-1RA, glucagon-like peptide-1 receptor agonist; SGLT2i, sodium-glucose cotransporter-2 inhibitor; SU, sulfonylurea; TZD, thiazolidinedione; AGI, alpha-glucosidase inhibitor; PsO, psoriasis; PsA, psoriatic arthritis; PDC, proportion of days covered; HR, hazard ratio; CI, confidence interval.

### 2.1.20 Supplementary Table S14. Changes and clarifications relative to the registered protocol and initial manuscript

| **Item** | **Registered protocol or initial manuscript** | **Revised reporting** | **Nature and implications of the change** |
| --- | --- | --- | --- |
| Outcome terminology | The registered title referred to incident psoriasis, whereas the protocol text already included incident or first-recorded psoriasis. | Incident and first-recorded psoriasis are explicitly distinguished in the manuscript. Temporal ascertainment and diagnostic scope are described separately. | This clarifies terminology already present in the protocol text; it does not introduce first-recorded psoriasis as a newly eligible outcome. |
| Updated literature search | The registered protocol described searches through 1 July 2026. The initial manuscript included 17 reports. | A revision-stage search was conducted on 11 September 2026, and one additional eligible report was included, bringing the total to 18. | This updates the evidence base. The update is reported separately from the initial search. The scope of cross-deduplication against the initial-search database could not be confirmed. |
| Outcome-selection hierarchy | The registered protocol prioritized the original study’s primary outcome when multiple definitions were available, followed by a psoriasis-specific, clinically restricted, or validated definition. | The revised manuscript prioritizes separately reported psoriasis outcomes relevant to the review question and distinguishes these from composite psoriasis/psoriatic arthritis outcomes. | The revised hierarchy differs from the wording of the registered protocol. It is disclosed as a revision-stage clarification and is not presented as a prospectively specified decision. The selected outcomes are identified in the extraction tables. |
| Selection of ROBINS-I assessment anchors and synthesis results | Contemporaneous reasons for the original selection of individual principal results were unavailable. | The revision distinguishes assessment anchors from synthesis representatives, presents the Chodick, Brauchli 2008 and Han strata in parallel, and adds a qualitative sensitivity assessment. | These rules and checks were introduced during revision. They do not reconstruct or retrospectively validate the original selection process. |
| Outcome classification | Some manuscript and supplementary labels did not consistently distinguish temporal ascertainment from diagnostic scope. | First-recorded outcomes, confirmed cutaneous outcomes, broader coding definitions, composite outcomes, and outcomes with unconfirmed cutaneous specificity are explicitly distinguished. | This improves consistency of interpretation without changing an effect estimate solely because its outcome label is clarified. |
| Additional evidence reporting | Reporting of event counts, person-time, and comparison-specific limitations was less complete in the initial manuscript. | Supplementary Tables S12 and S13 provide additional absolute-data context and comparison-specific interpretation. | These additions improve reporting transparency. The comparison-specific appraisals are not formal GRADE ratings. |
| Funding statement | The registered protocol stated “Support—None,” whereas the manuscript acknowledged Grant No. ZHY2025-020. | The authors confirmed that Grant No. ZHY2025-020 supported this review. The funding acknowledgement is retained in the manuscript. | Grant No. ZHY2025-020 supported this review. The INPLASY registration recorded “Support—None” at the time of registration. Support for this review under Grant No. ZHY2025-020 was obtained subsequently. The manuscript funding statement reflects this later support. |

## 2.2 Supplementary Figures

### 2.2.1 Supplementary Figure S1. Drug-by-report coverage matrix across medication classes.

Figure S1. Medication coverage and outcome scope across the included reports.

Filled squares identify medication classes or exposure strategies mapped as targets of analysis in each report; comparator-only medications are not necessarily marked. Blank cells indicate that no target estimate was mapped for that medication–report combination, rather than absence of medication use in the study population. The right-hand column summarizes the outcome scope of each report. Squares do not indicate effect direction, effect magnitude, risk of bias, or independent replication. Asterisks indicate that restriction of the outcome to cutaneous psoriasis could not be confirmed.

Abbreviations: AGI, alpha-glucosidase inhibitor; DPP-4i, dipeptidyl peptidase-4 inhibitor; GLP-1RA, glucagon-like peptide-1 receptor agonist; PsA, psoriatic arthritis; PsO, psoriasis; SGLT2i, sodium–glucose cotransporter-2 inhibitor; TZD, thiazolidinedione.

### 2.2.2 Supplementary Figure S2. Drug-nonuser and exposure-intensity comparisons for glucose-lowering medications

Supplementary Figure S2. Medication nonuser and exposure-intensity comparisons for glucose-lowering medications. Open circles indicate reported adjusted odds ratios (ORs), and horizontal lines indicate 95% confidence intervals. The forest axis is logarithmic; the vertical dashed line indicates the null value of 1. All four Brauchli 2008 TZD exposure strata documented in Supplementary Table S6 are displayed together: current use with 1–4 or ≥5 prescriptions and past use with 1–4 or ≥5 prescriptions. The remaining rows present selected medication and exposure-intensity comparisons from Supplementary Table S2; the figure is not an exhaustive display of every exposure stratum for every medication. Asterisks identify Brauchli 2008 estimates for first-recorded psoriasis, for which restriction to cutaneous psoriasis could not be confirmed. Wu CY 2015 used a composite PsO/PsA outcome identified with a broader diagnostic code set. First-recorded diagnoses do not independently establish biological disease onset. Outcome definitions, medication comparisons, exposure windows, and study designs differ and should not be treated as interchangeable. Multiple estimates within a report are not independent replications. Marker sizes do not represent statistical weights, and no pooled estimate was calculated. Confidence intervals including 1 do not establish absence of an association. Rx, prescriptions; TZD, thiazolidinedione; PsO, psoriasis; PsA, psoriatic arthritis.

### 2.2.3 Supplementary Figure S3. Statin exposure timing, cumulative use, and adherence in relation to psoriasis risk

|  |  |
| --- | --- |
| A | B |

Supplementary Figure S3. Statin exposure timing, cumulative use, and adherence in relation to psoriasis-related outcomes.(A) Reported adjusted odds ratios (ORs) and 95% confidence intervals (CIs) for statin use according to exposure timing, prescription count, and cumulative duration. Brauchli (2011) used a matched case-control design with first-recorded psoriasis as the outcome and no lipid-lowering drug use as the reference. The asterisk indicates that restriction of the outcome to cutaneous psoriasis could not be confirmed. Han (2025) used a nested case-control design with a broader psoriasis/psoriatic arthritis-coded outcome and statin nonuse as the reference. Han’s crude and adjusted estimates differ in direction; this difference alone does not establish a reporting error. Open circles indicate ORs. (B) Sex-specific hazard ratios (HRs) and 95% CIs for incident psoriasis across proportion-of-days-covered (PDC) categories among statin initiators, with PDC <20% as the reference. Filled squares represent women, and open circles represent men. PDC was assessed over the observed follow-up period rather than within a fixed baseline exposure window. In both panels, horizontal lines indicate 95% CIs, effect estimates are plotted on logarithmic scales, and vertical dashed lines indicate the null value of 1. Marker sizes do not represent statistical weights, and no pooled estimates were calculated. Comparators, outcome definitions, effect measures, study designs, and temporal frameworks differ across studies; estimates are therefore not directly interchangeable. See Table S4A for exposure timing and limitations. Rx, prescriptions; PsO, psoriasis; PsA, psoriatic arthritis.

### 2.2.4 Supplementary Figure S4. Additional, sensitivity, and exploratory effect estimates across medication comparisons and outcome definitions.

|  |  |  |
| --- | --- | --- |
| A | B | C |

Supplementary Figure S4. Additional, sensitivity, and exploratory estimates across medication comparisons and outcome definitions. Panel A presents alternative and supporting active-comparator estimates, including within-report comparisons, potentially overlapping reports, and composite-outcome evidence. Panel B presents alternative analyses, secondary or composite outcomes, and sensitivity analyses. Panel C presents Huang’s 30-, 90-, and 180-day treatment-gap analyses, the sparse-event TZD–sulfonylurea comparison from Chen WS, and the exploratory kidney-disease subgroup from Ma SH. Filled squares indicate reported hazard ratios (HRs), and horizontal lines indicate 95% confidence intervals. Evidence roles are described in the row labels rather than encoded by marker shape. Asterisks identify estimates for which restriction to cutaneous psoriasis could not be confirmed, including those from Lee, Chiang, Mahajan, and Ma SH. The Chen WS estimate concerns a broader psoriasis-related outcome explicitly including psoriatic arthritis codes. Psoriatic arthritis-only and composite PsO/PsA outcomes are identified separately and were not treated as equivalent to cutaneous psoriasis.

†For Chen WS in panel C, “Reported new-user / TTE” reflects the design reported by the original investigators. The exposed-group index date was the first target-drug prescription, whereas the comparator-group index date was randomly assigned with reference to the exposed group’s distribution of time from diabetes diagnosis to treatment. Alignment of comparator treatment initiation with the start of follow-up was therefore not fully established. Only two outcome events occurred in the TZD group, contributing to substantial imprecision. Huang’s comparison of AGI plus metformin with metformin alone concerns a composite PsO/PsA outcome and differs in treatment intensity. Ma SH’s kidney-disease subgroup estimate does not establish effect modification because an interaction P value was not reported.

The forest axes are logarithmic and use different ranges across panels; vertical dashed lines indicate the null value of 1. Marker sizes do not represent statistical weights, and no pooled estimates were calculated. Wide confidence intervals indicate limited precision, not stronger evidence. Multiple results within a report and potentially overlapping reports do not constitute independent replications. Detailed study designs and temporal specifications are provided in Supplementary Table S4A, and additional estimates are documented in Supplementary Table S6. DPP-4i, dipeptidyl peptidase-4 inhibitor; GLP-1RA, glucagon-like peptide-1 receptor agonist; SGLT2i, sodium-glucose cotransporter-2 inhibitor; AGI, alpha-glucosidase inhibitor; TZD, thiazolidinedione; PsO, psoriasis; PsA, psoriatic arthritis; CISD, chronic inflammatory skin disease; ITT, intention-to-treat; TTE, target trial emulation.

### 2.2.5 Supplementary Figure S5. ROBINS-I traffic-light plot for report-level assessment anchors.

Supplementary Figure S5. ROBINS-I traffic-light plot for report-level assessment anchors. One assessment anchor was retained per included report for traceability. Each row corresponds to the assessment anchor documented in Supplementary Table S9A and does not necessarily represent all effect estimates reported in that study. Circles indicate low risk of bias (L, green), moderate risk of bias (M, yellow), serious risk of bias (S, red), or no information (NI, grey). No anchor received a critical judgment in any domain or overall; this absent category is not displayed. The domain-level and overall judgments are taken directly from Supplementary Table S9A; the same judgments generate the distributions in Figure 5 and Supplementary Table S9B. Overall judgments were not calculated by summing domain scores. The applicability of anchor judgments to related results and additional result-specific concerns are described in Supplementary Table S9C. D1, bias due to confounding; D2, bias in selection of participants into the study; D3, bias in classification of interventions; D4, bias due to deviations from intended interventions; D5, bias due to missing data; D6, bias in measurement of outcomes; D7, bias in selection of the reported result.

### 2.2.6 Supplementary Figure S6. Database sources and potential population overlap among the included reports.

Supplementary Figure S6. Database sources and potential population overlap among the included reports. Each row represents one report, and the horizontal categories indicate the data source or potential population-overlap group. Filled squares indicate confirmed use of the same underlying study population; filled diamonds indicate likely overlap; open circles indicate possible partial overlap; and open triangles indicate no confirmed overlap or a distinct named data source. Chiang and Lee were classified as having likely overlap. Mahajan and Ma KSK were classified as having possible overlap with other TriNetX reports. The Taiwan NHIRD reports were classified as having possible population overlap, consistent with Supplementary Table S8. Their placement in separate medication-related columns does not establish independence between these groups. These classifications describe the evidence supporting overlap rather than the number or proportion of shared participants. No confirmed overlap does not establish complete participant independence.

## 2.3 Updated literature search

An updated literature search was conducted on 11 September 2026 during manuscript revision to identify newly available evidence published in 2026. The update identified 268 records across PubMed (n = 112), Embase (n = 68), Web of Science Core Collection (n = 85), and the Cochrane Library (n = 3). After removal of 102 duplicate records, 166 records remained for title and abstract screening, of which 163 were excluded. Three reports were assessed for eligibility; two were already included in the original review, and one additional eligible report was included. The update increased the total number of included reports from 17 to 18. The scope of cross-deduplication against the initial-search database could not be confirmed; the 166 screened records should therefore not be interpreted as 166 confirmed previously unseen records. The updated selection process is shown in Supplementary Figure S7.

### 2.3.1 Supplementary Figure S7. Flow diagram of the updated literature search and study selection conducted during manuscript revision.

Supplementary Figure S7. Updated literature search and study selection during manuscript revision. The search was conducted on 11 September 2026 using the database-specific search strategies provided in Supplementary Table S1, with publication year restricted to 2026. A total of 268 records were identified from PubMed (n = 112), Embase (n = 68), Web of Science Core Collection (n = 85), and the Cochrane Library (n = 3). Following removal of 102 duplicate records, 166 records remained for title and abstract screening, of which 163 were excluded. Three reports underwent eligibility assessment; two were already included in the original review and therefore did not contribute additional reports. One newly eligible report was added, increasing the total from 17 to 18 included reports. The scope of cross-deduplication against the initial-search database could not be confirmed. Accordingly, the 166 screened records should not be interpreted as 166 confirmed unique records newly identified since the initial search. This diagram describes the revision-stage update; the initial selection process is presented in Figure 1.
